# Supplementary material for: Differentiation‐related epigenomic changes define clinically distinct keratinocyte cancer subclasses
Source: Mol Syst Biol. 2022 Sep 19;18(9):e11073. doi: 10.15252/msb.202211073 (PMC9484266; doi:10.15252/msb.202211073)
Supplement: Supplementary file 1 — Appendix [file MSB-18-e11073-s005.pdf]

Differentiation-related epigenomic changes define clinically distinct keratinocyte cancer subclasses.

Llorenç Solé-Boldo[1], Günter Raddatz[1], Julian Gutekunst[1], Oliver Gilliam[1], Felix Bormann[1], Michelle S. Liberio[2], Daniel Hasche[3], Wiebke Antonopoulos[4,5], Jan-Philipp Mallm[2,6], Anke S. Lonsdorf[7], Manuel Rodríguez-Paredes[1,8*] & Frank Lyko[1*]

[1]Division of Epigenetics, DKFZ-ZMBH Alliance, German Cancer Research Center, 69120 Heidelberg, Germany; [2]Single-cell Open Lab, German Cancer Research Center and Bioquant, 69120 Heidelberg, Germany; [3]Division of Viral Transformation Mechanisms, German Cancer Research Center, 69120 Heidelberg, Germany; [4]Tissue Bank of the National Center for Tumor Diseases (NCT), 69120 Heidelberg, Germany; [5]Institute of Pathology, Heidelberg University Hospital, 69120 Heidelberg, Germany; [6]Division of Chromatin Networks, German Cancer Research Center and Bioquant, 69120 Heidelberg, Germany; [7]Department of Dermatology, University Hospital, Ruprecht-Karls University of Heidelberg, 69120 Heidelberg, Germany; [8]Institute of Toxicology, University Medical Center Mainz, Johannes Gutenberg University, 55131 Mainz, Germany.

*Equal contribution

Corresponding authors: Manuel Rodríguez-Paredes (m.rodriguez@dkfz.de, +49 6221424631)

Frank Lyko (f.lyko@dkfz.de, +49 6221423800)

Table of Contents

Appendix Figure S1. Single-cell transcriptomics of healthy human epidermis identifies archetypical keratinocyte populations. 3

Appendix Figure S2. Main genes involved in keratinocyte differentiation identified by RNA velocity. 4

Appendix Figure S3. Tumor subclasses do not display differences in sample purity. 5

Appendix Figure S4. Single-cell methylation of human epidermis shows variable methylomes. . 6

Appendix Figure S6. Illustrative examples of FFPE tumor isolation by laser microdissection. 8

Appendix Figure S7. Comparison between epidermal differentiation-related regulatory regions identified in vitro and in vivo. 9

Appendix Table S1. Sequencing primers for sci-MET. 10

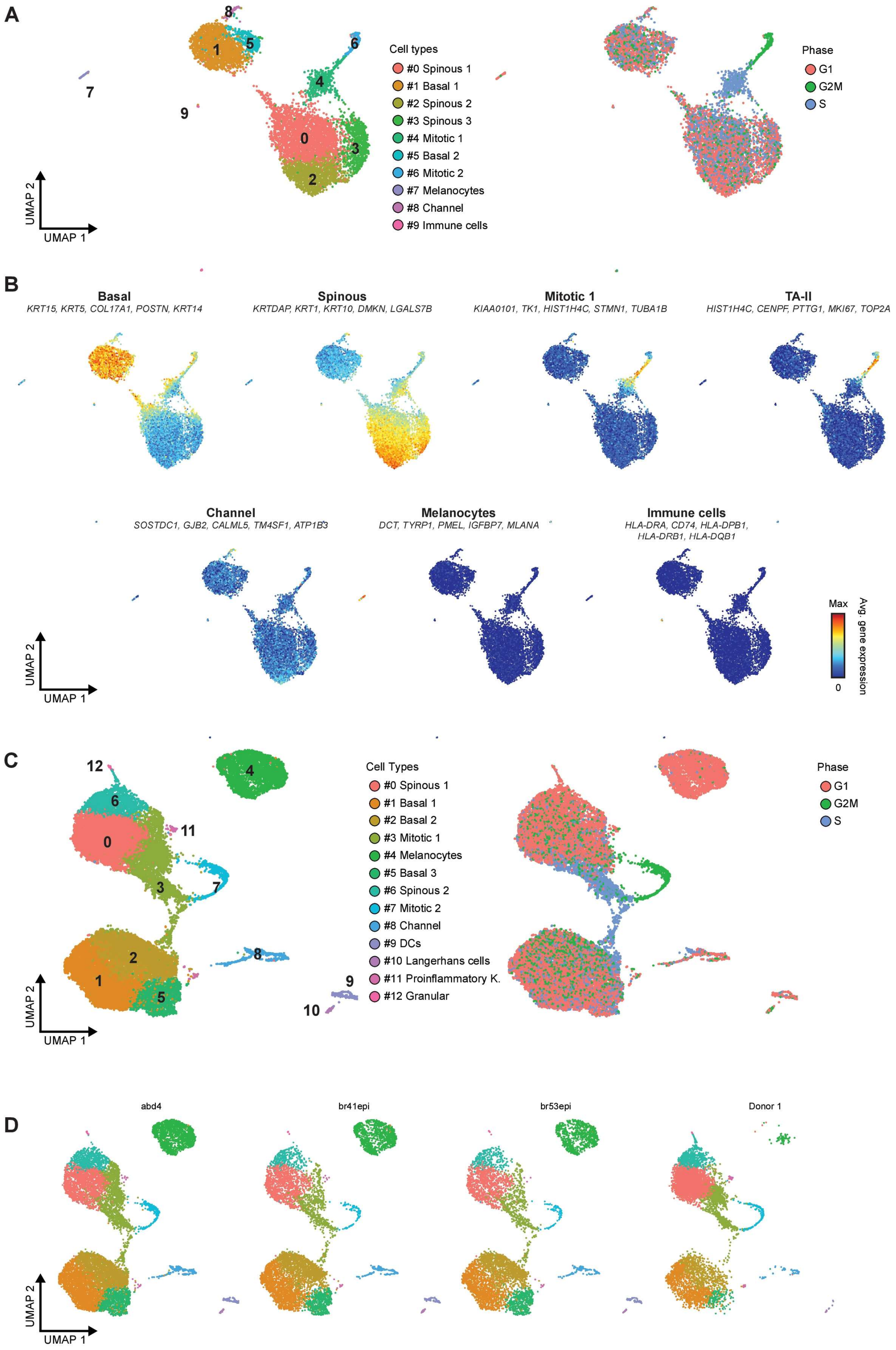
A
Cell types
#0 Spinous 1
#1 Basal 1
#2 Spinous 2
#3 Spinous 3
#4 Mitotic 1
#5 Basal 2
#6 Mitotic 2
#7 Melanocytes
#8 Channel
#9 Immune cells
Phase
G1
G2M
S
UMAP 1
UMAP 2
B
Basal
KRT15, KRT5, COL17A1, POSTN, KRT14
Spinous
KRTDAP, KRT1, KRT10, DMKN, LGALS7B
Mitotic 1
KIAA0101, TK1, HIST1H4C, STMN1, TUBA1B
TA-II
HIST1H4C, CENPF, PTTG1, MKI67, TOP2A
Channel
SOSTDC1, GJB2, CALML5, TM4SF1, ATP1B3
Melanocytes
DCT, TYRP1, PMEL, IGFBP7, MLANA
Immune cells
HLA-DRA, CD74, HLA-DPB1, HLA-DRB1, HLA-DQB1
Max
0
Avg. gene expression
C
Cell Types
#0 Spinous 1
#1 Basal 1
#2 Basal 2
#3 Mitotic 1
#4 Melanocytes
#5 Basal 3
#6 Spinous 2
#7 Mitotic 2
#8 Channel
#9 DCs
#10 Langerhans cells
#11 Proinflammatory K.
#12 Granular
Phase
G1
G2M
S
D
abd4
br41epi
br53epi
Donor 1

Appendix Figure S1. Single-cell transcriptomics of healthy human epidermis identifies archetypical keratinocyte populations.

A UMAP plot depicts 7,143 single-cell transcriptomes from a human healthy epidermis sample, generated in-house. Each cell is shown as a dot, and coloring is according to unsupervised clustering performed by Seurat (left) or cell cycle phase (right).

B UMAP plots show the average expression of the most representative genes of well-known human epidermal cell populations. Red indicates maximum average expression and blue indicates low or no expression in log-normalized UMI counts.

C UMAP plot depicts 32,272 single-cell transcriptomes from the integrated dataset comprising four healthy epidermal samples. Each cell is shown as a dot and coloring is according to unsupervised clustering performed by Seurat (left) or cell cycle phase (right).

D UMAP plots depict the contribution of each sample to the integrated dataset. Samples abd4, br41epi and br53epi correspond to the dataset published in Cheng *et al.*(Cheng *et al*, 2018) while Donor 1 corresponds to the in-house dataset.

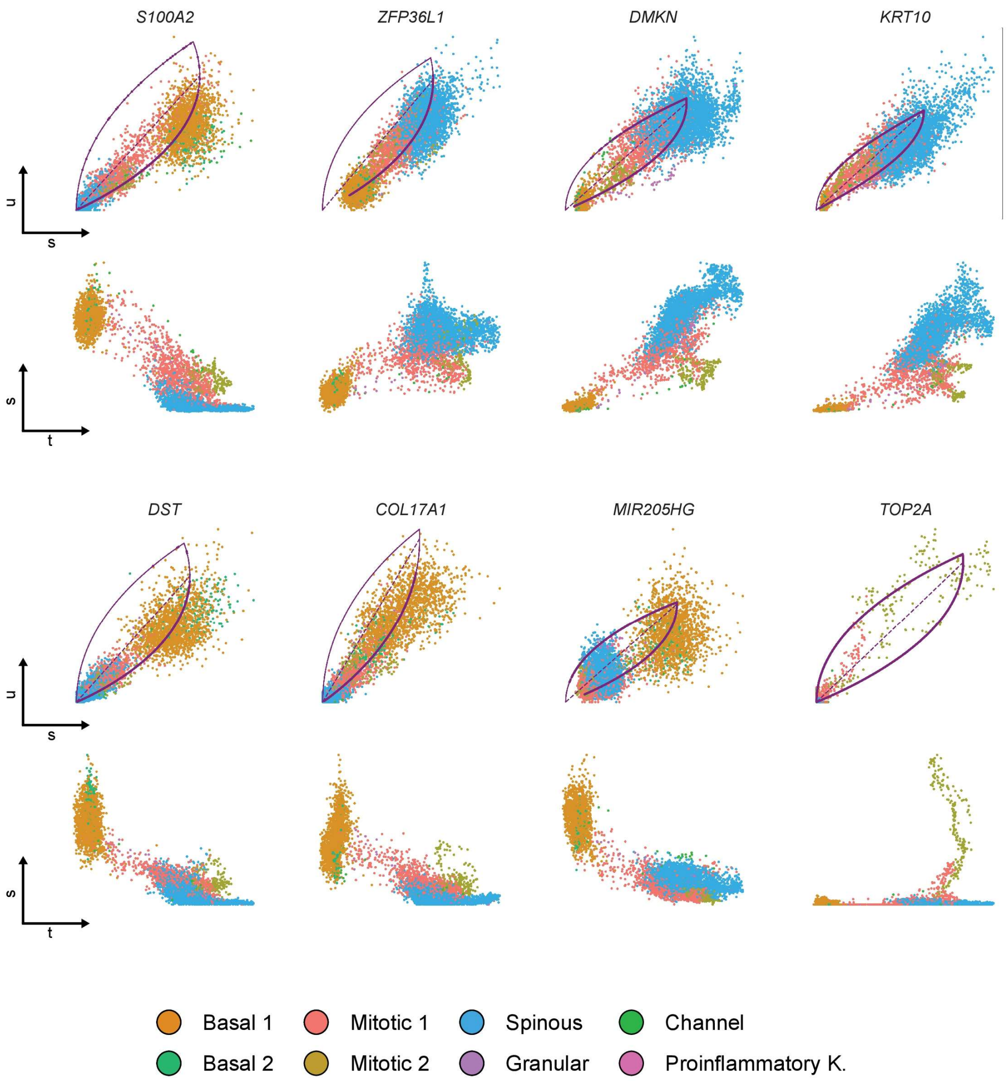

Appendix Figure S2. Main genes involved in keratinocyte differentiation identified by RNA velocity. Phase portraits (upper) and expression dynamics along latent time (lower) of the top eight putative driver genes of keratinocyte differentiation, as calculated by the dynamic model of scVelo(Bergen *et al*, 2020). Coloring is according to the defined keratinocyte populations. Bold lines depict the inferred splicing dynamics, while the dotted lines depict the transcriptional steady state. u: unspliced, s: spliced, t: latent time.

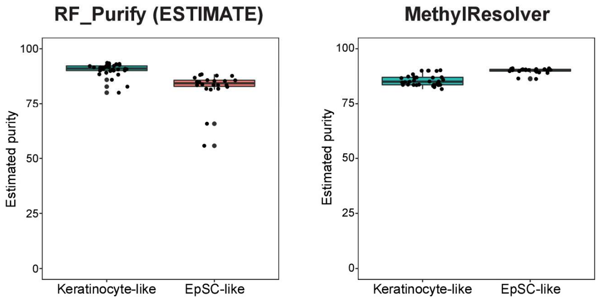

Appendix Figure S3. Tumor subclasses do not display differences in sample purity. Boxplot showing the tumor purity of the 55 AK/cSCC samples, as estimated by RF_Purify (ESTIMATE method) and MethylResolver.

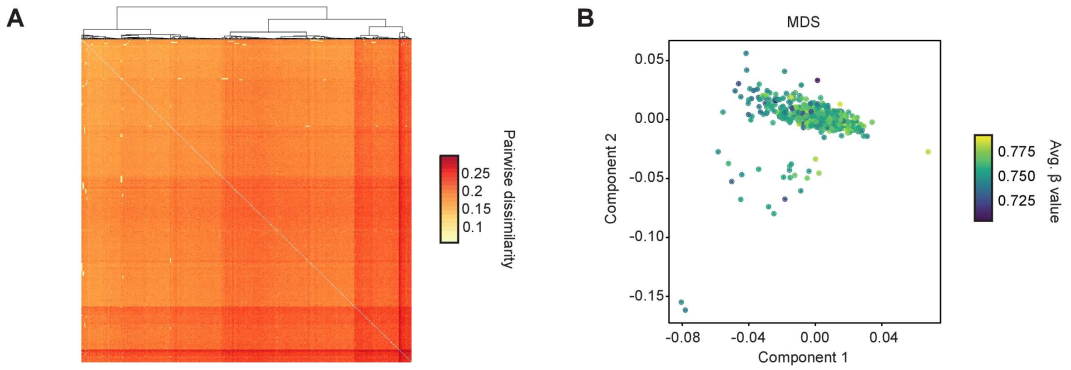

Appendix Figure S4. Single-cell methylation of human epidermis shows variable methylomes.

A Pairwise dissimilarity matrix between the 554 single-cell methylomes obtained from healthy human epidermis.

B Multidimensional scaling (MDS) analysis of the 554 single-cell methylomes obtained from healthy human epidermis and colored by average methylation content per cell.

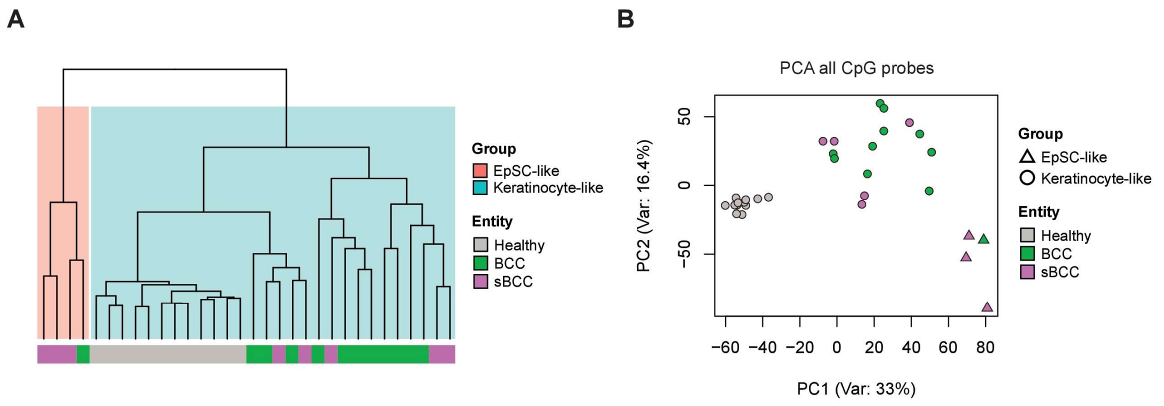

Appendix Figure S5. Combined analysis of BCC and sBCC tumor samples.

A Heatmap displaying the unsupervised hierarchical clustering of the 14 BCC (11 BCC and three sBCC) samples from our dataset and five sBCC from Sand *et al*.(Sand *et al*, 2019) based on the methylation patterns at differentiated keratinocyte-specific peaks. Healthy epidermal samples (n=12) were included as controls.

B PCA performed with 12 healthy epidermis, 11 BCC and eight sBCC samples using all CpG probes retained after filtering. Coloring is according to sample type and shape is according to cell-of-origin subclass. BCC: basal cell carcinoma, sBCC: sclerodermiform basal cell carcinoma.

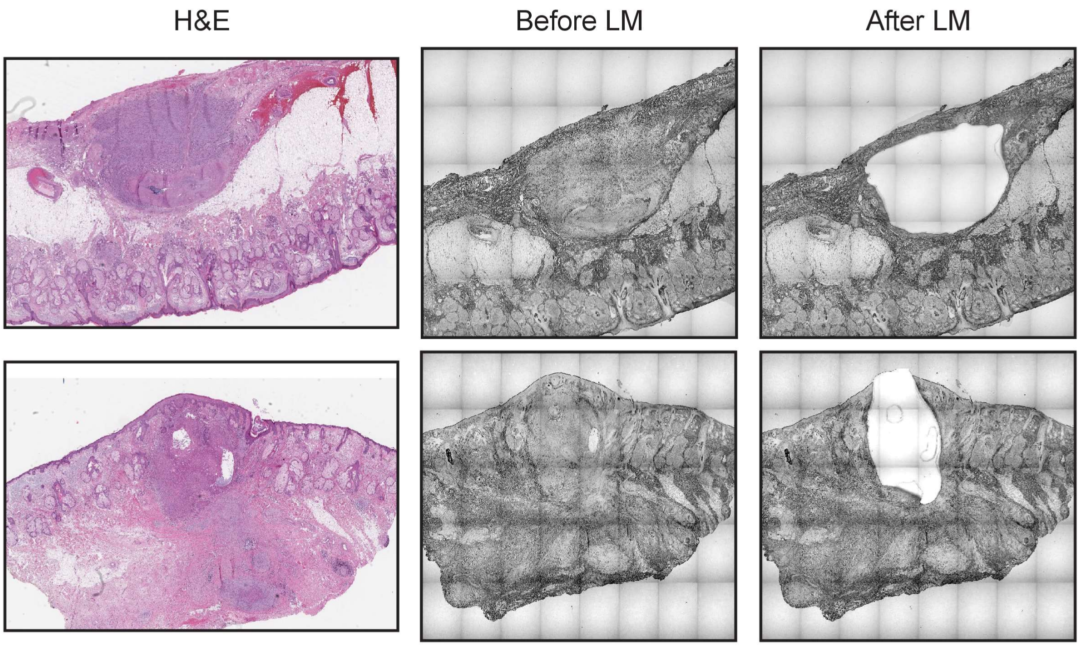

Appendix Figure S6. Illustrative examples of FFPE tumor isolation by laser microdissection. For each example, a hematoxylin and eosin (H&E) staining is shown (left), as well as an image of the tumor section before (middle) and after (right) laser microdissection. Images were taken at 5x original magnification. H&E: hematoxylin and eosin, LM: laser microdissection.

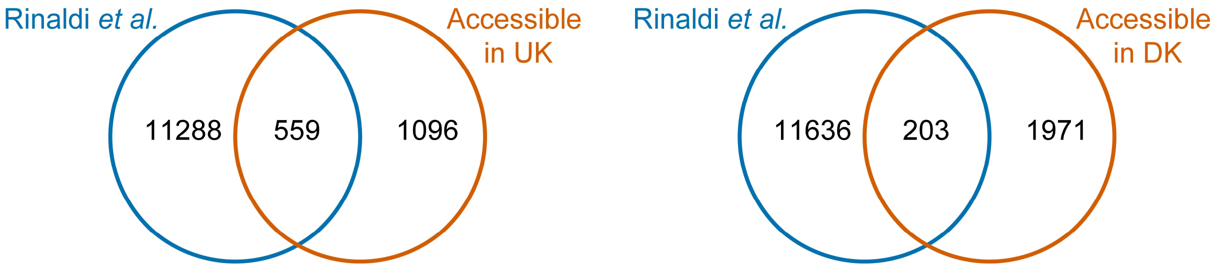

Appendix Figure S7. Comparison between epidermal differentiation-related regulatory regions identified in vitro and in vivo. Venn diagram comparing the set of undifferentiated and differentiated-specific peaks identified in our *in vivo* scATAC-seq data and a previously published set of enhancers found to be lost upon epidermal differentiation (Rinaldi *et al*, 2016), identified in upon calcium-induced differentiation of EpSC *in vitro*. DK: differentiated keratinocytes; UK: undifferentiated keratinocytes.

Appendix Table S1. Sequencing primers for sci-MET. Primer sequences for sequencing of sci-MET libraries. +: Locked Nucleic Acid (LNA) modification.

Name	Sequence
sciMET_Read1	+TGGT+AGAG+AGGG+TGAGATGTGTATAAGAGATAG
sciMET_Index1	AGATCGGAAGAGCACACGTCTGAACTCCAGTCAC
sciMET_Index2	+CTA+TCT+CTT+ATA+CAC+ATC+TCACCCTCTCTACCA
PhiX_Read1	ACACTCTTTCCCTACACGACGCTCTTCCGATCT
PhiX_Index1	AGATCGGAAGAGCGTCGTGTAGGGAAAGAGTGT

References

Bergen V, Lange M, Peidli S, Wolf FA & Theis FJ (2020) Generalizing RNA velocity to transient cell states through dynamical modeling. *Nat Biotechnol* 38: 1408–1414

Cheng JB, Sedgewick AJ, Finnegan AI, Benz SC, Song JS & Cho RJ (2018) Transcriptional Programming of Normal and Inflamed Human Epidermis at Single-Cell Resolution. *Cell Rep* 25: 871–883

Rinaldi L, Datta D, Serrat J, Morey L, Solanas G, Avgustinova A, Blanco E, Pons JI, Matallanas D, von Kriegsheim A, *et al* (2016) Dnmt3a and Dnmt3b Associate with Enhancers to Regulate Human Epidermal Stem Cell Homeostasis. *Cell Stem Cell* 19: 491–501

Sand M, Hessam ABDSTGS, Becker JC, Meyer EST & Bechara FG (2019) Dicer sequencing, whole genome methylation profiling, mRNA and smallRNA sequencing analysis in basal cell carcinoma. *Cell Physiol Biochem* 53: 760–773
