# Supplementary material for: Differentiation‐related epigenomic changes define clinically distinct keratinocyte cancer subclasses
Source: Mol Syst Biol. 2022 Sep 19;18(9):e11073. doi: 10.15252/msb.202211073 (PMC9484266; doi:10.15252/msb.202211073)
Supplement: Supplementary file 2 — Expanded View Figures PDF [file MSB-18-e11073-s002.pdf]

Expanded View Figures

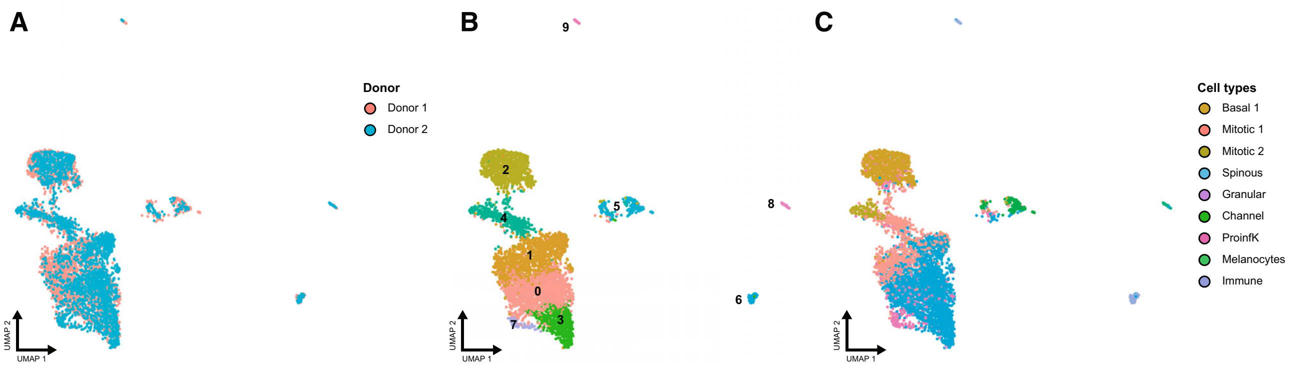

Figure EV1. Unsupervised clustering of the integrated single-cell multi-omics dataset.

A–C Joint UMAP plot depicting both scATAC-seq and scRNA-seq data from 5,565 cells from sun-protected human epidermis (*n* = 2) after data integration. Coloring is according to donor (A), unsupervised clustering based on gene expression (B), and cell-type annotation based on the reference scRNA-seq dataset (C).

© 2022 The Authors

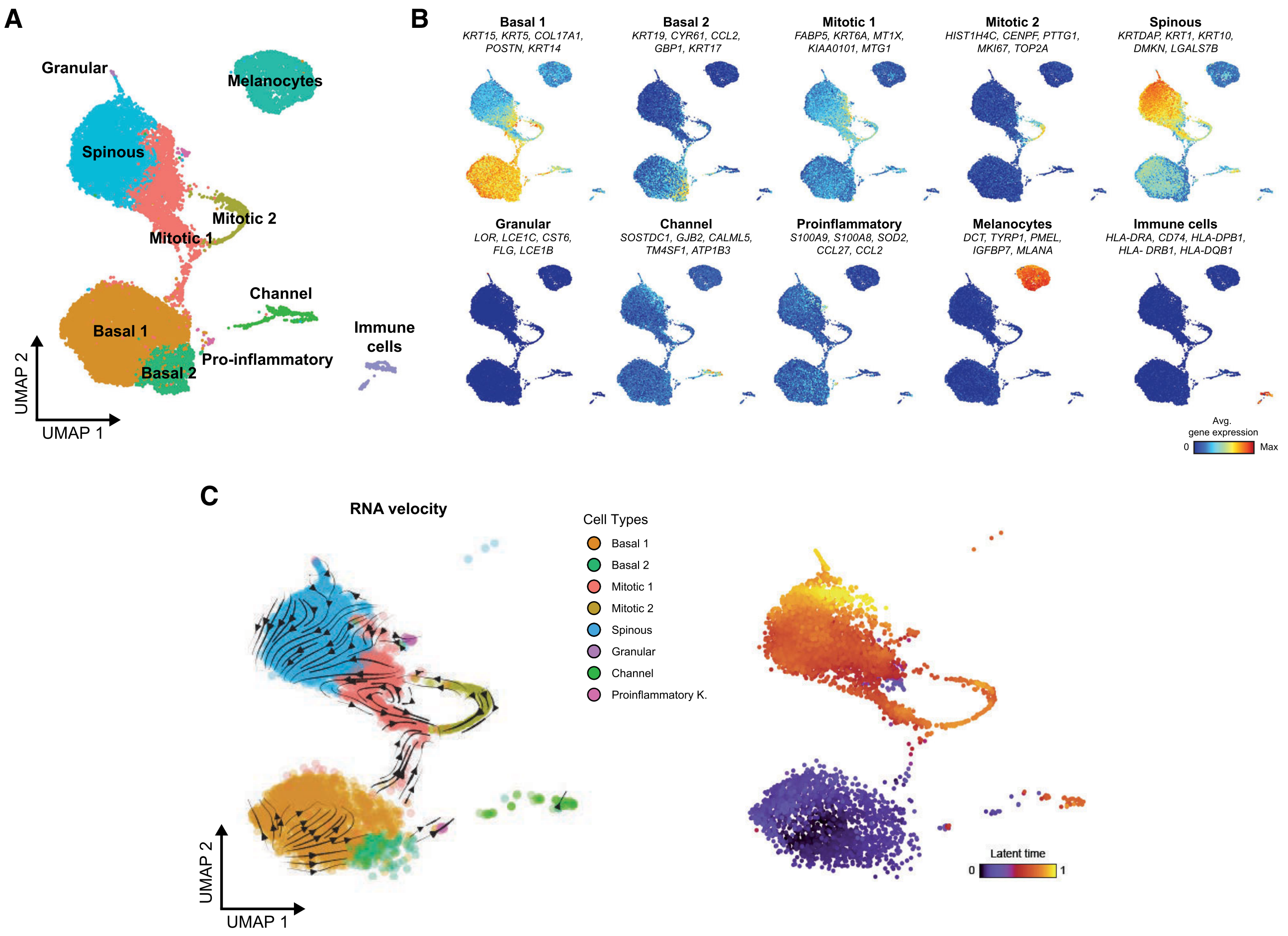

Figure EV2. Single-cell RNA sequencing analysis of the human epidermis.

A Uniform manifold approximation and projection (UMAP) plot depicting single-cell transcriptomes from healthy sun-protected human epidermis (n = 4). Each dot represents a single cell (n = 32,272). Colors depict the six archetypical keratinocyte populations described in the text, as well as other minority cell types (Cheng *et al*, 2018a; Data ref: Cheng *et al*, 2018b).

B Average expression of the top five gene markers defining each cell population projected on the UMAP plot. Red indicates maximum average gene expression, while blue indicates low or no expression of a particular set of genes in log-normalized UMI counts.

C Left: RNA velocities calculated using the 7,068 keratinocytes from the in-house generated dataset of healthy human epidermis, projected onto the UMAP embedding. Right: UMAP plot displaying the latent time calculated by scVelo.

Figure EV3. Cell-of-origin-based tumor stratification strategy can be expanded to other epidermal tumor entities.

A–E Heatmaps displaying unsupervised hierarchical clustering based on the methylation patterns at differentiated keratinocyte-specific peaks of (A) 102 epidermal tumors and healthy controls; (B) Five sBCC samples from Sand *et al* (2019a), Data ref: Sand *et al* (2019b); (C) 12 averaged VV samples from Al-Eitan *et al* (2020) (D) Five AK and 18 cSCC samples from Hervás-Marín *et al* (2019a), Data ref: Hervás-Marín *et al* (2019b); (E) Eight cSCC metastases and three primary metastatic cSCC, always together with the 12 healthy samples from our cohort. Each row represents the average methylation value of all CpGs contained in a particular peak. Heatmaps correspond to the dendrograms shown in Fig 5A–E. AK: actinic keratosis, BCC: basal cell carcinoma, BD: Bowen's disease, cSCC: cutaneous squamous cell carcinoma, sBCC: sclerodermiform basal cell carcinoma, SK: seborrheic keratosis, VV: verruca vulgaris.

 © 2022 The Authors

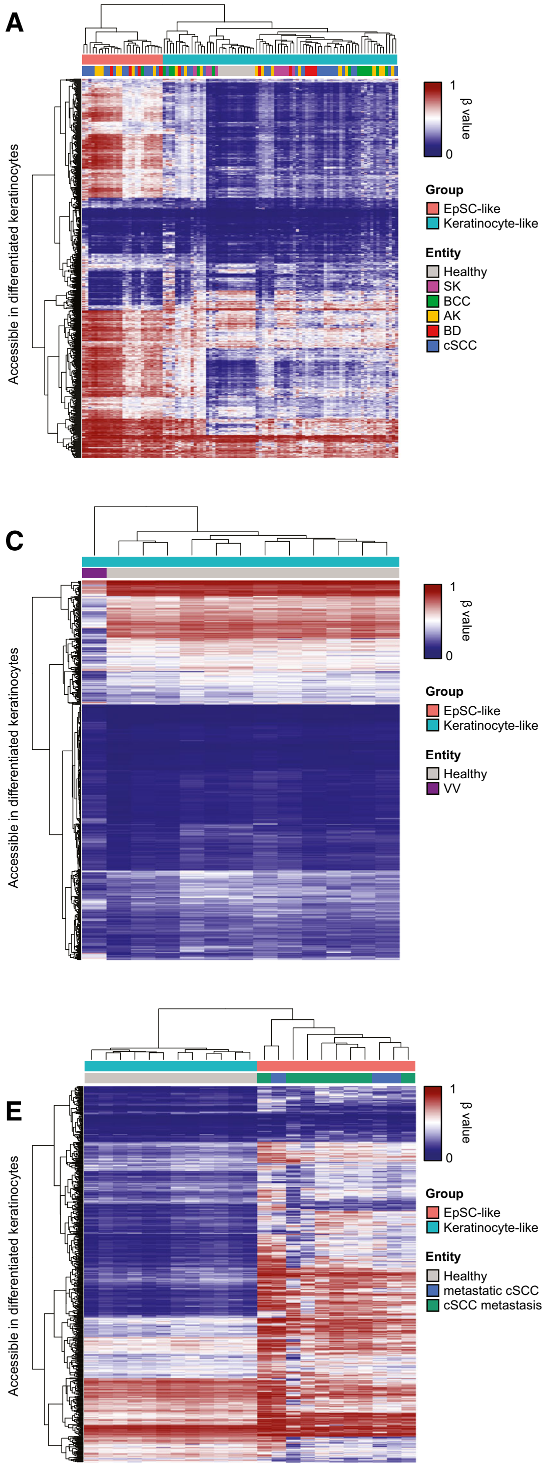
A
B
C
D
E
Accessible in differentiated keratinocytes
β value
1
0
Group
EpSC-like
Keratinocyte-like
Entity
Healthy
SK
BCC
AK
BD
cSCC
sBCC
VV
metastatic cSCC
cSCC metastasis

Figure EV3.

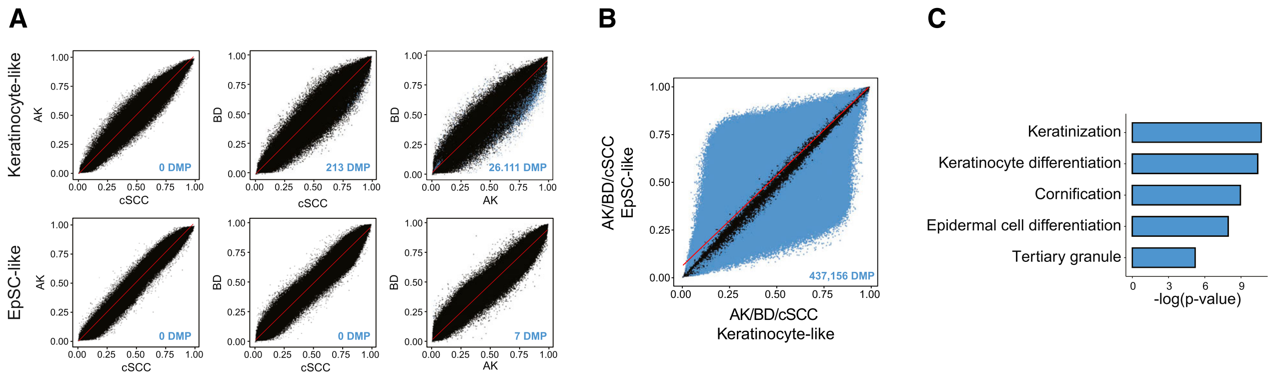

Figure EV4. cSCC and precursor lesions from the same cell-of-origin subclass display almost identical methylomes.

A Scatter plots of pairwise comparisons between AK, BD, and cSCC methylomes from the keratinocyte-like (upper) or EpSC-like (lower) subclass.

B Scatter plot comparing AK, BD, and cSCC methylomes from each cell-of-origin subclass as a unique entity.

C Top five enriched Gene Ontology (GO) terms using genes with differentially methylated promoter regions between AK/BD/cSCC samples from distinct cell-of-origin subclasses.

Data information: In the scatter plots, significantly differentially methylated CpG probes (P-value < 0.05, F-test) are depicted in blue. AK: actinic keratosis, BD: Bowen's disease, cSCC: cutaneous squamous cell carcinoma, DMP: differentially methylated probes.

 © 2022 The Authors
