# Supplementary material for: Differentiation‐related epigenomic changes define clinically distinct keratinocyte cancer subclasses
Source: Mol Syst Biol. 2022 Sep 19;18(9):e11073. doi: 10.15252/msb.202211073 (PMC9484266; doi:10.15252/msb.202211073)
Supplement: Supplementary file 6 — PDF+ [file MSB-18-e11073-s001.pdf]

Article

TRANSPARENT PROCESS
OPEN ACCESS

molecular systems biology

Differentiation-related epigenomic changes define clinically distinct keratinocyte cancer subclasses

Llorenç Solé-Boldo[1], Günter Raddatz[1], Julian Gutekunst[1], Oliver Gilliam[1], Felix Bormann[1], Michelle S Liberio[2], Daniel Hasche[3], Wiebke Antonopoulos[4,5], Jan-Philipp Mallm[2,6], Anke S Lonsdorf[7], Manuel Rodríguez-Paredes[1,8,*,†] & Frank Lyko[1,**,†]

Abstract

Keratinocyte cancers (KC) are the most prevalent malignancies in fair-skinned populations, posing a significant medical and economic burden to health systems. KC originate in the epidermis and mainly comprise basal cell carcinoma (BCC) and cutaneous squamous cell carcinoma (cSCC). Here, we combined single-cell multi-omics, transcriptomics, and methylomics to investigate the epigenomic dynamics during epidermal differentiation. We identified ~3,800 differentially accessible regions between undifferentiated and differentiated keratinocytes, corresponding to regulatory regions associated with key transcription factors. DNA methylation at these regions defined AK/cSCC subtypes with epidermal stem cell- or keratinocyte-like features. Using cell-type deconvolution tools and integration of bulk and single-cell methylomes, we demonstrate that these subclasses are consistent with distinct cells-of-origin. Further characterization of the phenotypic traits of the subclasses and the study of additional unstratified KC entities uncovered distinct clinical features for the subclasses, linking invasive and metastatic KC cases with undifferentiated cells-of-origin. Our study provides a thorough characterization of the epigenomic dynamics underlying human keratinocyte differentiation and uncovers novel links between KC cells-of-origin and their prognosis.

Keywords cell-of-origin; epigenetics; keratinocyte cancers; single-cell
Subject Categories Cancer; Chromatin, Transcription & Genomics; Skin
DOI 10.15252/msb.202211073 | Received 13 April 2022 | Revised 17 August 2022 | Accepted 30 August 2022
Mol Syst Biol. (2022) 18: e11073

Introduction

The epidermis constitutes the first line of defense of the human body against environmental damage. This stratified squamous epithelium is mainly composed of keratinocytes, which arise from epidermal stem cells (EpSCs) located at the basal layer of the epidermis (Gonzales & Fuchs, 2017; Moreci & Lechler, 2020). As EpSCs start differentiating, they detach from the basement membrane and migrate upwards, resulting in distinct differentiated keratinocyte populations (i.e., spinous, granular, and cornified) (Gonzales & Fuchs, 2017; Moreci & Lechler, 2020). Terminally differentiated keratinocytes are continuously desquamated. As such, the homeostatic epidermis is subjected to a constant turnover, which is regulated by a fine-tuned balance between self-renewal and differentiation (Blanpain & Fuchs, 2009).

Keratinocyte cancers (KC), also known as non-melanoma skin cancers (NMSC), originate from epidermal keratinocytes. They represent the most common malignancies worldwide in the fair-skinned population, with an incidence 20 times higher than that of melanoma, the other major skin cancer (Apalla *et al*, 2017b; Fitzmaurice *et al*, 2019; Stang *et al*, 2019). The incidence of KC has alarmingly risen over the last decade, with an increase of ~33% in the total number of cases worldwide between 2007 and 2017 (Fitzmaurice *et al*, 2019). These numbers illustrate why, despite a lower mortality rate, KC are associated with significant morbidity and a heavy burden on public health systems (Mudigonda *et al*, 2010; Apalla *et al*, 2017b; Fitzmaurice *et al*, 2019). Two distinct malignancies account for 99% of all KC: basal cell carcinoma (BCC) and cutaneous squamous cell carcinoma (cSCC) (Apalla *et al*, 2017a; Bartoš & Kullová, 2018). Even though cSCC represents only 20% of KC cases, it accounts for the vast majority of deaths associated with such malignancies, as about 5% of the tumors metastasize, with a mortality rate exceeding 70% (Ratushny *et al*, 2012; Burton *et al*,

1 Division of Epigenetics, DKFZ-ZMBH Alliance, German Cancer Research Center, Heidelberg, Germany
2 Single-cell Open Lab, German Cancer Research Center and Bioquant, Heidelberg, Germany
3 Division of Viral Transformation Mechanisms, German Cancer Research Center, Heidelberg, Germany
4 Tissue Bank of the National Center for Tumor Diseases (NCT), Heidelberg, Germany
5 Institute of Pathology, Heidelberg University Hospital, Heidelberg, Germany
6 Division of Chromatin Networks, German Cancer Research Center and Bioquant, Heidelberg, Germany
7 Department of Dermatology, University Hospital, Ruprecht-Karls University of Heidelberg, Heidelberg, Germany
8 Institute of Toxicology, University Medical Center Mainz, Johannes Gutenberg University, Mainz, Germany
*Corresponding author. Tel: +49 6221424631; E-mail: m.rodriguez@dkfz.de
**Corresponding author. Tel: +49 6221423800; E-mail: f.lyko@dkfz.de
†These authors contributed equally to this work

© 2022 The Authors. Published under the terms of the CC BY 4.0 license.

2016). In contrast, the estimated metastatic potential of BCC is less than 0.05% (Apalla *et al*, 2017a). Most invasive cSCCs arise either from a precancerous dysplasia known as actinic keratosis (AK) or from an *in situ* carcinoma known as Bowen's disease (BD), with a progression rate of 0.025-16% and 3-5% per year and event, respectively (Ratushny *et al*, 2012; Burton *et al*, 2016). However, the molecular mechanisms underlying their progression to invasive cSCC remain largely unknown.

DNA methylation is a dynamic epigenetic modification that mainly occurs in the context of CpG dinucleotides at the carbon-5 position of cytosines (Lyko, 2018). Catalyzed by a set of three methyltransferases (DNMT1, DNMT3A, and DNMT3B), it has a strong influence on gene expression and other essential genetic functions (Lyko, 2018). Consequently, DNA methylation is essential for the establishment and maintenance of cellular identity (Lyko, 2018; Greenberg & Bourc'his, 2019). Disruption of normal DNA methylation patterns is currently considered a hallmark of cancer, which presents a characteristic genome-wide hypomethylation and regional hypermethylation (Jones & Baylin, 2007). Importantly, tumor methylomes not only include cancer-specific methylation changes, but also partially maintain the DNA methylation patterns of their tumor-initiating cell (Kulis *et al*, 2013; Moran *et al*, 2016). In fact, a systematic study concluded that cell-of-origin-related patterns are the main variable influencing tumor stratification in many tumor entities (Hoadley *et al*, 2018).

Epidermal differentiation has been associated with dynamic changes in DNA methylation. In mice, keratinocyte differentiation was associated with a general loss of DNA methylation at lineage-specific regulatory elements, while methylation gains occurred at regulatory regions of other lineages (Bock *et al*, 2012). Similarly, a loss of DNA methylation in the promoter region of roughly 60% of the genes induced upon keratinocyte differentiation *in vitro* has been observed in humans (Sen *et al*, 2010). In agreement with these findings, we have previously identified two subclasses of AK and cSCC based on their methylation patterns and that we interpreted to arise from keratinocytes at two distinct epidermal differentiation stages: one more closely related to the EpSCs and one to a more differentiated keratinocyte (Rodríguez-Paredes *et al*, 2018a). However, direct proof for this interpretation has been lacking and the subclasses were not characterized in detail.

Here, we performed an integrated analysis of the chromatin dynamics associated with human epidermal differentiation using single-cell multi-omics and transcriptomics approaches. We identified more than 3,800 differentially accessible regions between undifferentiated and terminally differentiated keratinocytes. Further characterization of these regions revealed that they comprised regulatory regions associated with known but also novel epidermal differentiation transcription factors. Tumor stratification based on the DNA methylation patterns found at these differentially accessible regions identified two subtypes of AK and cSCC with EpSC-like and keratinocyte-like features. Importantly, we also show for the first time DNA methylation dynamics in the human epidermis at single-cell resolution, which we studied with single-cell combinatorial indexing for methylation analysis (sci-MET) (Mulqueen *et al*, 2018), after addressing important shortcomings of the original protocol. The integrative analysis of bulk and single-cell methylation datasets, as well as the use of deconvolution tools based on scRNA-seq, provided direct evidence of the cell-of-origin interpretation of the AK/cSCC subtypes. Furthermore, epigenomic data analyses using a mitotic-like clock and the stratification of an expanded dataset, which included BCC and other yet unstratified epidermal entities, suggested a more invasive phenotype and higher metastatic potential for tumors arising from undifferentiated keratinocytes. All in all, our DNA methylation-based tumor stratification strategy may represent an important advance in the risk assessment of KC patients.

Results

Single-cell multi-omics analysis of healthy human epidermis

To investigate differentiation-related epigenomic changes in the human epidermis at the single-cell level, we used a combination of single-cell multi-omic and transcriptomic approaches. First of all, we generated a single-cell multi-omics (scATAC-seq + scRNA-seq) dataset from two sun-protected healthy epidermis samples (55 and 72 y/o, male). A total of 5,565 cells passed the quality control for both genomic layers and were integrated into a common dataset to avoid batch effects (Fig EV1A). Unsupervised clustering identified 10 cell clusters, which were visualized using a joint uniform manifold approximation and projection (UMAP) representing both gene expression and chromatin accessibility (Fig EV1B). To identify the cell identity of each cluster, we also generated a reference scRNA-seq dataset by combining our own data generated from a sun-protected healthy epidermis sample from a 30 y/o male donor (Appendix Fig S1A and B), with a matching subset of sun-protected healthy epidermis from three donors (Cheng *et al*, 2018a; Data ref: Cheng *et al*, 2018b). All four samples were obtained from the trunk area and did not display significant differences. The integrated dataset contained 32,272 high-quality cells and their unsupervised clustering defined 13 cell clusters, which comprised cells from all donors (Appendix Fig S1C and D). These included six archetypical keratinocyte populations: two basal undifferentiated populations, two mitotic clusters, and the well-differentiated spinous and granular keratinocytes (Fig EV2A and B, and Dataset EV1). Highly specialized keratinocyte populations such as channel or pro-inflammatory keratinocytes were also detected (Fig EV2A and B, and Dataset EV1). Lineage inference using RNA velocity analysis was possible with our own dataset and placed the Basal 1 population at the beginning of the differentiation process (Fig EV2C and Appendix Fig S2). The trajectory then progressed to the mitotic keratinocytes and, lastly, to the well-differentiated spinous population (Fig EV2C). Hence, these results suggest that the main EpSC population is contained in the Basal 1 cluster.

After cell annotation based on the reference scRNA-seq dataset, most keratinocyte populations identified in the scRNA-seq experiment were also detected in the multi-omics dataset (Fig 1A). Of note, our multi-omics analysis showed the expected chromatin accessibility and gene expression dynamics for several established epidermal differentiation markers. For instance, ATAC peaks associated with either the basal keratinocyte gene marker *KRT5* or the suprabasal differentiated keratinocyte gene marker *KRT10*, lost or gained accessibility as they became less or more expressed along the differentiation trajectory, respectively (Fig 1B). Consistently, we observed an increase in co-accessibility in the epidermal

 © 2022 The Authors

differentiation complex (EDC), a genomic region containing multiple genes related to terminal differentiation and cornification (Kypriotou *et al*, 2012), in terminally differentiated keratinocytes (Spinous and Granular, cluster 3) compared with basal undifferentiated keratinocytes (Basal 1, cluster 2, Fig 1C).

To further characterize differentiation-related changes occurring at the chromatin level, we compared the genome accessibility in basal undifferentiated keratinocytes (Basal 1, cluster 2) and in terminally differentiated keratinocytes (Spinous and Granular, cluster 3). This comparison identified 3,838 differentially accessible peaks, of which 1,659 were only accessible in undifferentiated keratinocytes and 2,179 were only accessible in differentiated keratinocytes (Fig 1D, Dataset EV2). Motif enrichment analysis for each set of accessible peaks identified cell-type-specific overrepresentation of transcription factor (TF) binding motifs (Fig 1D). For example, TF-binding motifs associated with key regulators of epidermal stem cell proliferation and differentiation such as TP63 (Soares & Zhou, 2018) and OVOL1 (Lee *et al*, 2014) were enriched in peaks that were

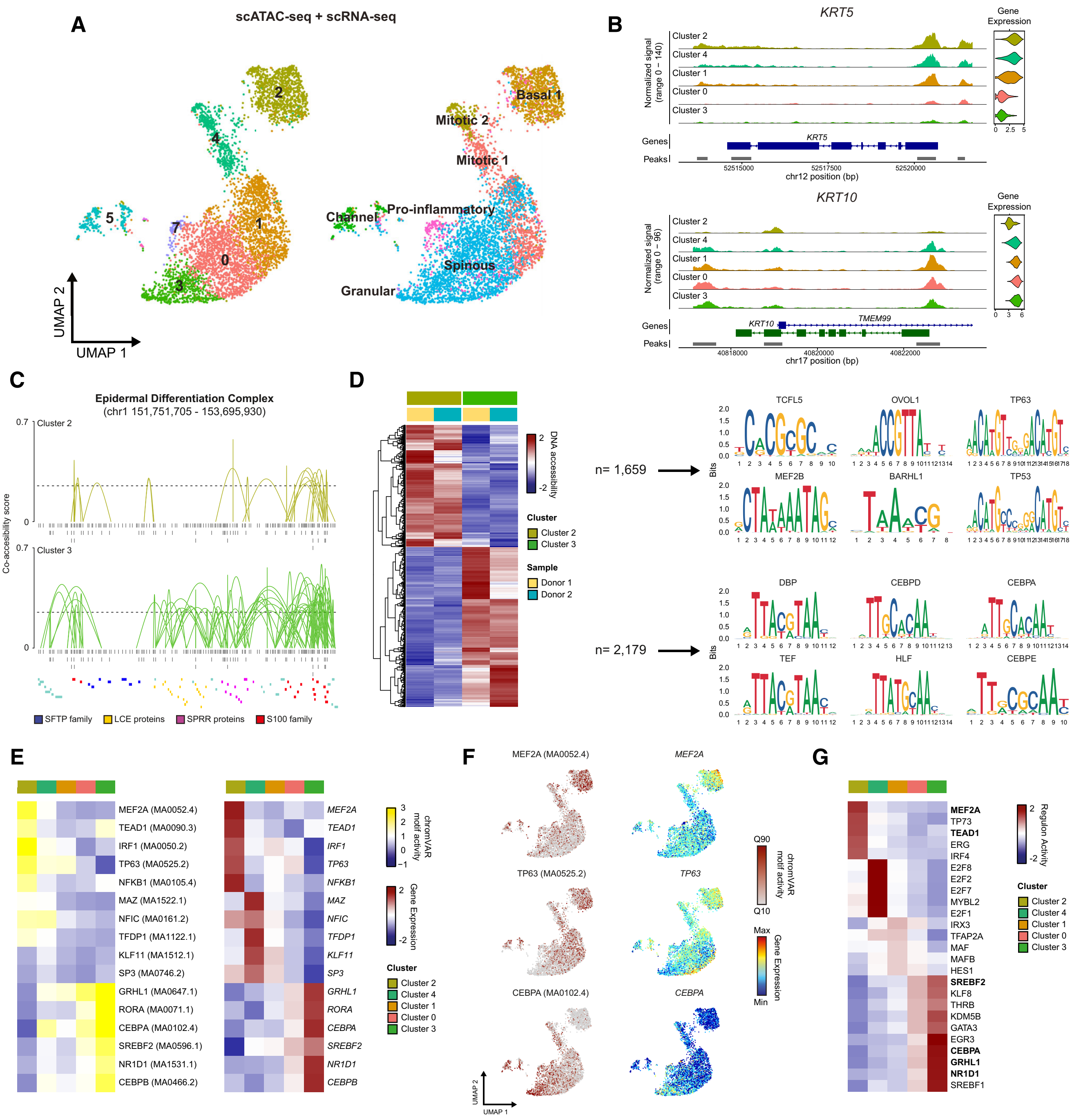

Figure 1.

◀ **Figure 1. Single-cell multi-omics characterization of human epidermal differentiation.**

A Joint UMAP plot depicting both scATAC-seq and scRNA-seq data from 5,355 keratinocytes from sun-protected human epidermis (n = 2). Color depicts the unsupervised clustering (left) as well as cell-type annotation based on the reference scRNA-seq dataset (right).

B Representative examples of chromatin accessibility and gene expression of *KRT5* and *KRT10*, two key epidermal differentiation-related genes, in the main keratinocyte populations.

C Co-accessibility at the human epidermal differentiation complex (EDC) in undifferentiated (cluster 2) and differentiated (cluster 3) keratinocytes. Only connections (arcs) with a co-accessibility score above 0.25 are plotted. Gray boxes below tracks represent scATAC-seq peaks.

D Left: Heatmap displaying the differentially accessible peaks between undifferentiated (cluster 2) and differentiated (cluster 3) keratinocytes. Number of Tn5 insertion sites in each region was scaled by row. Right: DNA sequence motifs for the top six overrepresented transcription factor (TF) motifs in undifferentiated (cluster 2) and differentiated (cluster 3) keratinocyte-specific accessible peaks.

E Heatmaps displaying the predicted top five transcription factors in each cell cluster using chromVAR motif activity (left) and gene expression (right).

F chromVAR deviations (in quantiles) and gene expression for representative enriched TF projected onto the joint UMAP plot of keratinocytes from the single-cell multi-omics dataset.

G Heatmap showing the top five active regulons in each cell cluster, based on the expression data of the multi-omics dataset. TF regulons also identified by other approaches are highlighted.

specific to undifferentiated keratinocytes (Fig 1D). In contrast, TF-binding motifs from members of the CEBP family, which are associated with terminal differentiation in keratinocytes (Borrelli *et al*, 2007; Lopez *et al*, 2009), were enriched in peaks specific to differentiated keratinocytes (Fig 1D). To refine our multimodal analysis, we then combined motif activity scores calculated using chromVAR and gene expression data in order to identify the transcription factors with specifically enriched expression and motif accessibility in each cell cluster. This identified key regulators of the basal undifferentiated keratinocytes, including MEF2A, TEAD1, IRF1, TP63, and NFKB and key transcription factors of terminally differentiated keratinocytes, including GRHL1, RORA, CEBPA, NR1D1, or SREBF2 (Fig 1E and F). While most of these transcription factors have been previously found to play important roles in epidermal differentiation (Truong *et al*, 2006; Dai *et al*, 2013; Gulati *et al*, 2013; Mlacki *et al*, 2014; Yuan *et al*, 2020), MEF2A has not yet been associated with this process. Gene regulatory networks analysis using single-cell regulatory network inference and clustering (SCENIC) (Aibar *et al*, 2017) on the transcriptomics data of our multi-omics dataset also identified MEF2A and TEAD1 as key transcription factors for undifferentiated keratinocytes, and SREBF2, CEBPA, GRHL1, and NR1D1 for differentiated keratinocytes (Fig 1G). Altogether, our multi-omics data recapitulated known accessibility and gene expression dynamics during epidermal differentiation and identified potential new key regulators, such as MEF2A.

DNA methylation at differentially accessible regions defines AK/cSCC subtypes

To investigate whether the differentially accessible peaks detected during epidermal differentiation corresponded to regulatory regions, such as gene promoters or enhancers, we made use of published ChIP-seq data for several histone marks generated on normal human epidermal keratinocytes (NHEK). Accessible peaks from basal and differentiated keratinocytes showed no enrichment for the repressive chromatin mark H3K27me3, in agreement with their open state (Fig 2A). On the contrary, differentiated keratinocyte-specific peaks showed a strong correlation with H3K27ac and H3K4me1, two histone marks that are associated with active enhancers (Creyghton *et al*, 2010) (Fig 2A). Furthermore, undifferentiated keratinocyte-specific peaks were enriched for H3K27ac and H3K4me2/me3, histone marks that are associated with gene promoters and actively transcribed regions (Bernstein *et al*, 2005; Orford *et al*, 2008) (Fig 2A). Altogether, our analyses indicate that the differentially accessible regions identified in our scATAC-seq data correspond to regulatory regions associated with key regulators of epidermal differentiation.

DNA methylation cooperates with chromatin accessibility to establish and maintain cellular identity (Guo *et al*, 2016; Li *et al*, 2021). Furthermore, DNA methylation patterns at regulatory regions have been used to define the cellular origin of several human cancer types (Kulis *et al*, 2013; Moran *et al*, 2016; Hoadley *et al*, 2018). To assess whether the methylation patterns at the differentially accessible regions between undifferentiated and differentiated keratinocytes would be informative for identifying the cellular origin of epidermal tumors, we extracted the CpGs located in the 3,838 differentially accessible peaks and that can be interrogated with probes on the Infinium EPIC array. This identified 2,925 CpG probes located in undifferentiated keratinocyte-specific peaks and 1,426 CpG probes located in differentiated keratinocyte-specific peaks. These probes covered 914 and 864 peaks accessible exclusively in undifferentiated or differentiated keratinocytes, respectively. In agreement with the histone modifications landscape of each set of accessible regions, the CpGs located in undifferentiated keratinocyte-specific peaks were mostly located in promoter-associated CpG islands while the CpGs located in differentiated keratinocyte-specific peaks were mostly located in OpenSea regions, which are often associated with enhancers (Fig 2B). We then combined 21 newly generated AK and cSCC methylomes with a published dataset comprising healthy, AK and cSCC epidermis samples (Rodríguez-Paredes *et al*, 2018a; Data ref: Rodríguez-Paredes *et al*, 2018b). Unsupervised clustering of all 12 healthy, 20 AK and 35 cSCC epidermal samples based on the methylation patterns of either the 914 undifferentiated keratinocyte-specific peaks or the 864 differentiated keratinocyte-specific peaks clearly stratified the AK and cSCC methylomes into two groups, one with EpSC-like features and another one with keratinocyte-like features (Fig 2C). The two subclasses were also clearly separated in a Principal Component Analysis (PCA) based on all CpG probes (Fig 2D). Importantly, this separation was not related to differences in sample purity, as both subclasses showed a very high degree of tumor cell purity (Appendix Fig S3).

Single-cell methylation analysis of keratinocyte differentiation

To further refine the cells-of-origin of AK/cSCC, we combined these bulk methylation datasets with single-cell methylation data from

 © 2022 The Authors

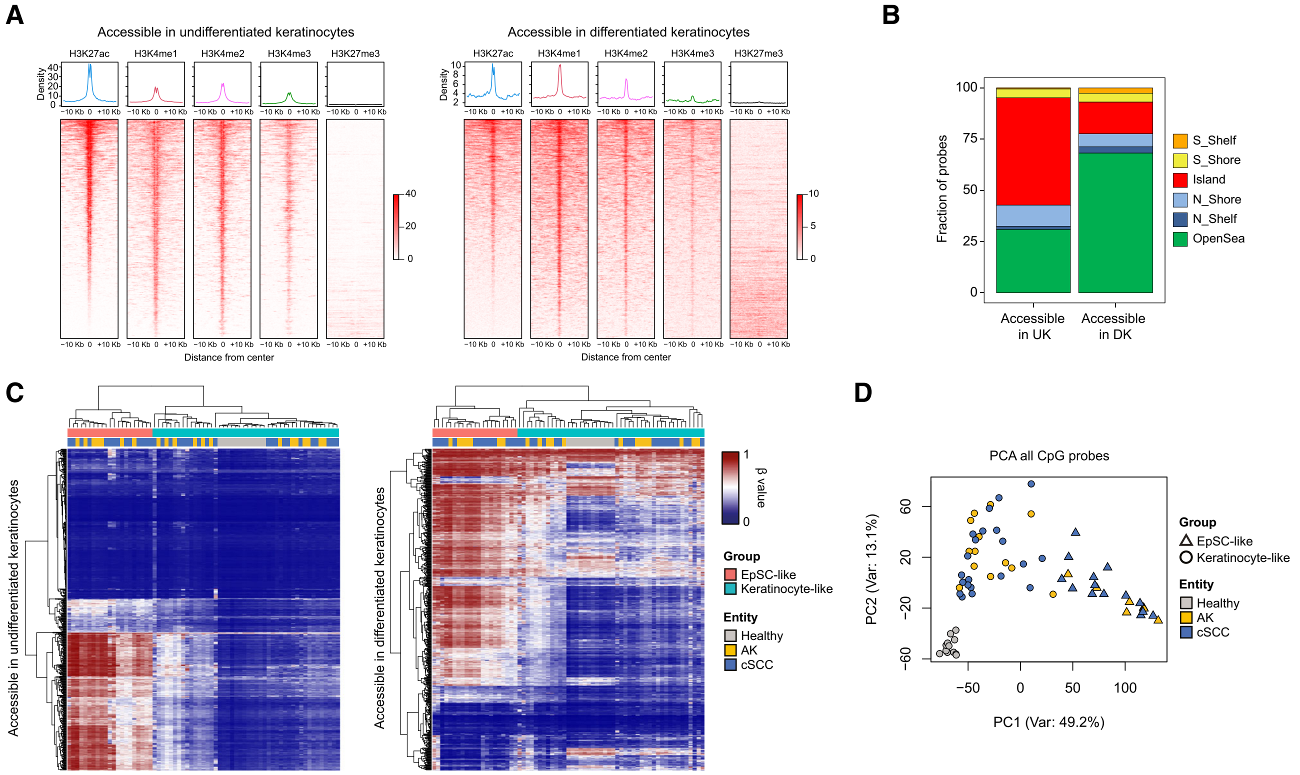

Figure 2. Epidermal differentiation-specific accessible regions define AK/cSCC subclasses.

A Average histone modification profiles of undifferentiated and differentiated keratinocyte-specific peaks using previously published data generated on NHEK cells (ENCODE). The normalized signal of H3K27ac, H3K4me1/me2/me3, and H3K27me3 were measured in a window of ± 10,000 base pairs (bp).

B Fractions of CpGs located within epigenomic substructures for the 4,351 InfiniumEPIC CpG probes found within undifferentiated and differentiated keratinocyte-specific peaks.

C Unsupervised hierarchical clustering of 12 healthy, 20 AK, and 35 cSCC epidermal samples based on the methylation status at undifferentiated and differentiated keratinocyte-specific peaks. Each row represents the average methylation value of all CpGs contained in a particular peak.

D Principal Component Analysis (PCA) of 67 AK/cSCC and healthy controls performed with all detected CpGs after filtering (n = 632,778). Coloring is according to sample type and shape is according to cell-of-origin-related subclass.

Data information: AK: actinic keratosis, cSCC: cutaneous squamous cell carcinoma, DK: Differentiated keratinocytes, UK: undifferentiated keratinocytes.

human epidermal cells. Thus, we performed single-cell combinatorial indexing for methylation analysis (sci-MET) (Mulqueen *et al*, 2018) from a sun-protected healthy epidermis sample that was obtained from a 62 y/o male donor. In order to obtain a dataset with a higher sequencing coverage, we generated three sci-MET libraries containing only around 200 epidermal cells each. This resulted in the detection of 554 cells after read alignment and single-cell demultiplexing, with an average CpG coverage per cell of 0.85% (0.14–6.88%), in agreement with published data (Mulqueen *et al*, 2018). Single cells showed a detectable heterogeneity and subclustering as well as differences in their methylation content, suggesting methylation changes within the population (Appendix Fig S4). We then performed a multidimensional scaling (MDS) analysis with the 554 single-cell methylomes and the 55 AK/cSCC and 12 healthy epidermis EPIC samples. Of note, 548 single-cell methylomes grouped closely with the healthy epidermis and keratinocyte-like tumors, while six cells clustered with the EpSC-like tumors (Fig 3A). To assess whether these two cell clusters represented EpSC and differentiated keratinocytes, respectively, we examined their average methylation level at different genomic regions based on the NHEK ChromHMM segmentation (see Materials and Methods for details) (Ernst *et al*, 2011). Consistent with their keratinocyte identity, both cell clusters displayed low methylation levels in promoter regions that were designated as active in NHEKs and high methylation in regions that were designated as actively transcribed in NHEKs (Fig 3B). Also, repressed, heterochromatic, and repetitive regions displayed high methylation levels in both cell groups, as expected (Deplus *et al*, 2014) (Fig 3B). In agreement with the loss of DNA methylation in lineage-specific regulatory elements upon epidermal differentiation seen in mice (Bock *et al*, 2012) and humans (Sen *et al*, 2010), enhancer regions, and especially strong enhancers, were found to be less methylated in cells clustering with keratinocyte-like tumors (Fig 3B). These findings further support the notion that the two cell clusters represent EpSCs and differentiated keratinocytes, respectively. Importantly, the genomic regions of AK/cSCC showed very similar methylation patterns compared with the single-cell profiles, with keratinocyte-like tumors displaying substantial enhancer hypomethylation (Fig 3C). Taken together, these results provide important confirmation for the cell-of-origin interpretation of the methylation-based AK/cSCC subclasses.

© 2022 The Authors

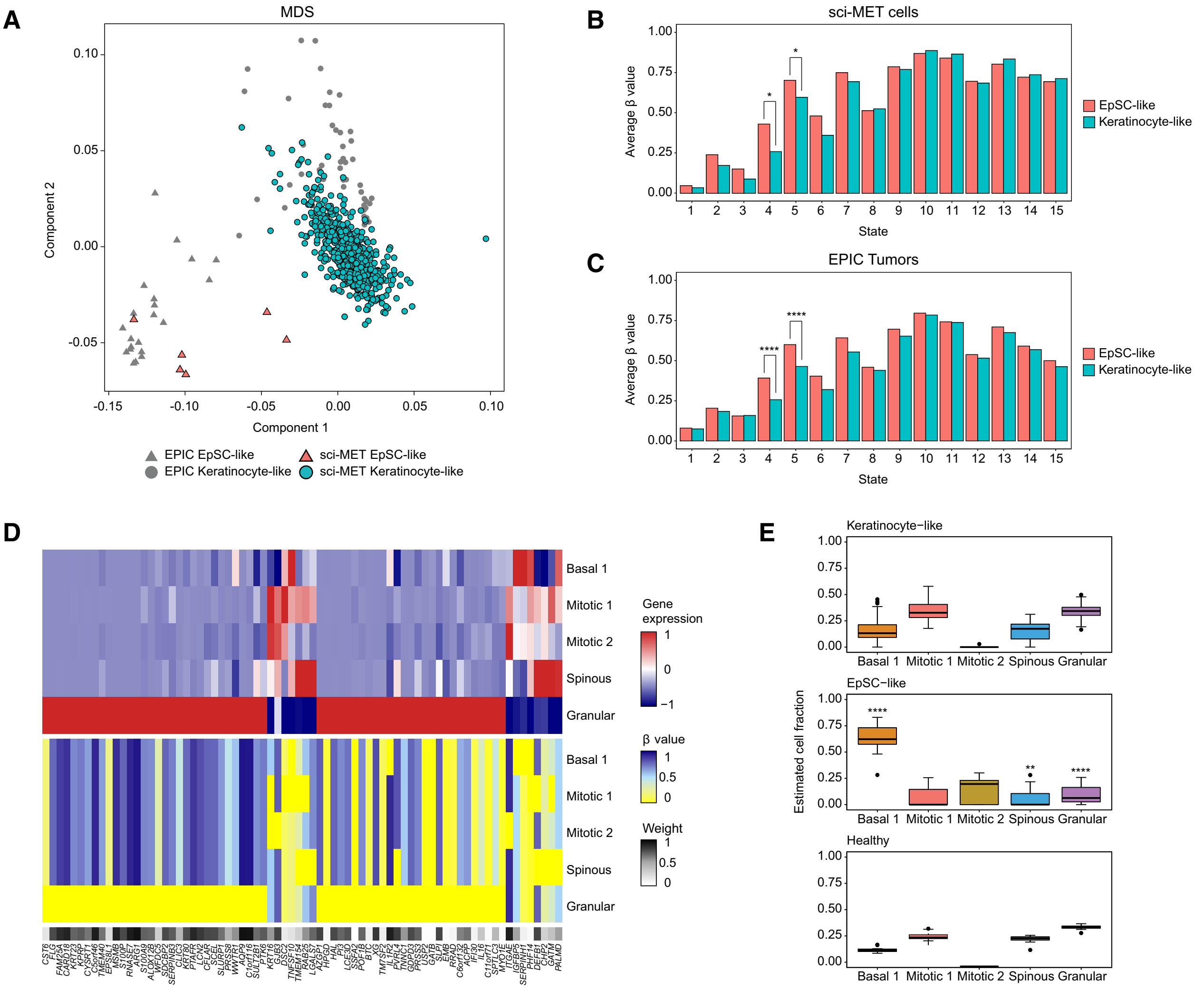

Figure 3. Single-cell methylomics validates cell-of-origin-based stratification for AK/cSCC.

A Multidimensional Scaling (MDS) analysis of the 67 bulk DNA methylomes from AK/cSCC and healthy samples, and the 554 single-cell methylomes obtained with sci-MET.

B, C Average DNA methylation in the 15 chromatin states defined by ChromHMM in NHEK for (B) both EpSC and Keratinocytes from the sci-MET single-cell dataset, or for (C) EpSC-like and keratinocyte-like AK/cSCC from the EPIC dataset. ChromHMM state 1: active promoter; state 2: weak promoter; state 3: inactive/poised promoter; state 4: strong enhancer; state 5: strong enhancer; state 6: weak/poised enhancer; state 7: weak/poised enhancer; state 8: insulator; state 9: transcriptional transition; state 10: transcriptional elongation; state 11: weak transcription; state 12: polycomb-repressed; state 13: heterochromatin/low signal; state 14: repetitive/copy number variation; state 15: repetitive/copy number variation.

D Heatmaps displaying the gene expression (upper) and the imputed promoter DNA methylation (lower) reference matrices for keratinocyte populations involved in terminal differentiation, calculated by EpiSCORE. The weight of each gene in the reference DNA matrix is also depicted.

E Boxplots displaying estimated cellular fractions of the keratinocyte populations involved in the differentiation trajectory in keratinocyte-like and EpSC-like AK/cSCC as well as in healthy epidermis.

Data information: in Boxplots, the central bar, boxes, and whiskers represent the median, first and third quartiles, and 1.5-time interquartile range (IQR), respectively. Statistical analyses in (B) and (C) were performed using a Wilcoxon Rank Sum test comparing the average methylation values in genomic states 4 and 5 between EpSC (n = 6) and keratinocytes (n = 548) (B) or EpSC-like (n = 22) and keratinocyte-like (n = 33) AK/cSCC samples (C). Statistical analysis in (E) was performed using a Wilcoxon Rank Sum test, comparing the Basal 1, Spinous and Granular fractions between cell-of-origin-related subclasses (EpSC-like: n = 22; keratinocyte-like: n = 33). *$P < 0.05$, **$P < 0.01$, ***$P < 0.001$, ****$P < 0.0001$.

While we only detected two epidermal differentiation stages in our single-cell methylation analysis, we observed a higher number using single-cell transcriptomics and chromatin accessibility analyses. To assess whether the DNA methylation patterns represent several transcriptomic states and to further explore the keratinocyte composition of AK/cSCC, we used computational deconvolution of cell-type fractions in the bulk DNA methylation datasets based on scRNA-seq data (Teschendorff *et al*, 2020). After characterization of the reference scRNA-seq dataset (Fig EV2), we generated the reference expression and DNA methylation matrices for bulk methylome deconvolution (Fig 3D; see Materials and Methods). Cell fraction estimation revealed an overall similar keratinocyte composition in

 © 2022 The Authors

healthy epidermis and keratinocyte-like tumors, with higher proportions of well-differentiated spinous and granular keratinocytes (Fig 3E). In contrast, EpSC-like AK/cSCC showed enrichment for the EpSC-containing Basal 1 population (Fig 3E). Collectively, these results indicate that the two methylation profiles reflect transcriptionally distinct epidermal differentiation stages. Furthermore, the methylation-based AK/cSCC subclasses display a differential enrichment for keratinocyte populations at the start and end of the lineage trajectory. These findings thus provide further confirmation for their EpSC-like and keratinocyte-like origin, respectively.

Methylation-based subclasses display distinct phenotypic features

To further characterize the two cell-of-origin-based subclasses identified in the bulk methylome analysis, we used mitotic clock algorithms (Teschendorff, 2020). We observed an increased mitotic age in both tumor types in comparison with healthy epidermis (Fig 4A). This increase was more pronounced in the keratinocyte-like subgroup, while the EpSC-like tumors showed a more moderate effect (Fig 4A). Subsequent calculations of the intrinsic stem cell division rate (SCDR) estimated an SCDR of 10.35 divisions per stem cell and year in the healthy epidermis (Fig 4B). This is similar to experimentally assessed division rates for human EpSC (Maeda, 2017). In agreement with the increased mitotic age, we also observed an increase in the SCDR in AK/cSCC, with a more pronounced effect in keratinocyte-like tumors (SCDR = 39.7; Fig 4B). On the contrary, the EpSC-like tumor subclass again showed a more moderate effect (SCDR = 25, Fig 4B). These results suggest different proliferation rates for the two cell-of-origin-based subclasses.

Cellular proliferation and invasion are known to be two independent processes with a high degree of anticorrelation in cancer cells (Gao *et al*, 2005; Hoek *et al*, 2008; Hecht *et al*, 2015). Thus, we hypothesized that a less proliferative phenotype in EpSC-like tumors could indicate a higher invasiveness in these cases. Following this line of thought, we investigated whether the EpSC-like tumors displayed epigenetic features of an invasive phenotype by analyzing the methylation status of three miRNAs known to be silenced by promoter hypermethylation in cancer cells displaying epithelial-to-mesenchymal transition (EMT) (Wiklund *et al*, 2011; Davalos *et al*, 2012). Indeed, promoter regions for the *MIR200C/141* cluster and *MIR205* were found to be highly methylated, specifically in the EpSC-like subclass (Fig 4C). Furthermore, immunofluorescence staining of ZEB2, an EMT-driving transcription factor repressed by the miR200 family and miR205 (Gregory *et al*, 2008; Park *et al*, 2008), showed an increase in ZEB2-positive nuclei in EpSC-like cSCC samples (Fig 4D). These results are consistent with distinct proliferative and invasive characteristics for the two AK/cSCC cell-of-origin-based subclasses.

Methylation-based subclasses display distinct clinical features

To assess whether the phenotypic differences observed between cell-of-origin-based subclasses also resulted in distinct clinical features, we analyzed the DNA methylation patterns of additional epidermal tumors with different metastatic potentials. Thus, we generated new EPIC datasets containing 11 *in situ* squamous cell carcinoma (Bowen's disease; BD), another type of pre-invasive lesion leading to invasive cSCC, 14 basal cell carcinoma (BCC), the most common KC in the general, immunocompetent population, and 10 non-cancerous senile warts (seborrheic keratosis, SK). These newly generated datasets were combined with our dataset containing healthy, AK and cSCC samples. Tumor stratification based on methylation patterns at the differentiated keratinocyte-specific accessible regions identified in our scATAC-seq uncovered the two previously described cell-of-origin-related subclasses, which could again be observed in a PCA performed with all CpG probes (Figs 5A and EV3A). Similar to AK and cSCC samples, BD lesions were also stratified into the two cell-of-origin-based subclasses (Fig 5A). Moreover, further analyses indicated almost indistinguishable methylomes between precursor AK lesions, *in situ* carcinomas, and cSCC arising from the same cell type, but highlighted major epigenetic differences between cell-of-origin-based subclasses (Fig EV4). In contrast, rarely metastatic BCC and non-cancerous SK cases were almost exclusively classified as keratinocyte-like (Figs 5A and EV3A). Our analysis thus stratifies all the main keratinocyte cancer entities according to two main initiating cells-of-origin and suggests a bias toward lower metastatic potential for the more differentiated subclass.

In addition, we investigated a published dataset comprising five sclerodermiform BCC (sBCC) tumors (Sand *et al*, 2019a; Data ref: Sand *et al*, 2019b). sBCC is considered particularly aggressive as it presents high recurrence rates as well as higher local invasiveness (Sand *et al*, 2019a; Conforti *et al*, 2021), which predicted an enrichment of EpSC-like methylation patterns. Indeed, methylation patterns at the accessible regions identified three out of five samples as EpSC-like (Figs 5B and EV3B). When these datasets were integrated with our BCC datasets, which also included three sBCCs, only four out of 19 samples were classified as EpSC-like, three of which were from the sBCC subtype (Appendix Fig S5). These results indicate an enrichment for aggressive sBCC cases in the EpSC-like BCC subclass.

Furthermore, we analyzed a published EPIC dataset consisting of 12 common warts (verruca vulgaris; VV), another type of benign epidermal tumor linked to human papillomavirus infection (Al-Eitan *et al*, 2020). In accessible regions, methylation patterns of VV samples indicated a more differentiated cell-of-origin that was highly similar to those present in healthy epidermis (Figs 5C and EV3C). VV methylation patterns also appeared similar to those in SK, thus indicating a differentiated keratinocyte-like cell-of-origin for both senile (SK) and common (VV) warts.

Lastly, we stratified another published dataset, consisting of five AK and a collection of 18 invasive cSCC samples, ranging from initially invasive to metastatic (Hervás-Marín *et al*, 2019a; Data ref: Hervás-Marín *et al*, 2019b). Strikingly, this dataset was classified completely as EpSC-like (Figs 5D and EV3D), further suggesting a more invasive phenotype in EpSC-like cSCCs. To further validate these observations, we profiled the methylome of eight cSCC metastases and three metastasizing primary cSCC. These samples were obtained as formalin-fixed paraffin-embedded (FFPE) sections, and tumor tissue was isolated by laser microdissection to ensure high sample purity (Appendix Fig S6). Methylation analysis of differentiated keratinocyte accessible regions classified all 11 samples as EpSC-like (Figs 5E and EV3E), thus again suggesting the higher invasiveness and metastatic potential of this subclass.

© 2022 The Authors

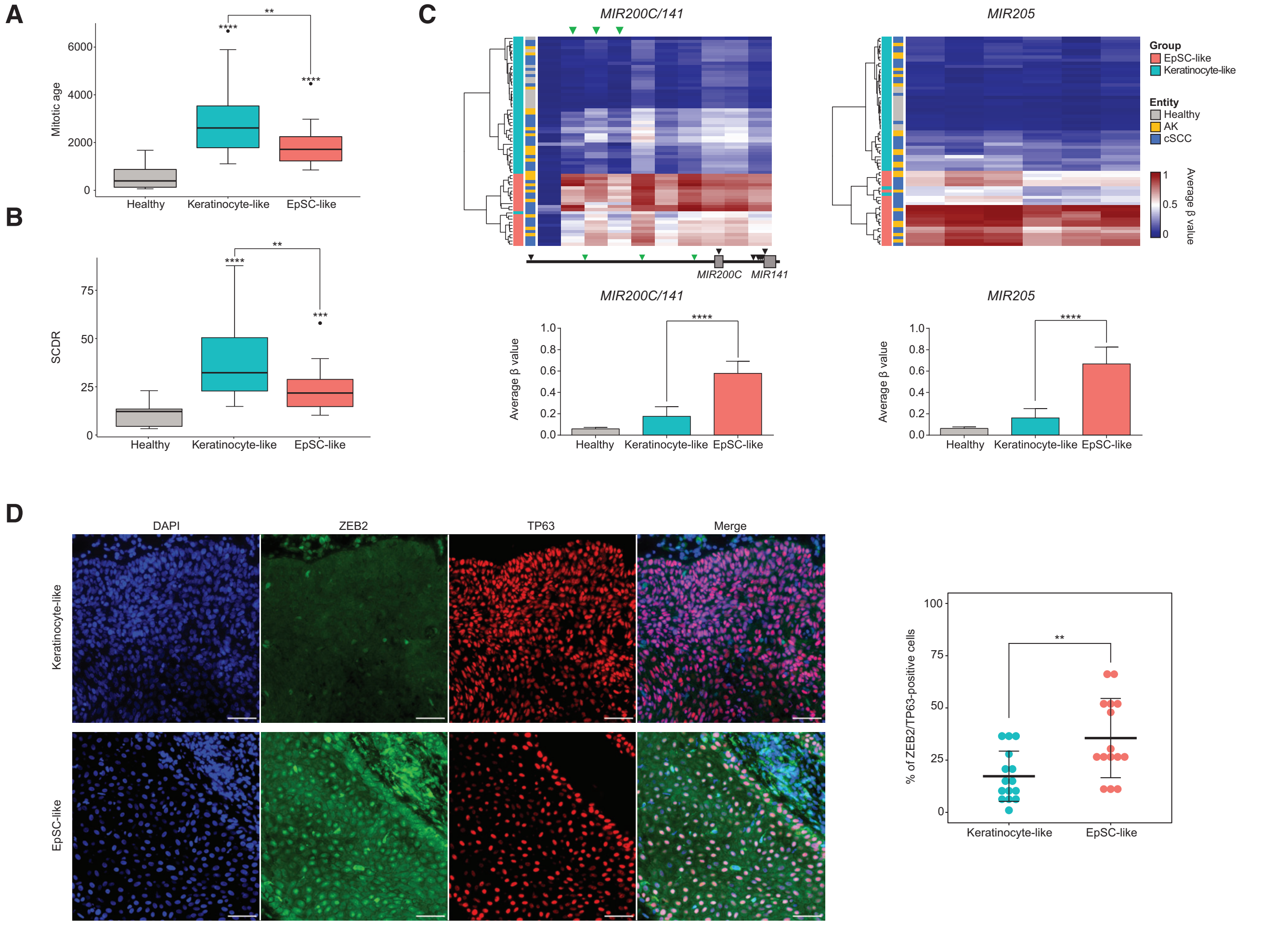

Figure 4. AK/cSCC cell-of-origin-based subclasses present distinct mitotic ages and EMT-related methylation features.

A Boxplots representing the total number of cumulative stem cell divisions per sample (mitotic age) as calculated with epiTOC2 in each AK/cSCC cell-of-origin-related subclass.

B Boxplots representing the stem cell division rate per stem cell and year in each entity and cell-of-origin-based subclass.

C Upper: Heatmaps displaying the unsupervised clustering of 67 AK/cSCC and healthy controls based on the methylation patterns of the *MIR200C/141* cluster and the *MIR205* gene. Lower: Bar plots showing the quantification of the methylation values at CpGs located at promoter regions in each cell-of-origin-based subclass. For the *MIR200C/141* cluster, probes located at the regulatory CpG island are depicted with green arrows. For the *MIR205* gene, all probes shown in the heatmap are part of the promoter region and were used for quantification.

D Left: Representative images of EpSC-like ($n = 5$) and Keratinocyte-like ($n = 5$) cSCC tumors stained for the EMT-marker ZEB2 (green) and the keratinocyte marker TP63 (red). Nuclei were counterstained with DAPI. Images are shown at 40x original magnification. Scale bar, 50 μm. Right: Quantification of ZEB2-positive tumor cells (TP63-positive) in three independent regions per sample. Each dot represents a region with at least 500 tumor cells (TP63-positive) counted.

Data information: In Boxplots in (A and B), the central bar, boxes and whiskers represent the median, first and third quartiles, and 1.5-time interquartile range (IQR), respectively. Barplots in (C) represent the mean and error bars represent the standard error of the mean (SEM). Statistical analyses in (A–C) were performed using a Wilcoxon Rank Sum test, comparing each subclass (EpSC-like: $n = 22$; keratinocyte-like: $n = 33$) to healthy samples ($n = 12$), or between cell-of-origin-related subclasses (depicted by a line). Statistical analysis in (D) was performed using an unpaired two-sided *t*-test (EpSC-like: $n = 5$ samples, 15 technical replicates; keratinocyte-like: $n = 5$ samples, 15 technical replicates). Central bar represents the mean and error bars represent the standard deviation. *$P < 0.05$, **$P < 0.01$, ***$P < 0.001$, ****$P < 0.0001$. AK: actinic keratosis, cSCC: cutaneous squamous cell carcinoma.

Discussion

The roles of chromatin accessibility and DNA methylation in establishing cell identity throughout lineage differentiation are well accepted (Guo *et al*, 2016; Greenberg & Bourc'his, 2019; Li *et al*, 2021). Moreover, cancer methylomes reflect the epigenetic programs of the tumor-initiating cell, which can be used to define distinct cell-of-origin-based tumor subclasses, often with clinical implications (Kulis *et al*, 2013; Moran *et al*, 2016; Hoadley *et al*, 2018). Our results show that DNA methylation patterns found at differentially accessible regions between undifferentiated and differentiated keratinocytes define human KC subtypes through their cells-of-origin. These results confirm previous observations for AK and cSCC (Rodríguez-Paredes *et al*, 2018a) with an analytical framework that is completely based on single-cell data from human epidermis samples. It is important to notice that the set of differentially

 © 2022 The Authors

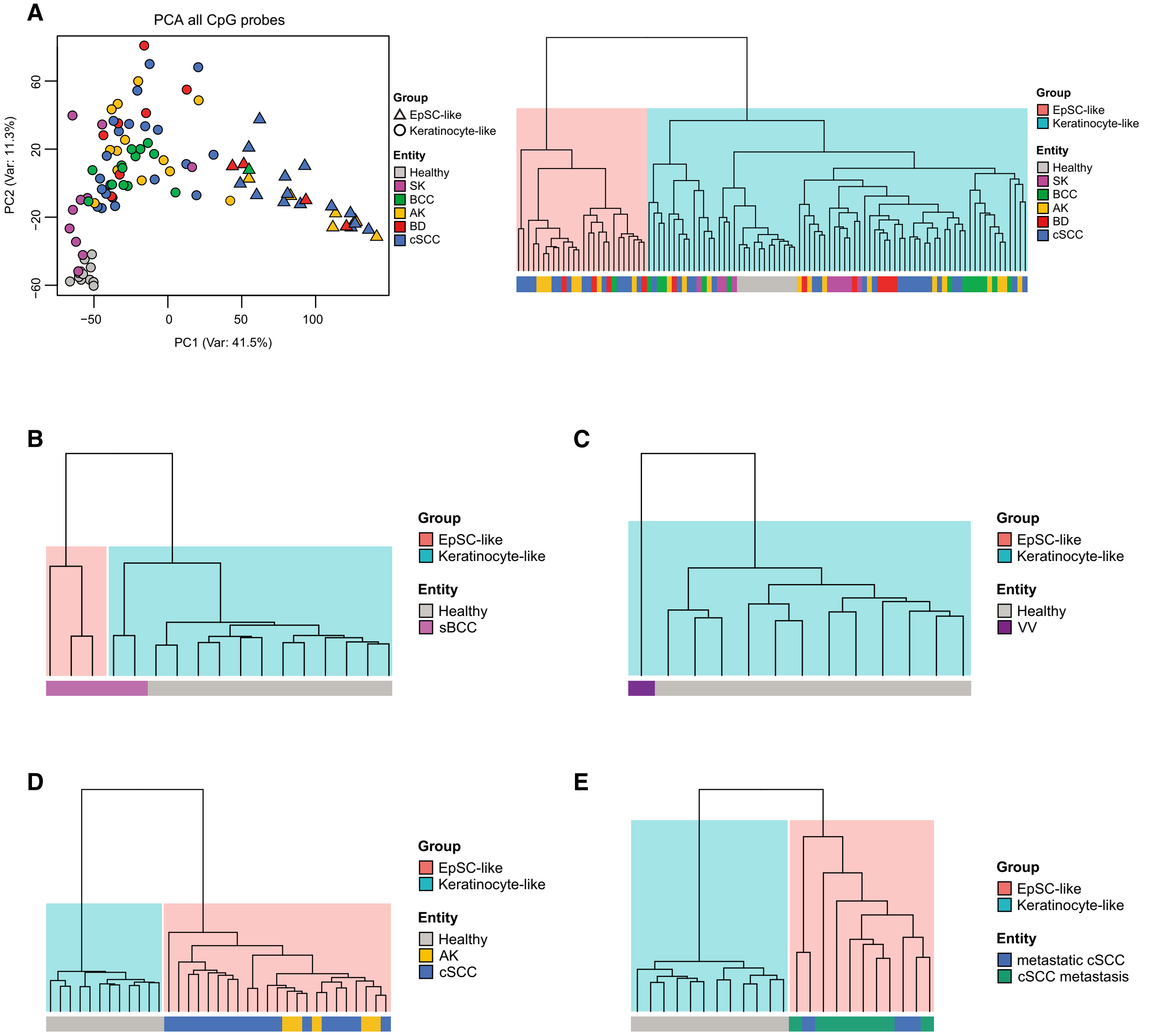

Figure 5. Epidermal tumors with lower metastatic potential arise from more differentiated cells-of-origin.

A Left: Principal Component Analysis of 12 healthy, 10 SK, 14 BCC, 20 AK, 11 BD, and 35 cSCC epidermal samples using all CpGs after filtering (n = 632,778). Coloring is according to sample type and shape is according to cell-of-origin-related subclass. Right: Unsupervised hierarchical clustering of 102 epidermal tumors and healthy controls based on the methylation status at differentiated keratinocyte-specific peaks.

B–E Unsupervised hierarchical clustering based on the methylation status at differentiated keratinocyte-specific peaks of (B) Five sBCC samples from Sand *et al* (2019a), Data ref: Sand *et al* (2019b); (C) 12 averaged VV samples from AL Eitan *et al.*(Al-Eitan *et al*, 2020); (D) Five AK and 18 cSCC samples from Hervás-Marín *et al* (2019a), Data ref: Hervás-Marín *et al* (2019b); (E) Eight cSCC metastases and three primary metastatic cSCC, together with 12 healthy samples from our cohort.

Data information: AK: actinic keratosis, BCC: basal cell carcinoma, BD: Bowen's disease, cSCC: cutaneous squamous cell carcinoma, sBCC: sclerodermiform basal cell carcinoma, SK: seborrheic keratosis, VV: verruca vulgaris.

accessible peaks identified in our analysis shows little overlap with the set of enhancers defined in an *in vitro* system of epidermal stem cell differentiation (Rinaldi *et al*, 2016) (Appendix Fig S7), which further highlights the importance of our *in vivo* approach.

Paired multimodal single-cell profiling provides new opportunities to study differentiation processes and for characterizing important cell states. Furthermore, combining various genomic read-outs is important for the identification of key transcription factors involved in differentiation trajectories. Our single-cell multi-omics analysis of the healthy human epidermis identified several well-known transcription factors associated with undifferentiated keratinocytes (i.e., TP63, TEAD1, and IRF1) (Truong *et al*, 2006; Yuan *et al*, 2020) and with terminally differentiated keratinocytes (i.e., CEBPA, GRHL1, and SREBF2) (Maytin & Habener, 1998; Gulati

© 2022 The Authors

et al, 2013; Mlacki et al, 2014). Of note, this approach also identified MEF2A as one of the key regulators for undifferentiated basal keratinocytes. MEF2A has been previously reported to exert important roles in the differentiation of several cell lineages such as the skeletal muscle and neuronal systems (Estrella et al, 2015; Zhu et al, 2018). However, it has not been linked to epidermal differentiation yet.

Single-cell DNA methylation approaches hold great potential to further explore the role of this epigenetic modification in the context of cellular differentiation. However, their development is still in early stages, with limited applicability (Karemaker & Vermeulen, 2018). Combinatorial indexing (Adey et al, 2014; Amini et al, 2014) could provide a solution to many current limitations due to its high scalability. However, the published protocol for single-cell combinatorial indexing to whole-genome bisulfite sequencing (Mulqueen et al, 2018) contains important inaccuracies and methodological gaps that have so far precluded a wider application of the method. After successfully addressing these shortcomings, we generated a sci-MET library containing 554 single cells with an average CpG coverage of 0.85%. Single-cell methylomes of human epidermal cells showed differences in average methylation content as well as a certain degree of dissimilarity, thus suggesting dynamic methylation changes upon differentiation *in vivo*. Single-cell DNA methylation studies are also critically important for identifying the exact cell type that provides the tumor cell-of-origin. Thus, we combined our extensive bulk AK/cSCC methylation dataset with the 554 single-cell methylomes, which identified six out of the 554 cells as EpSCs and clustered them with the EpSC-like tumors. Human EpSCs have been previously estimated to represent approximately 1% of the epidermal cells (Rachidi et al, 2007). Our results are consistent with these numbers and validate the cell-of-origin-based stratification of human AK/cSCC.

We also used scRNA-seq data from more than 32,000 epidermal cells to define the main keratinocyte populations along the epidermal differentiation trajectory. We identified a cell composition that was similar to previous reports (Ji et al, 2020; Cheng et al, 2018a). However, contrary to the multiple branches identified in another scRNA-seq analysis using a graph-based approach (Cheng et al, 2018a), our RNA velocity analysis suggests a single differentiation trajectory. Interestingly, our results suggest that only one of the two basal populations found in adult human epidermis (Basal 1) is located at the beginning of the differentiation trajectory, followed by the mitotic and more differentiated keratinocyte populations. *KRT19*-expressing Basal 2 cells were not part of the trajectory, which is consistent with previous reports (Michel et al, 1996; Pontiggia et al, 2009). Such keratinocytes are known to be self-renewing but not involved in terminal differentiation and might represent a stem cell reservoir for the interfollicular epidermis (Pontiggia et al, 2009). Deconvolution of bulk AK/cSCC methylation patterns using these scRNA-seq data detected distinct keratinocyte populations in both keratinocyte-like and EpSC-like DNA methylation subclasses. These results indicate that the methylation profiles defined by single-cell methylomics do not represent completely homogeneous keratinocyte populations. Furthermore, tumor heterogeneity is also consistent with published scRNA-seq results of human cSCC that revealed the presence of undifferentiated, mitotic, and well-differentiated keratinocyte populations, even when they share a common initiating cell (Ji et al, 2020). Importantly, our approach also indicated a strong enrichment for Basal 1 keratinocytes in EpSC-like tumors, while finding differentiated spinous and granular cells in the keratinocyte-like cases.

DNA methylation clocks are compound biomarkers that are increasingly used in cancer research (Yang et al, 2016; Duran-Ferrer et al, 2020; Teschendorff, 2020). When we used DNA methylation clocks to calculate the mitotic age and stem cell division rates (SCDR) in epidermal tumors, our results revealed a general increase in mitotic age and SCDR in AK/cSCC in comparison with the healthy epidermis, as described for several other malignancies (Yang et al, 2016; Teschendorff, 2020). While we observed a substantial increase in the keratinocyte-like subgroup, EpSC-like tumors showed a more moderate increase, which we interpreted to reflect a less proliferative but more invasive phenotype. This was confirmed by methylation analysis of miRNAs from the miR-200 family (i.e., miR-200-c and miR-141) and miR-205, which play an essential role in maintaining epithelial phenotypes by targeting the E-cadherin transcriptional repressors ZEB1 and ZEB2 (Gregory et al, 2008; Park et al, 2008). Silencing of these miRNAs by promoter hypermethylation has been described in several human cancer cell lines displaying EMT features (Neves et al, 2010; Davalos et al, 2012) and invasive epithelial human malignancies, such as muscle-invasive bladder cancer (MIBC) (Wiklund et al, 2011). Consistent with a more invasive phenotype, we observed promoter hypermethylation in *MIR200C/141* and *MIR205* genes and increased ZEB2-positive nuclei in EpSC-like tumors. Hence, our analysis proposes cell-of-origin-dependent differences in the invasive phenotype of AK/cSCC and thus establishes novel opportunities for the development of risk stratification biomarkers.

Following this line of thought, we expanded our DNA methylation-based stratification to other epidermal tumor entities with different metastatic potential, including *in situ* carcinoma (BD), rarely metastatic BCC, and non-cancerous senile and common warts. All entities could be stratified again into two subclasses displaying either EpSC-like or keratinocyte-like methylation profiles, indicating that the previously described bimodal cell-of-origin model can be applied to a wide range of epidermal tumors. Importantly, our analysis uncovered a prominent bias toward a more differentiated cell-of-origin for entities bearing a lower metastatic potential such as BCC or non-cancerous warts. In contrast, most EpSC-like tumors belonged to entities with a higher metastatic potential (cSCC), including precursor lesions (AK and BD) that can progress to metastatic cSCC if left untreated. The consistent classification of invasive cSCC samples, cSCC metastases and primary metastasizing cSCC samples entirely as EpSC-like tumors further supported this notion.

Altogether, our study thus provides novel insight into the role of chromatin accessibility and DNA methylation in epidermal differentiation and KC initiation and proposes a general stratification strategy for epidermal tumors that might improve patient risk assessment.

Materials and Methods

Samples

For scRNA-seq, single-cell multi-omics and sci-MET experiments, we obtained remnant clinically healthy whole skin from patients

 © 2022 The Authors

undergoing routine surgery at Heidelberg University Hospital. Samples were obtained from the sun-protected ilioinguinal region after written informed consent by the patients, in compliance with current legislation and as approved by the Ethics Committee of Heidelberg University (no. S-091/2011). All samples used for these experiments were obtained from male donors.

Punch biopsies (4-mm) from four AK, 17 cSCC, 11 BD, 14 BCC, and 10 SK samples (Table EV1) were obtained at the Department of Dermatology of the Heidelberg University Hospital, as approved by the ethics committee of Heidelberg University (protocol no. S-091/2011). Moreover, 12 healthy, 16 AK, and 18 cSCC samples previously analyzed and used for publication were also included in this study (Rodríguez-Paredes *et al*, 2018a; Data ref: Rodríguez-Paredes *et al*, 2018b). All samples were immediately immersed in liquid nitrogen after resection and stored at −80°C. Epidermal regions of the tumors were separated from the dermis by heat-split (incubated in pre-warmed PBS at 37°C for 1 min and then at 56°C for up to 5 min) and carefully dissected manually under a magnifying glass. Only tumor samples for which proper epidermis isolation was achieved were included in the study. DNA was isolated using the QIAamp DNA Investigator Kit (Qiagen) following the manufacturer's instructions.

Metastatic primary cSCC and cSCC metastasis samples (Table EV1) were obtained as 7-μm FFPE sections provided by the tissue bank of the National Center for Tumor Diseases (NCT Heidelberg, Germany) and the Department of Dermatology of Heidelberg University Hospital, in accordance with the regulations of the tissue bank and the approval of the ethics committee of Heidelberg University (protocol no. S-091/2011). Sections were placed on MembraneSlide NF 1.0 PEN (Zeiss) slides, and tumor tissue was isolated by laser microdissection using the Zeiss PALM MicroBeam system (Zeiss). DNA was subsequently isolated using the QIAamp DNA Micro Kit (Qiagen) following the manufacturer's instructions.

Diagnosis and histopathological features of both fresh-frozen (FF) and FFPE tumor samples obtained at Heidelberg University Hospital were routinely established by an expert dermatohistopathologist and reviewed before inclusion in this study.

Handling of samples and data was performed in a pseudonymized manner, also in strict compliance with the current legislation and institutional guidelines for data protection and privacy of the participating patients.

Single-cell multi-omics sequencing

Healthy whole skin biopsies were obtained from two male donors of fair-skin type (55 and 72 y/o) and preserved in MACS Tissue Storage Solution (Miltenyi Biotec). Samples were subsequently cut into small pieces that were further processed using the Epidermis Dissociation Kit, human (Miltenyi Biotec) and the Gentle MACS Dissociator (Miltenyi Biotec), following the manufacturer's instructions. Nuclei were isolated from the resulting single-cell suspension using the lysis buffer recipe described in (Wysocka *et al*, 2001) In brief, epidermal cells were resuspended in Buffer A (10 mM HEPES pH 7.9, 10 mM KCl, 1.5 mM $MgCl_2$, 0.34 M sucrose, 10% glycerol, 1 mM DTT, and 1X protease inhibitor cocktail) containing freshly added Triton X-100 at a final concentration of 0.1%. Cells were resuspended in a 1×10^6 cells/25 μl of Buffer A ratio and were incubated for 10 min on ice. Nuclei were recovered by centrifugation at 1,300 *g* for 5 min at 4°C and resuspended in 1× Nuclei Buffer (10× Genomics).

scATAC-seq and scRNA-seq libraries were generated using the Chromium Next GEM Single Cell Multiome ATAC + Gene Expression Reagent Kit (10× Genomics), as described by the manufacturer. Approximately, 10,000 nuclei per sample were loaded into a Chromium Single Cell Controller (10× Genomics) as initial input. Quantification of the library was carried out using the Qubit dsDNA HS Assay Kit (Life Technologies), and cDNA integrity was assessed using D1000 ScreenTapes (Agilent Technologies). Paired-end (28 + 90 bp) sequencing (100 cycles) was used for the scRNA-seq libraries while paired-end (50 + 50 bp) sequencing (100 cycles) was used for the scATAC-seq libraries, both performed with a NovaSeq 6000 device (Illumina).

Single-cell multi-omics sequencing data analysis

Raw sequencing data were processed with the Cell Ranger software (version 2.1.0) from 10× Genomics, and downstream analysis was performed using the Seurat (version 4.0.5) (Stuart *et al*, 2019) and Signac (version 1.5.0) (Stuart *et al*, 2021) packages. Low-quality cells were filtered out using Signac by removing those with less than 1,000 or more than 50,000 UMIs in the scRNA-seq and those with less than 1,000 or more than 100,000 counts in the scATAC-seq data. Furthermore, we also filtered out cells with a higher nucleosome signal than 2 and a transcriptional start site (TSS) enrichment lower than 1. The final datasets thus contained 2,851 and 2,714 single cells. We then used MACS2 to call the ATAC peaks on each sample independently. To ensure comparability between samples, we created a common set of peaks by merging all intersecting peaks using the GenomicRanges package (version 1.46.1) (Lawrence *et al*, 2013).

To avoid batch effects, we integrated the scRNA-seq datasets from the two samples using the standard protocol described in the Seurat package (Stuart *et al*, 2019) and as described above. We used default parameters and 30 CCA dimensions for the integration. Data dimensionality was reduced, and cell embeddings were calculated for both scRNA-seq and scATAC-seq data for the integrated dataset using PCA and latent semantic indexing (LSI), respectively. Then, the cell embeddings were integrated using the IntegrateEmbeddings() function from Signac using the anchors identified for the data integration of the scRNA-seq data. Lastly, we generated a joint dimensional reduction combining both scATAC-seq and scRNA-seq data using the weighted nearest neighbor method from Seurat and using 50 dimensions for each assay.

Unsupervised clustering of the integrated data was performed on the scRNA-seq data using 50 PCA dimensions and 0.4 resolution, which resulted in 10 cell clusters that were visualized by UMAP. Cell-type identity was established by transferring the cell labels from the reference scRNA-seq dataset containing more than 30,000 epidermal cells. To that end, we used 30 PCA dimensions for identifying the transfer anchors and for transferring the cell-type labels to the multiome dataset. Differentially accessible regions between undifferentiated (cluster 2) and differentiated keratinocytes (cluster 3) were identified using the FindMarkers() function. To assess the histone modification landscape at differentially accessible regions, we made use of previously published

© 2022 The Authors

ChIP-seq data of histone modifications generated on NHEK cells (ENCODE) and available at the UCSC genome annotation database. The analysis was performed using the ChipPeakAnno (v.3.28.1) package (Zhu *et al*, 2010).

Cis-regulatory interactions at the epidermal differentiation complex (EDC) were predicted by identifying co-accessible peaks in undifferentiated (cluster 2) and differentiated keratinocytes (cluster 3) independently using Cicero (v.1.3.6) (Pliner *et al*, 2018). Only Cicero connections with a co-accessibility score higher than 0.25 were plotted.

Overrepresented TF-binding motifs in differentially accessible peaks were identified using the FindMotifs() function with default parameters. In addition, motif activity was also calculated in each individual cell using chromVAR (Schep *et al*, 2017). Then, cell-type-specific gene markers and active motifs were identified using a Wilcoxon rank-sum test and the area under the receiver operator curve (auROC) with the presto package (v.1.0.0). Cell-type-specific transcription factors were obtained by ranking transcription factors by the average AUC statistic from both gene expression and motif activity for each cluster.

Regulatory gene network inference was performed using pySCENIC (v0.11.2) (Kumar *et al*, 2021) with default parameters in Phython (v3.7), following the previously described protocol (Aibar *et al*, 2017; Kumar *et al*, 2021). In brief, potential regulatory interactions were inferred based on the expression of predefined human transcription factors and their target genes in the preprocessed gene expression data from keratinocyte clusters defined by the single-cell multi-omics approach, using the GRNBoost2 algorithm. These interactions were then used to calculate TF-gene co-expression modules, which were subsequently subjected to motif enrichment analyses. Thus, only target genes that contained the corresponding TF-binding site were kept in the module. The activity of the resulting regulons was then assessed in individual cells using the AUCell score method. Lastly, to identify cell-type-specific regulons, we calculated a *Z*-score as previously described, and the top five regulons per cell type were displayed as a heatmap.

Single-cell RNA sequencing

A healthy whole skin biopsy was obtained from a 30 y/o male donor of fair-skin type and preserved in MACS Tissue Storage Solution (Miltenyi Biotec). The skin sample was subsequently cut into small pieces that were further processed using the Epidermis Dissociation Kit, human (Miltenyi Biotec) and the Gentle MACS Dissociator (Miltenyi Biotec), following the manufacturer's instructions. The resulting cell suspension was then filtered through a 70-µm cell strainer (Falcon) and depleted of apoptotic and dead cells with the Dead Cell Removal Kit (Miltenyi Biotec).

We used the 10× Genomics platform to generate a sequencing library with the Chromium Single Cell 3' Reagent Kit, v2 chemistry (10× Genomics), as described by the manufacturer. Approximately, 20,000 cells were loaded into a Chromium Single Cell Controller (10× Genomics) as initial input. Quantification of the library was carried out using the Qubit dsDNA HS Assay Kit (Life Technologies), and cDNA integrity was assessed using D1000 ScreenTapes (Agilent Technologies). Paired-end (26 + 74 bp) sequencing (100 cycles) was finally performed with a HiSeq 4000 device (Illumina).

Single-cell RNA sequencing data analysis

Raw sequencing data were processed with the Cell Ranger software (version 2.1.0) from 10× Genomics, and downstream analysis was performed using the Seurat package (version 3.1.1) (Stuart *et al*, 2019). A total of 7,752 cells passed the quality control of Cell Ranger. Further filtering of low-quality cells was carried out using Seurat by removing those expressing < 200 genes or more than 2,500, as well as cells expressing more than 5% of mitochondrial genes. The final dataset thus contained 7,143 single-cell transcriptomes. Unsupervised cell clustering was performed using 20 PCA dimensions and 0.5 resolution, which resulted in 10 clusters, and visualized as uniform manifold approximation and projection (UMAP) plots. Each cluster's representative gene markers were identified using the FindAllMarkers() function.

To combine our scRNA-seq sample with the three previously published abdominal epidermis samples (Cheng *et al*, 2018a; Data ref: Cheng *et al*, 2018b), we performed sample integration following Seurat's standard protocol (Stuart *et al*, 2019). First, gene expression in each cell was normalized using a log-normalization of the Unique Molecular Identifier (UMI) counts for each sample independently. Also, the 2,000 most variable genes per sample were identified. These features were then used to find common anchors using FindIntegrationAnchors(), with default parameters and 30 canonical correlation analysis (CCA) dimensions. Final integration was subsequently achieved using these anchors in IntegrateData(), with 30 CCA dimensions and default parameters.

Unsupervised clustering of the integrated data was performed using 30 PCA dimensions and 0.4 resolution, which resulted in 13 cell clusters that were visualized by UMAP projection. Cell-type identity was established comparing the most representative genes found by FindAllMarkers() and literature-based gene markers.

RNA velocity analysis

Spliced and unspliced reads from the in-house scRNA-seq dataset were obtained by running the command line interface of velocyto (version 0.17) (la Manno *et al*, 2018). For this analysis, we only used the in-house dataset, as the not preprocessed raw data were not available for the rest of the samples. Data were then preprocessed by normalization, log-transformation, and identification of highly variable genes, before calculating the RNA velocity. Additionally, melanocytes and immune cells were removed from the dataset, resulting in 7,068 keratinocytes that were used for further analysis. RNA velocity was then estimated by the generalized dynamical model of scVelo (version 0.2.3) (Bergen *et al*, 2020), using the recover_dynamics() function with 100 maximum iterations. Lastly, RNA velocities were projected and visualized onto the UMAP embedding calculated by Seurat. The latent time was calculated using the default values and manually assigning the root cells to cluster Basal 1, based on the RNA velocity results.

DNA methylation analysis

DNA methylation data from epidermal tumors were obtained using Infinium MethylationEPIC BeadChips (Illumina), according to the manufacturer's protocols, at the Genomics and Proteomics Core Facility of the German Cancer Research Center (DKFZ, Heidelberg,

 © 2022 The Authors

Germany). For FFPE samples, a special restoration protocol was applied during sample preparation to ensure good sequencing quality, following the manufacturer's instructions.

Data processing and analysis were performed using the R Bioconductor package minfi (version 1.34.0) (Aryee *et al*, 2014), as previously described (Rodríguez-Paredes *et al*, 2018a). Briefly, raw sequencing data were preprocessed by filtering out probes located in sex chromosomes as well as low-detected, self-hybridizing, and SNP-associated CpGs. Data normalization was performed using the function preprocessFunnorm(). Methylation levels for each individual CpG were displayed as β values, which are calculated using the ratio of methylated and unmethylated intensities per locus (β = methylated/(methylated + unmethylated + 100)). Detection of differentially methylated probes (DMPs) was carried out by fitting a linear model and using an empirical Bayes method for statistical testing. Multiple testing was corrected using the Benjamini-Hochberg method, and DMPs were filtered by significance threshold (*P*-value < 0.05, *F*-test).

Gene Ontology analysis using differentially methylated probes was performed using the R package methylGSA (version 1.6.1) (Ren & Kuan, 2019). As promoters, we used CpG probes corresponding to the TSS1500, TSS200, 1stExon, and 5′UTR locations obtained from the UCSC reference group of the Illumina annotation, contained in the IlluminaHumanMethylationEPICanno.ilm10b4.hg19 R package (Hansen, 2017).

Mitotic age and stem cell division rates (SCDR) were calculated using the publicly available epiTOC2 R Script with default parameters (Teschendorff, 2020).

DNA methylation-based stratification of KC samples

CpG probes located at the set of differentially accessible regions between undifferentiated and differentiated keratinocytes identified by scATAC-seq and contained in the Infinium MethylationEPIC were identified (*n* = 4,351). The number of CpG probes detected in peaks from undifferentiated keratinocytes was significantly higher (2,925 probes) than in peaks from differentiated keratinocytes (1,426 probes). This was most likely due to the fact that peaks in undifferentiated keratinocytes cover higher DNA sequences in general than those in differentiated keratinocytes. Furthermore, peaks in differentiated keratinocytes are enriched for enhancers, usually associated with intergenic regions, which are underrepresented in EPIC arrays.

Then, methylation values for CpG probes located in the same region were averaged for each sample. Tumor stratification was performed by hierarchical clustering using complete-linkage and Euclidean distances. Clustering was then visualized as heatmaps or dendrograms.

We used healthy epidermis methylation as a control as it mostly represents the methylome of terminally differentiated keratinocytes. Thus, we combined each previously published dataset with the 12 healthy epidermis samples from our cohort. Raw methylation data were not available for the dataset published in AL Eitan *et al.*, so we used processed mean β values per group as provided in the original publication (Al-Eitan *et al*, 2020). Stratification of these datasets was performed as described above.

Tn5 activity assessment and transposome assembly

For the sci-MET experiment, a homemade Tn5 transposase provided by the Protein Expression and Purification Core Facility at the *European Molecular Biology Laboratory* (EMBL; Heidelberg, Germany) was used, and its activity was assessed as follows. First, forward linker oligonucleotides (FC121-1030 and FC121-1031) and the reverse linker Tn5MERev, obtained from (Picelli *et al*, 2014), were resuspended at 100 μM in EB Buffer (Qiagen). Each forward linker was then combined with the reverse oligo at a 1:1 ratio and subsequently annealed in a thermocycler (95°C for 5 min, cool-down to 65°C, 65°C for 5 min, cool-down to 4°C). Cool-down steps were performed at a −0.1°C/s rate. Next, transposomes were assembled by adding 0.5 μl of each annealed linker to 10 μl of the Tn5 stock, followed by an incubation at 23°C for 1 h in a thermocycler.

After assembly, transposomes were diluted in 50% glycerol at different concentrations to assess the proper working concentration. Tagmentation was performed in a 5 μl reaction containing 2.5 μl of 2× Tagmentation Buffer (20 mM Tris–HCl pH 7.5, 20 mM $MgCl_2$, 50% DMF), 1.25 μl of the diluted Tn5, and 150 pg of cDNA as input. Samples were incubated at 55°C for 3 min and subsequently cooled down to 10°C. Then, reactions were stopped by adding 1.25 μl of 0.2% SDS followed by a 5 min incubation at room temperature. Tagmented cDNA amplification was directly carried out by adding 10 μl of the following PCR mixture: 6.75 μl of KAPA HiFi HotStart ReadyMix (Roche), 0.75 μl of DMSO, 1.25 μl of 10 μM Illumina i7 adapter (5′-CAAGCAGAAGACGGCATACGAGATGTCTCGTGGGCTCGG), and 1.25 μl of 10 μM Illumina i5 adapter (5′- AATGATACGGCGACCACCGAGATCTACACTCGTCGGCAGCGTC). The amplification reaction was performed by an incubation for 3 min at 72°C and 30 s at 95°C, followed by 12 cycles of 20 s at 98°C, 15 s at 58°C and 30 s at 72°C, ending with a final incubation of 30 s at 72°C.

Amplified DNA was then cleaned-up using AMPureXP Beads (Beckman Coulter) at 1× volume. Samples were incubated for 5 min at room temperature and then placed in a magnetic rack where beads were washed in 80% EtOH. Lastly, DNA was eluted in 10 μl of H_2O. The Tn5 activity was assessed by checking DNA quantification and fragment size distribution using the Qubit dsDNA HS Assay Kit (Life Technologies) and D5000 ScreenTapes (Agilent Technologies), respectively. The 1:50 dilution was found to provide the highest yield with an adequate fragment size distribution for subsequent sequencing, so it was finally used for the sci-MET experiment.

To assemble the sci-MET transposomes, the 96 unique Cytosine-depleted linkers and the reverse complement primer described in Mulqueen *et al* (2018) were resuspended at 100 μM in EB buffer (Qiagen) and combined at a 1:1 ratio. Linkers were then annealed as described above and diluted at 1:50 in EB Buffer (Qiagen). The Tn5 stock transposase was also diluted at 1:50 in 50% glycerol. Then, transposomes were assembled by adding one volume of the diluted linkers to 10 volumes of the diluted transposase (i.e., 1 μl of diluted linker to 10 μl of diluted Tn5), followed by an incubation for 1 h at 23°C and room temperature. Assembled transposomes were stored at −20°C.

Single-cell combinatorial indexing for methylation analysis (sci-MET)

A healthy whole human skin biopsy was obtained from the ilioinguinal region of a 62 y/o male donor of fair-skin type. The sample

© 2022 The Authors

was immersed in MACS Tissue Storage solution (Miltenyi Biotec) immediately after resection and was kept on ice until further processing. The single-cell suspension from the epidermis was obtained using the Epidermis Dissociation Kit, human (Miltenyi Biotec), following the manufacturer's instructions. We then followed the previously published sci-MET protocol (Mulqueen *et al*, 2018), with important modifications. Hence, cells were then fixed by incubation with 1.5% formaldehyde (without methanol) in 1 ml of PBS for 10 min, with gentle shaking. The reaction was stopped by adding 80 µl of 2.5 M glycine followed by 5 min incubation on ice. The sample was centrifuged at 550 *g* for 10 min at 4°C to recover fixed cells.

Nuclear isolation was performed using the lysis buffer recipe described in (Wysocka *et al*, 2001) and as described for the single-cell multi-omics dataset. Recovered nuclei were subsequently subjected to nucleosome depletion. To do so, fixed nuclei were resuspended in 800 µl of 1× NEBuffer 2.1 (New England Biolabs) supplemented with 0.3% SDS, and incubated for 30 min at 42°C with intense shaking. Then, 200 µl of 10% Triton X-100 was added to the sample, which was incubated for 30 min at 42°C with intense shaking. Nuclei were then centrifuged at 500 *g* for 5 min at 4°C and filtered through a 40-µm cell strainer. The nuclear stain TO-PRO™-3 Iodide (1:10,000; Invitrogen) was then added, and we subsequently proceeded to fluorescence-activated nuclei sorting (FANS).

In a first sorting step, we sorted 1,000 nuclei/well into a 96-well plate containing 5 µl of 2× Tagmentation Buffer (20 mM Tris–HCl pH 7.5, 20 mM $MgCl_2$, 140 µM PitStop 2 (Sigma-Aldrich), 20% DMF) and 5 µl of nuclear isolation buffer (10 mM Tris–HCl pH 7.5, 10 mM NaCl, 3 mM $MgCl_2$, 0.1% IGEPAL CA-630 (Sigma-Aldrich), 1× protease inhibitors cocktail). Next, 4 µl of a uniquely indexed transposome were added to each well, and the plate was incubated at 55°C for 30 min in a thermocycler for nuclear tagmentation. After this step, nuclei from the 96 wells were pooled, mixed, and filtered through a 40-µm cell strainer again. Fresh TO-PRO™-3 Iodide (1:10,000; Invitrogen) was added for the second sorting step.

In this second sorting, we sorted 22 nuclei/well in a full 96-well plate containing 5 µl of Zymo Digestion Reagent (2.5 µl of Zymo M-Digestion Buffer, 2.25 µl of nuclease-free H_2O, and 0.25 µl of Proteinase K), following the manufacturer's instructions of the EZ-96 DNA Methylation-Direct MagPrep Kit (Zymo Research). Only 10 nuclei were sorted in three wells, where 35 pg of a barcoded-lambda DNA were added as spike-in controls. The plate was incubated for 4 h at 50°C for nuclear digestion. Bisulfite conversion was then performed by adding 32.5 µl of Zymo CT Conversion Reagent in each well and incubating the plate according to the manufacturer's instructions (98°C for 8 min and 64°C for 3.5 h).

After conversion, bisulfite-converted DNA was desulphonated, washed, and recovered following the manufacturer's instructions. In brief, 5 µl of Zymo M-Binding Beads diluted in 150 µl of Zymo M-Binding Buffer was added to each well, and the plate was incubated for 5 min at room temperature for allowing the DNA to attach to the beads. Beads were then washed with 80% ethanol, resuspended in 50 µl of Zymo M-Desulphonation Buffer, and incubated at room temperature for 15 min. Beads were then washed again in 80% ethanol, and DNA was subsequently eluted in 25 µl of Zymo M-Elution Buffer. Eluted DNA was transferred to a 96-well plate containing 16 µl of nuclease-free H_2O, 5 µl of 10× NEBuffer 2.1 (New England Biolabs), 2 µl of 10 mM dNTP mix, and 2 µl of 9-nucleotide random primers (Mulqueen *et al*, 2018). DNA was subjected to four rounds of linear amplification in a thermocycler by incubating at 95°C for 45 s to achieve denaturation. The plate was quickly placed on ice, and 10 U of Klenow (3′-5′ exo-) polymerase (Biozym Scientific) was added per well. The plate was placed again in the thermocycler for incubation at 4°C for 5 min, followed by incubation at 37°C for 90 min. Importantly, the temperature was increased to 37°C by 1°C/15 s. In each amplification round, fresh reagents were added to each well (1.25 µl of 4× NEBuffer 2.1(New England Biolabs), 1 µl of 10 µM dNTP mix, and 1 µl of 9-nucleotide random primers (Mulqueen *et al*, 2018)) as well as 10 U of Klenow (3'-5' exo-) polymerase (Biozym Scientific).

Amplified material was subsequently purified using AMPureXP beads (Beckman Coulter) at a 1.1× volume. DNA was attached to the beads, washed with 80% ethanol, and eluted in 21 µl of EB Buffer (Qiagen). Eluted DNA was added to a 96-well plate containing 25 µl of 2× KAPA HiFi HotStart ReadyMix (Roche), 2 µl of 10 µM i7 index PCR primer, and 2 µl of i5 index PCR primer. Index PCR reaction was performed in a thermocycler by incubating at 95°C for 2 min, then performing 18 cycles at 94°C for 80 s and, finally, incubating at 65°C for 30 s and at 72°C for 30 s. The number of cycles was determined by a qPCR reaction performed with additional wells and could be up to 21 cycles. Libraries were then pooled to achieve a particular cell number and purified using a double-sided size selection step with AMPureXP Beads (0.6×–0.8×; Beckman Coulter). The library was subsequently quantified with Qubit dsDNA HS Assay Kit (Invitrogen), and fragment size was assessed using D5000 or D1000 ScreenTapes (Agilent Technologies). Paired-end (150 bp) sequencing (100 cycles) was finally performed with a NextSeq550 High-Output (Illumina) system following a custom-made recipe (Read 1: 100 imaged cycles; Index Read 1: 10 imaged cycles; Index Read 2: 11 imaged cycles, 16 dark cycles and 10 imaged cycles). Primer sequences for library preparation and sequencing are shown in Appendix Table S1.

sci-MET data analysis

All single cells displaying a minimum of 100,000 sequencing reads were kept for further analysis, which resulted in 554 cells. Reads corresponding to these cells were trimmed by removing stretches of bases with a quality score of < 30 at the end of the reads. Trimmed reads were mapped using bsmap (Xi & Li, 2009). As a reference sequence for the bisulfite mapping, we used the hg19 assembly of the human genome. In order to achieve comparability with the binary nature of single-cell methylation values, the β values of bulk methylomes were binarized by setting every value ≥ 0.5 to 1 and every value < 0.5 to 0. Methylation data of all 554 single cells were then combined with the binarized data of the 67 bulk DNA methylomes from AK, cSCC and healthy epidermal samples, and a 2-dimensional multidimensional scaling (MDS) analysis was performed. For the ChromHMM analysis, we used the Chromatin State Segmentation by HMM from ENCODE/Broad for epidermal keratinocytes (NHEK) (Ernst *et al*, 2011), provided by the UCSC genome server (http://genome.ucsc.edu). Methylation values of all

© 2022 The Authors

CpGs located within each of the 15 types of genomic segments were averaged for each sci-MET cell-type and EPIC tumor subclass.

Bulk DNA methylation data deconvolution

Cell fraction estimation in bulk epidermal tumor methylation data was performed using the EpiSCORE R package (version 0.9.2) (Teschendorff *et al*, 2020). We used 15,000 keratinocytes from the integrated scRNA-seq dataset as the input to generate a gene expression reference matrix with a maximum marker specificity score (MSS) of 3. Only keratinocyte populations involved in the differentiation trajectory defined by RNA velocity and also present in the single-cell multi-omics dataset were included in this analysis. The gene expression reference matrix comprised 784 marker genes and was subsequently validated on a downsampled dataset containing 10,000 keratinocytes, showing an overall cell prediction accuracy of 74%. EpiSCORE then compared the expression-based reference gene matrix to two available datasets containing paired gene expression and DNA methylation data (Epigenomics Roadmap and SCM2) to build an imputed reference DNA methylation matrix for human epidermis, which contained 74 genes. This imputed matrix was then used to estimate cell fractions in our bulk epidermal tumor methylation datasets.

Immunofluorescence stainings

Immunofluorescence stainings were performed with 4 μm sections obtained from nine cSCC and one *in situ* cSCC (BD) previously stratified based on their cells-of-origin, corresponding to five keratinocyte-like and five EpSC-like tumors. Briefly, sections were deparaffinized in xylene and rehydrated in a gradient of ethanol and distilled water prior to heat-induced antigen retrieval. To this end, slides were incubated for 30 min at 95°C in a water bath in 10 mM citrate buffer (pH 6.0) containing 0.05% Tween-20. Subsequently, non-specific antibody binding was blocked by incubation with 10% normal goat serum for 1 h, followed by overnight incubation with primary antibodies diluted in blocking solution at 4°C. Primary antibodies used were rabbit anti-ZEB2 (Sigma-Aldrich, HPA003456, 1:100) and mouse anti-TP63 (Abcam, ab735, 1:100). After washing with PBS with 0.1% Tween-20, sections were then incubated with corresponding Alexa Fluor-conjugated secondary antibodies (Life Technologies) for 2 h at room temperature. Nuclear counterstaining was performed with DAPI, and slides were mounted using ProLong Gold Antifade Mountant (ThermoFisher). Images were taken with an Olympus VS200 slide scanner system (Olympus) using a 40× oil immersion lens and were further processed using the Fiji software (Schindelin *et al*, 2012).

The percentage of ZEB2-positive tumor nuclei (TP63-positive) was calculated in three different regions per sample, counting at least 500 tumor cells per region. Statistical analysis was performed using an unpaired two-sided *t*-test using each region as an independent value.

Data availability

The datasets generated in this study are available from the following databases:

- scMultiome, scRNA-seq and sci-MET: Gene Expression Omnibus GSE207337 (https://www.ncbi.nlm.nih.gov/geo/query/acc.cgi?acc=GSE207337).
- Bulk DNA methylation data: ArrayExpress E-MTAB-11856 (https://www.ebi.ac.uk/arrayexpress/experiments/E-MTAB-11856)

Expanded View for this article is available online.

Acknowledgments

We gratefully acknowledge the help of Julika Dick, Enrico Streit, Patrick Gholam, and David Deltgen for dermatosurgery, and the clinical staff of the Department of Dermatology at Heidelberg University Hospital. We thank the Genomics and Proteomics Core Facility of the DKFZ for their crucial contribution to the implementation of the sci-MET methodology. We also thank Fabio Tabone (Tissue Bank of the National Center for Tumor Diseases (NCT), Heidelberg, Germany) for expert technical assistance, and Claudia Robens for bioinformatics support in the scRNA-seq analysis. This work was supported by a grant (EpiSkin—MET-ID09) from the Baden-Württemberg Stiftung gGmbH to GR and FL. Open access funding enabled and organized by Projekt DEAL.

Author contributions

Manuel Rodríguez-Paredes: Conceptualization; formal analysis; supervision; writing—original draft; project administration; writing—review and editing. **Llorenç Solé-Boldo:** Data curation; software; formal analysis; validation; investigation; visualization; writing—original draft; writing—review and editing. **Günter Raddatz:** Software; formal analysis; validation. **Julian Gutekunst:** Software; formal analysis. **Oliver Gilliam:** Software; formal analysis. **Felix Bormann:** Software; formal analysis. **Michelle S Liberio:** Methodology. **Daniel Hasche:** Methodology. **Wiebke Antonopoulos:** Resources; methodology. **Jan-Philipp Mallm:** Methodology. **Anke S Lonsdorf:** Resources; formal analysis; investigation. **Frank Lyko:** Conceptualization; formal analysis; supervision; funding acquisition; writing—original draft; project administration; writing—review and editing.

Disclosure and competing interests statement

The authors declare that they have no conflict of interest.

References

Adey A, Kitzman JO, Burton JN, Daza R, Kumar A, Christiansen L, Ronaghi M, Amini S, Gunderson KL, Steemers FJ *et al* (2014) In vitro, long-range sequence information for de novo genome assembly via transposase contiguity. *Genome Res* 24: 2041–2049

Aibar S, González-Blas CB, Moerman T, Huynh-Thu VA, Imrichova H, Hulselmans G, Rambow F, Marine JC, Geurts P, Aerts J *et al* (2017) SCENIC: single-cell regulatory network inference and clustering. *Nat Methods* 14: 1083–1086

Al-Eitan LN, Alghamdi MA, Tarkhan AH, Al-Qarqaz FA (2020) Genome-wide identification of methylated CpG sites in nongenital cutaneous warts. *BMC Med Genomics* 13: 100

Amini S, Pushkarev D, Christiansen L, Kostem E, Royce T, Turk C, Pignatelli N, Adey A, Kitzman JO, Vijayan K *et al* (2014) Haplotype-resolved whole-genome sequencing by contiguity-preserving transposition and combinatorial indexing. *Nat Genet* 46: 1343–1349

Apalla Z, Lallas A, Sotiriou E, Lazaridou E, Ioannides D (2017a) Epidemiological trends in skin cancer. *Dermatol Pract Concept* 7: 1–6

© 2022 The Authors

Apalla Z, Nashan D, Weller RB, Castellsagué X (2017b) Skin cancer: epidemiology, disease burden, pathophysiology, diagnosis, and therapeutic approaches. *Dermatol Ther (Heidelb)* 7: 5 – 19

Aryee MJ, Jaffe AE, Corrada-Bravo H, Ladd-Acosta C, Feinberg AP, Hansen KD, Irizarry RA (2014) Minfi: A flexible and comprehensive Bioconductor package for the analysis of Infinium DNA methylation microarrays. *Bioinformatics* 30: 1363 – 1369

Bartoš V, Kullová M (2018) Non-melanoma skin cancer – a clinicopathological study of patients with basal cell carcinoma and squamous cell carcinoma. *Klin Onkol* 31: 40 – 45

Bergen V, Lange M, Peidli S, Wolf FA, Theis FJ (2020) Generalizing RNA velocity to transient cell states through dynamical modeling. *Nat Biotechnol* 38: 1408 – 1414

Bernstein BE, Kamal M, Lindblad-Toh K, Bekiranov S, Bailey DK, Huebert DJ, McMahon S, Karlsson EK, Kulbokas EJ, Gingeras TR *et al* (2005) Genomic maps and comparative analysis of histone modifications in human and mouse. *Cell* 120: 169 – 181

Blanpain C, Fuchs E (2009) Epidermal homeostasis: a balancing act of stem cells in the skin. *Nat Rev Mol Cell Biol* 10: 207 – 217

Bock C, Beerman I, Lien WH, Smith ZD, Gu H, Boyle P, Gnirke A, Fuchs E, Rossi DJ, Meissner A (2012) DNA methylation dynamics during *in vivo* differentiation of blood and skin stem cells. *Mol Cell* 47: 633 – 647

Borrelli S, Testoni B, Callari M, Alotto D, Castagnoli C, Romano RA, Sinha S, Viganò AM, Mantovani R (2007) Reciprocal regulation of p63 by C/EBP delta in human keratinocytes. *BMC Mol Biol* 8: 1 – 14

Burton KA, Ashack KA, Khachemoune A (2016) Cutaneous squamous cell carcinoma: a review of high-risk and metastatic disease. *Am J Clin Dermatol* 17: 491 – 508

Cheng JB, Sedgewick AJ, Finnegan AI, Benz SC, Song JS, Cho RJ (2018a) Transcriptional programming of normal and inflamed human epidermis at single-cell resolution. *Cell Rep* 25: 871 – 883

Cheng JB, Sedgewick AJ, Finnegan AI, Benz SC, Song JS, Cho RJ (2018b) European Genome-phenome Archive EGAS00001002927 (https://ega-archive.org/studies/EGAS00001002927) [DATASET]

Conforti C, Pizzichetta MA, Vichi S, Toffolutti F, Serraino D, di Meo N, Giuffrida R, Deinlein T, Giacomel J, Rosendahl C *et al* (2021) Sclerodermiform basal cell carcinomas vs. other histotypes: analysis of specific demographic, clinical and dermatoscopic features. *J Eur Acad Dermatol Venereol* 35: 79 – 87

Creyghton MP, Cheng AW, Welstead GG, Kooistra T, Carey BW, Steine EJ, Hanna J, Lodato MA, Frampton GM, Sharp PA *et al* (2010) Histone H3K27ac separates active from poised enhancers and predicts developmental state. *Proc Natl Acad Sci U S A* 107: 21931 – 21936

Dai J, Brooks Y, Lefort K, Getsios S, Dotto GP (2013) The retinoid-related orphan receptor RORα promotes keratinocyte differentiation via FOXN1. *PLoS One* 8: e70392

Davalos V, Moutinho C, Villanueva A, Boque R, Silva P, Carneiro F, Esteller M (2012) Dynamic epigenetic regulation of the microRNA-200 family mediates epithelial and mesenchymal transitions in human tumorigenesis. *Oncogene* 31: 2062 – 2074

Deplus R, Blanchon L, Rajavelu A, Boukaba A, Defrance M, Luciani J, Rothé F, Dedeurwaerder S, Denis H, Brinkman AB *et al* (2014) Regulation of DNA methylation patterns by CK2-mediated phosphorylation of Dnmt3a. *Cell Rep* 8: 743 – 753

Duran-Ferrer M, Clot G, Nadeu F, Beekman R, Baumann T, Nordlund J, Marincevic-Zuniga Y, Lönnerholm G, Rivas-Delgado A, Martín S *et al* (2020) The proliferative history shapes the DNA methylome of B-cell tumors and predicts clinical outcome. *Nat Cancer* 1: 1066 – 1081

Ernst J, Kheradpour P, Mikkelsen TS, Shoresh N, Ward LD, Epstein CB, Zhang X, Wang L, Issner R, Coyne M *et al* (2011) Mapping and analysis of chromatin state dynamics in nine human cell types. *Nature* 473: 43 – 49

Estrella NL, Desjardins CA, Nocco SE, Clark AL, Maksimenko Y, Naya FJ (2015) MEF2 transcription factors regulate distinct gene programs in mammalian skeletal muscle differentiation. *J Biol Chem* 290: 1256 – 1268

Fitzmaurice C, Abate D, Abbasi N, Abbastabar H, Abd-Allah F, Abdel-Rahman O, Abdelalim A, Abdoli A, Abdollahpour I, Abdulle ASM *et al* (2019) Global, regional, and national cancer incidence, mortality, years of life lost, years lived with disability, and disability-adjusted life-years for 29 cancer groups, 1990 to 2017: a systematic analysis for the global burden of disease study. *JAMA Oncol* 5: 1749 – 1768

Gao CF, Xie Q, Su YL, Koeman J, Khoo SK, Gustafson M, Knudsen BS, Hay R, Shinomiya N, van de Woude GF (2005) Proliferation and invasion: plasticity in tumor cells. *Proc Natl Acad Sci U S A* 102: 10528 – 10533

Gonzales KAU, Fuchs E (2017) Skin and its regenerative powers: an Alliance between stem cells and their niche. *Dev Cell* 43: 387 – 401

Greenberg MVC, Bourc'his D (2019) The diverse roles of DNA methylation in mammalian development and disease. *Nat Rev Mol Cell Biol* 20: 590 – 607

Gregory PA, Bert AG, Paterson EL, Barry SC, Tsykin A, Farshid G, Vadas MA, Khew-Goodall Y, Goodall GJ (2008) The miR-200 family and miR-205 regulate epithelial to mesenchymal transition by targeting ZEB1 and SIP1. *Nat Cell Biol* 10: 593 – 601

Gulati N, Krueger JG, Suárez-Fariñas M, Mitsui H (2013) Creation of differentiation-specific genomic maps of human epidermis through laser capture microdissection. *J Invest Dermatol* 133: 2640 – 2642

Guo H, Hu B, Yan L, Yong J, Wu Y, Gao Y, Guo F, Hou Y, Fan X, Dong J *et al* (2016) DNA methylation and chromatin accessibility profiling of mouse and human fetal germ cells. *Cell Res* 27: 165 – 183

Hansen K (2017) IlluminaHumanMethylationEPICanno.ilm10b4.hg19: annotation for Illumina's EPIC methylation arrays. R package version 0.6.0

Hecht I, Natan S, Zaritsky A, Levine H, Tsarfaty I, Ben-Jacob E (2015) The motility-proliferation-metabolism interplay during metastatic invasion. *Sci Rep* 5: 1 – 10

Hervás-Marín D, Higgins F, Sanmartín O, López-Guerrero JA, Bañó MC, Igual JC, Quilis I, Sandoval J (2019a) Genome wide DNA methylation profiling identifies specific epigenetic features in highrisk cutaneous squamous cell carcinoma. *PLoS One* 14: e0223341

Hervás-Marín D, Higgins F, Sanmartín O, López-Guerrero JA, Bañó MC, Igual JC, Quilis I, Sandoval J (2019b) Array Express (https://www.ebi.ac.uk/arrayexpress/experiments/E-MTAB-8542) [DATASET]

Hoadley KA, Yau C, Hinoue T, Wolf DM, Lazar AJ, Drill E, Shen R, Taylor AM, Cherniack AD, Thorsson V *et al* (2018) Cell-of-origin patterns dominate the molecular classification of 10,000 tumors from 33 types of cancer. *Cell* 173: 291 – 304

Hoek KS, Eichhoff OM, Schlegel NC, Döbbeling U, Kobert N, Schaerer L, Hemmi S, Dummer R (2008) *In vivo* switching of human melanoma cells between proliferative and invasive states. *Cancer Res* 68: 650 – 656

Ji AL, Rubin AJ, Thrane K, Jiang S, Reynolds DL, Meyers RM, Guo MG, George BM, Mollbrink A, Bergenstråhle J *et al* (2020) Multimodal analysis of composition and spatial architecture in human squamous cell carcinoma. *Cell* 182: 497 – 514

Jones PA, Baylin SB (2007) The epigenomics of cancer. *Cell* 128: 683 – 692

Karemaker ID, Vermeulen M (2018) Single-cell DNA methylation profiling: technologies and biological applications. *Trends Biotechnol* 36: 952 – 965

Kulis M, Queirós AC, Beekman R, Martín-Subero JI (2013) Intragenic DNA methylation in transcriptional regulation, normal differentiation and cancer. *Biochim Biophys Acta* 1829: 1161 – 1174

 © 2022 The Authors

Kumar N, Mishra B, Athar M, Mukhtar S (2021) Inference of gene regulatory network from single-cell transcriptomic data using pySCENIC. *Methods Mol Biol* 2328: 171 – 182

Kypriotou M, Huber M, Hohl D (2012) The human epidermal differentiation complex: Cornified envelope precursors, S100 proteins and the "fused genes" family. *Exp Dermatol* 21: 643 – 649

Lawrence M, Huber W, Pagès H, Aboyoun P, Carlson M, Gentleman R, Morgan MT, Carey VJ (2013) Software for computing and annotating genomic ranges. *PLoS Comput Biol* 9: e1003118

Lee B, Villarreal-Ponce A, Fallahi M, Ovadia J, Sun P, Yu QC, Ito S, Sinha S, Nie Q, Dai X (2014) Transcriptional mechanisms link epithelial plasticity to adhesion and differentiation of epidermal progenitor cells. *Dev Cell* 29: 47 – 58

Li D, Shu X, Zhu P, Pei D (2021) Chromatin accessibility dynamics during cell fate reprogramming. *EMBO Rep* 22: e51644

Lopez RG, Garcia-Silva S, Moore SJ, Bereshchenko O, Martinez-Cruz AB, Ermakova O, Kurz E, Paramio JM, Nerlov C (2009) C/EBPalpha and beta couple interfollicular keratinocyte proliferation arrest to commitment and terminal differentiation. *Nat Cell Biol* 11: 1181 – 1190

Lyko F (2018) The DNA methyltransferase family: A versatile toolkit for epigenetic regulation. *Nat Rev Genet* 19: 81 – 92

Maeda K (2017) New method of measurement of epidermal turnover in humans. *Cosmetics* 4: 47

la Manno G, Soldatov R, Zeisel A, Braun E, Hochgerner H, Petukhov V, Lidschreiber K, Kastriti ME, Lönnerberg P, Furlan A *et al* (2018) RNA velocity of single cells. *Nature* 560: 494 – 498

Maytin EV, Habener JF (1998) Transcription factors C/EBP alpha, C/EBP beta, and CHOP (Gadd153) expressed during the differentiation program of keratinocytes *in vitro* and *in vivo*. *J Invest Dermatol* 110: 238 – 246

Michel M, Török N, Godbout MJ, Lussier M, Gaudreau P, Royal A, Germain L (1996) Keratin 19 as a biochemical marker of skin stem cells in vivo and *in vitro*: Keratin 19 expressing cells are differentially localized in function of anatomic sites, and their number varies with donor age and culture stage. *J Cell Sci* 109: 1017 – 1028

Mlacki M, Darido C, Jane SM, Wilanowski T (2014) Loss of grainy head-like 1 is associated with disruption of the epidermal barrier and squamous cell carcinoma of the skin. *PLoS One* 9: e89247

Moran S, Martínez-Cardús A, Sayols S, Musulén E, Balañá C, Estival-Gonzalez A, Moutinho C, Heyn H, Diaz-Lagares A, de Moura MC *et al* (2016) Epigenetic profiling to classify cancer of unknown primary: a multicentre, retrospective analysis. *Lancet Oncol* 17: 1386 – 1395

Moreci RS, Lechler T (2020) Epidermal structure and differentiation. *Curr Biol* 30: R144 – R149

Mudigonda T, Pearce DJ, Yentzer BA, Williford P, Feldman SR (2010) The economic impact of non-melanoma skin cancer: a review. *J Natl Compr Canc Netw* 8: 888 – 896

Mulqueen RM, Pokholok D, Norberg SJ, Torkenczy KA, Fields AJ, Sun D, Sinnamon JR, Shendure J, Trapnell C, O'Roak BJ *et al* (2018) Highly scalable generation of DNA methylation profiles in single cells. *Nat Biotechnol* 36: 428 – 431

Neves R, Scheel C, Weinhold S, Honisch E, Iwaniuk KM, Trompeter HI, Niederacher D, Wernet P, Santourlidis S, Uhrberg M (2010) Role of DNA methylation in miR-200c/141 cluster silencing in invasive breast cancer cells. *BMC Res Notes* 3: 219

Orford K, Kharchenko P, Lai W, Dao MC, Worhunsky DJ, Ferro A, Janzen V, Park PJ, Scadden DT (2008) Differential H3K4 methylation identifies developmentally poised hematopoietic genes. *Dev Cell* 14: 798 – 809

Park SM, Gaur AB, Lengyel E, Peter ME (2008) The miR-200 family determines the epithelial phenotype of cancer cells by targeting the E-cadherin repressors ZEB1 and ZEB2. *Genes Dev* 22: 894 – 907

Picelli S, Björklund AK, Reinius B, Sagasser S, Winberg G, Sandberg R (2014) Tn5 transposase and tagmentation procedures for massively scaled sequencing projects. *Genome Res* 24: 2033 – 2040

Pliner HA, Packer JS, McFaline-Figueroa JL, Cusanovich DA, Daza RM, Aghamirzaie D, Srivatsan S, Qiu X, Jackson D, Minkina A *et al* (2018) Cicero predicts cis-regulatory DNA interactions from single-cell chromatin accessibility data. *Mol Cell* 71: 858 – 871

Pontiggia L, Biedermann T, Meuli M, Widmer D, Böttcher-Haberzeth S, Schiestl C, Schneider J, Braziulis E, Montaño I, Meuli-Simmen C *et al* (2009) Markers to evaluate the quality and self-renewing potential of engineered human skin substitutes in vitro and after transplantation. *J Invest Dermatol* 129: 480 – 490

Rachidi W, Harfourche G, Lemaitre G, Amiot F, Vaigot P, Martin MT (2007) Sensing radiosensitivity of human epidermal stem cells. *Radiother Oncol* 83: 267 – 276

Ratushny V, Gober MD, Hick R, Ridky TW, Seykora JT (2012) From keratinocyte to cancer: the pathogenesis and modeling of cutaneous squamous cell carcinoma. *J Clin Invest* 122: 464 – 472

Ren X, Kuan PF (2019) methylGSA: a Bioconductor package and shiny app for DNA methylation data length bias adjustment in gene set testing. *Bioinformatics* 35: 1958 – 1959

Rinaldi L, Datta D, Serrat J, Morey L, Solanas G, Avgustinova A, Blanco E, Pons JI, Matallanas D, von Kriegsheim A *et al* (2016) Dnmt3a and Dnmt3b associate with enhancers to regulate human epidermal stem cell homeostasis. *Cell Stem Cell* 19: 491 – 501

Rodríguez-Paredes M, Bormann F, Raddatz G, Gutekunst J, Lucena-Porcel C, Köhler F, Wurzer E, Schmidt K, Gallinat S, Wenck H *et al* (2018a) Methylation profiling identifies two subclasses of squamous cell carcinoma related to distinct cells of origin. *Nat Commun* 9: 1 – 9

Rodríguez-Paredes M, Bormann F, Raddatz G, Gutekunst J, Lucena-Porcel C, Köhler F, Wurzer E, Schmidt K, Gallinat S, Wenck H *et al* (2018b) Array Express E-MTAB-5738 (https://www.ebi.ac.uk/arrayexpress/experiments/E-MTAB-5738) [DATASET]

Sand M, Bromba A, Sand D, Gambichler T, Hessam S, Becker JC, Stockfleth E, Meyer T, Bechara FG (2019a) Dicer sequencing, whole genome methylation profiling, mRNA and smallRNA sequencing analysis in basal cell carcinoma. *Cell Physiol Biochem* 53: 760 – 773

Sand M, Bromba A, Sand D, Gambichler T, Hessam S, Becker JC, Stockfleth E, Meyer T, Bechara FG (2019b) Gene Expression Omnibus GSE128795 (https://www.ncbi.nlm.nih.gov/geo/query/acc.cgi?acc=GSE128795) [DATASET]

Schep AN, Wu B, Buenrostro JD, Greenleaf WJ (2017) chromVAR: Inferring transcription-factor-associated accessibility from single-cell epigenomic data. *Nat Methods* 14: 975 – 978

Schindelin J, Arganda-Carreras I, Frise E, Kaynig V, Longair M, Pietzsch T, Preibisch S, Rueden C, Saalfeld S, Schmid B *et al* (2012) Fiji: an open-source platform for biological-image analysis. *Nat Methods* 9: 676 – 682

Sen GL, Reuter JA, Webster DE, Zhu L, Khavari PA (2010) DNMT1 maintains progenitor function in self-renewing somatic tissue. *Nature* 463: 563 – 567

Soares E, Zhou H (2018) Master regulatory role of p63 in epidermal development and disease. *Cell Mol Life Sci* 75: 1179

Stang A, Khil L, Kajüter H, Pandeya N, Schmults CD, Ruiz ES, Karia PS, Green AC (2019) Incidence and mortality for cutaneous squamous cell

carcinoma: Comparison across three continents. *J Eur Acad Dermatol Venereol* 33: 6 – 10

Stuart T, Butler A, Hoffman P, Hafemeister C, Papalexi E, Mauck WM, Hao Y, Stoeckius M, Smibert P, Satija R (2019) Comprehensive integration of single-cell data. *Cell* 177: 1888 – 1902

Stuart T, Srivastava A, Madad S, Lareau CA, Satija R (2021) Single-cell chromatin state analysis with Signac. *Nat Methods* 18: 1333 – 1341

Teschendorff AE (2020) A comparison of epigenetic mitotic-like clocks for cancer risk prediction. *Genome Med* 12: 56

Teschendorff AE, Zhu T, Breeze CE, Beck S (2020) EPISCORE: Cell type deconvolution of bulk tissue DNA methylomes from single-cell RNA-seq data. *Genome Biol* 21: 1

Truong AB, Kretz M, Ridky TW, Kimmel R, Khavari PA (2006) p63 regulates proliferation and differentiation of developmentally mature keratinocytes. *Genes Dev* 20: 3185

Wiklund ED, Bramsen JB, Hulf T, Dyrskjøt L, Ramanathan R, Hansen TB, Villadsen SB, Gao S, Ostenfeld MS, Borre M *et al* (2011) Coordinated epigenetic repression of the miR-200 family and miR-205 in invasive bladder cancer. *Int J Cancer* 128: 1327 – 1334

Wysocka J, Reilly PT, Herr W (2001) Loss of HCF-1–chromatin association precedes temperature-induced growth arrest of tsBN67 cells. *Mol Cell Biol* 21: 3820 – 3829

Xi Y, Li W (2009) BSMAP: whole genome bisulfite sequence MAPping program. *BMC Bioinformatics* 10: 1 – 9

Yang Z, Wong A, Kuh D, Paul DS, Rakyan VK, Leslie RD, Zheng SC, Widschwendter M, Beck S, Teschendorff AE (2016) Correlation of an epigenetic mitotic clock with cancer risk. *Genome Biol* 17: 205

Yuan Y, Park J, Feng A, Awasthi P, Wang Z, Chen Q, Iglesias-Bartolome R (2020) YAP1/TAZ-TEAD transcriptional networks maintain skin homeostasis by regulating cell proliferation and limiting KLF4 activity. *Nat Commun* 11: 1 – 14

Zhu B, Carmichael RE, Solabre Valois L, Wilkinson KA, Henley JM (2018) The transcription factor MEF2A plays a key role in the differentiation/maturation of rat neural stem cells into neurons. *Biochem Biophys Res Commun* 500: 645

Zhu LJ, Gazin C, Lawson ND, Pagès H, Lin SM, Lapointe DS, Green MR (2010) ChIPpeakAnno: a Bioconductor package to annotate ChIP-seq and ChIP-chip data. *BMC Bioinformatics* 11: 1 – 10

License: This is an open access article under the terms of the Creative Commons Attribution License, which permits use, distribution and reproduction in any medium, provided the original work is properly cited.

© 2022 The Authors

Expanded View Figures

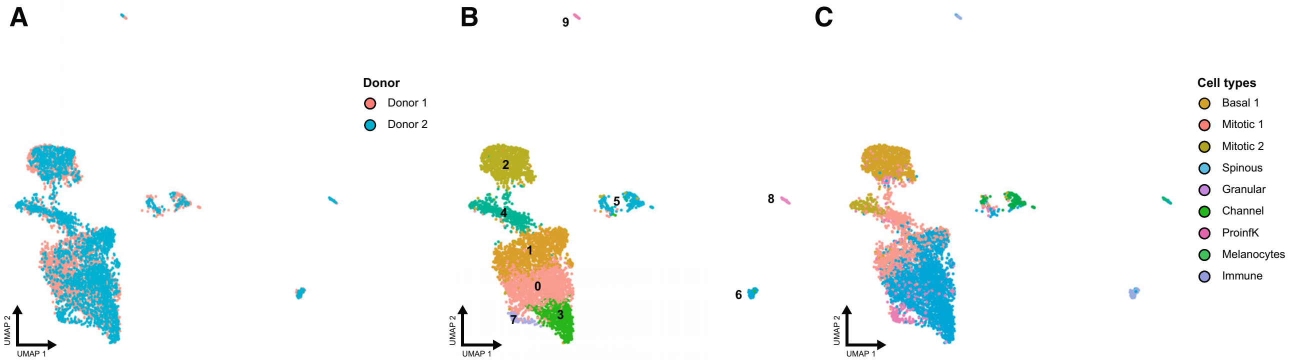

Figure EV1. Unsupervised clustering of the integrated single-cell multi-omics dataset.

A–C Joint UMAP plot depicting both scATAC-seq and scRNA-seq data from 5,565 cells from sun-protected human epidermis ($n = 2$) after data integration. Coloring is according to donor (A), unsupervised clustering based on gene expression (B), and cell-type annotation based on the reference scRNA-seq dataset (C).

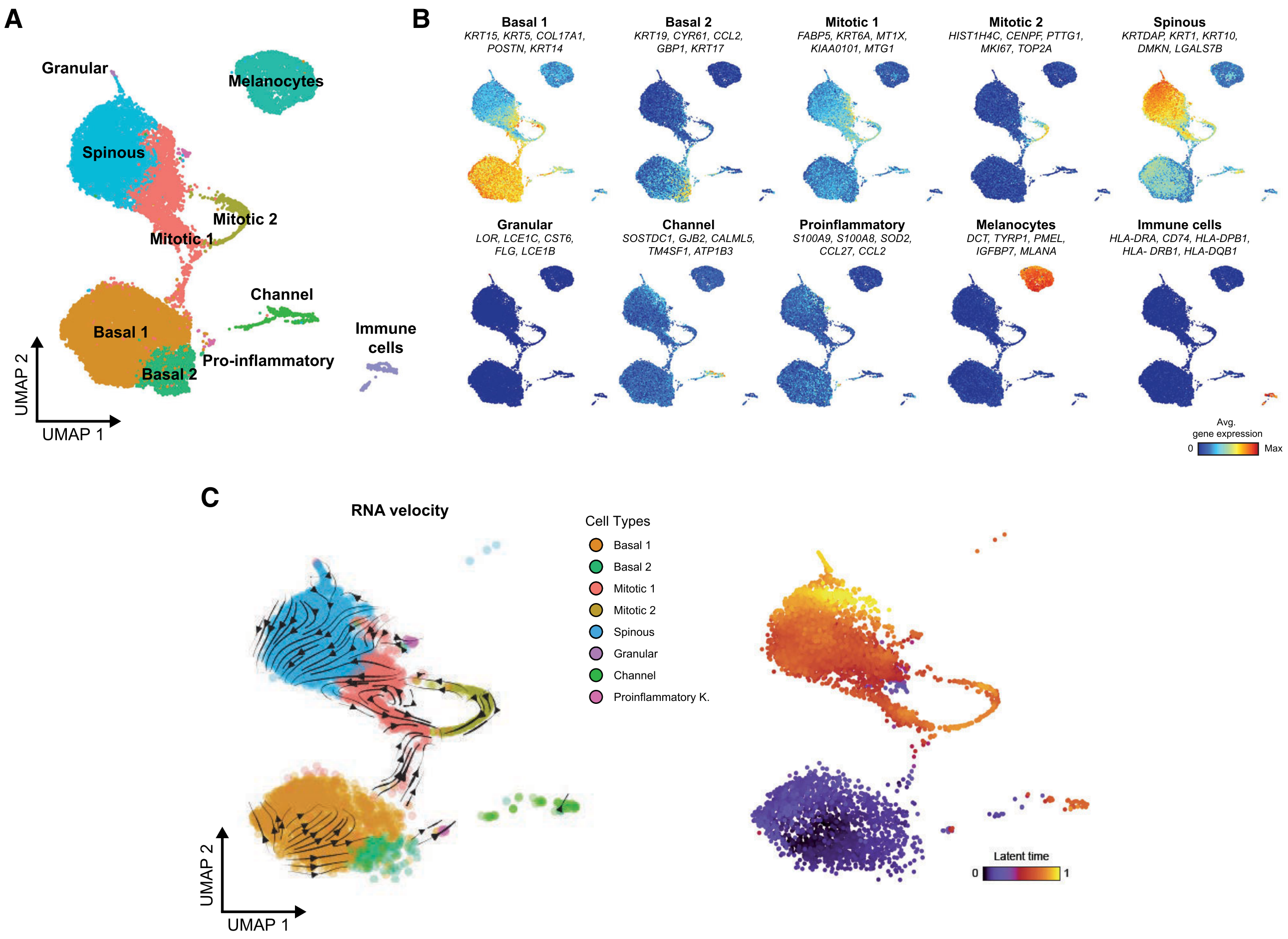

Figure EV2. Single-cell RNA sequencing analysis of the human epidermis.

A Uniform manifold approximation and projection (UMAP) plot depicting single-cell transcriptomes from healthy sun-protected human epidermis (n = 4). Each dot represents a single cell (n = 32,272). Colors depict the six archetypical keratinocyte populations described in the text, as well as other minority cell types (Cheng *et al*, 2018a; Data ref: Cheng *et al*, 2018b).

B Average expression of the top five gene markers defining each cell population projected on the UMAP plot. Red indicates maximum average gene expression, while blue indicates low or no expression of a particular set of genes in log-normalized UMI counts.

C Left: RNA velocities calculated using the 7,068 keratinocytes from the in-house generated dataset of healthy human epidermis, projected onto the UMAP embedding. Right: UMAP plot displaying the latent time calculated by scVelo.

Figure EV3. Cell-of-origin-based tumor stratification strategy can be expanded to other epidermal tumor entities.

A–E Heatmaps displaying unsupervised hierarchical clustering based on the methylation patterns at differentiated keratinocyte-specific peaks of (A) 102 epidermal tumors and healthy controls; (B) Five sBCC samples from Sand *et al* (2019a), Data ref: Sand *et al* (2019b); (C) 12 averaged VV samples from Al-Eitan *et al* (2020) (D) Five AK and 18 cSCC samples from Hervás-Marín *et al* (2019a), Data ref: Hervás-Marín *et al* (2019b); (E) Eight cSCC metastases and three primary metastatic cSCC, always together with the 12 healthy samples from our cohort. Each row represents the average methylation value of all CpGs contained in a particular peak. Heatmaps correspond to the dendrograms shown in Fig 5A–E. AK: actinic keratosis, BCC: basal cell carcinoma, BD: Bowen's disease, cSCC: cutaneous squamous cell carcinoma, sBCC: sclerodermiform basal cell carcinoma, SK: seborrheic keratosis, VV: verruca vulgaris.

 © 2022 The Authors

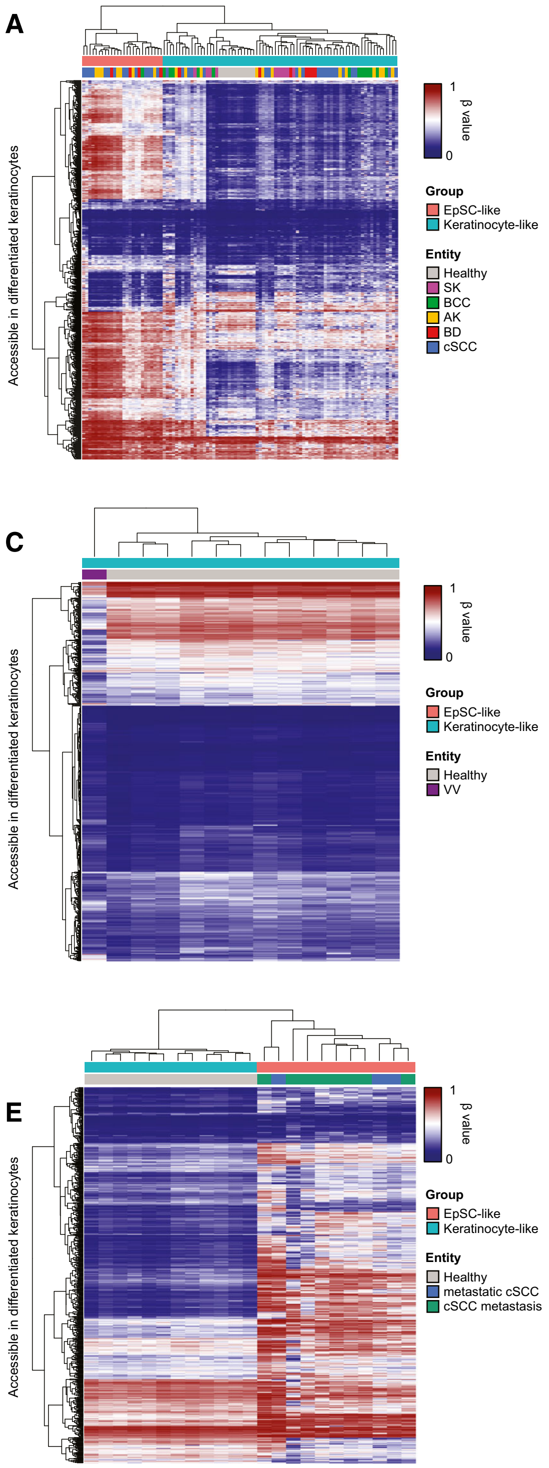
A
B
C
D
E
Accessible in differentiated keratinocytes
β value
1
0
Group
EpSC-like
Keratinocyte-like
Entity
Healthy
SK
BCC
AK
BD
cSCC
sBCC
VV
metastatic cSCC
cSCC metastasis

Figure EV3.

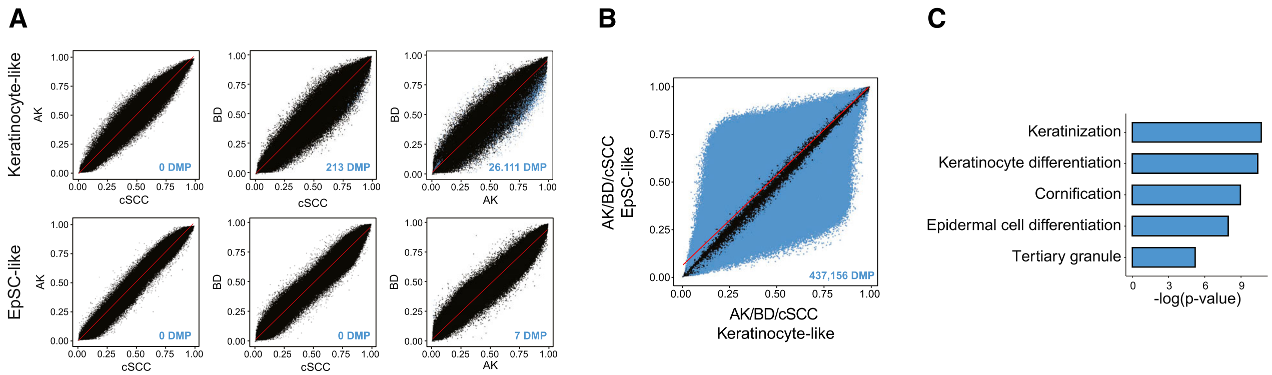

Figure EV4. cSCC and precursor lesions from the same cell-of-origin subclass display almost identical methylomes.

A Scatter plots of pairwise comparisons between AK, BD, and cSCC methylomes from the keratinocyte-like (upper) or EpSC-like (lower) subclass.
B Scatter plot comparing AK, BD, and cSCC methylomes from each cell-of-origin subclass as a unique entity.
C Top five enriched Gene Ontology (GO) terms using genes with differentially methylated promoter regions between AK/BD/cSCC samples from distinct cell-of-origin subclasses.

Data information: In the scatter plots, significantly differentially methylated CpG probes (*P*-value < 0.05, *F*-test) are depicted in blue. AK: actinic keratosis, BD: Bowen's disease, cSCC: cutaneous squamous cell carcinoma, DMP: differentially methylated probes.

 © 2022 The Authors

Expanded View Figures

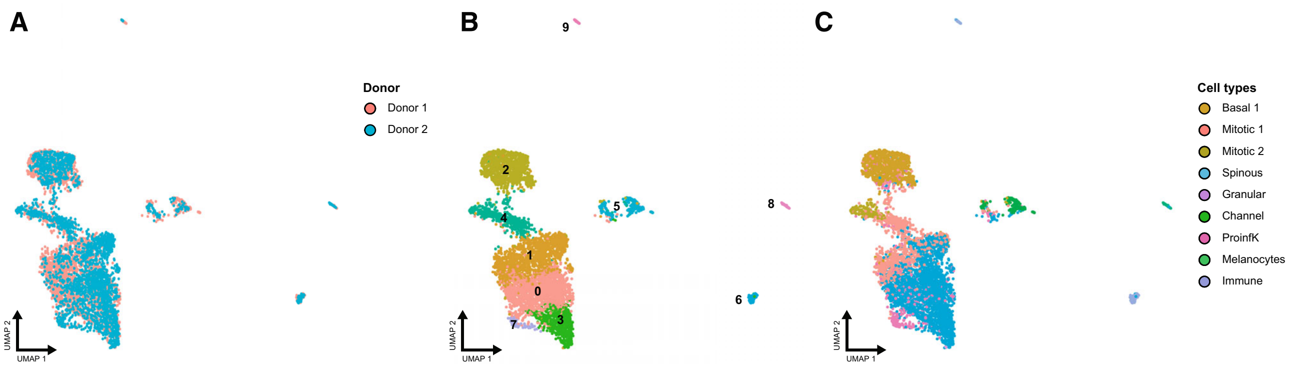

Figure EV1. Unsupervised clustering of the integrated single-cell multi-omics dataset.

A–C Joint UMAP plot depicting both scATAC-seq and scRNA-seq data from 5,565 cells from sun-protected human epidermis (n = 2) after data integration. Coloring is according to donor (A), unsupervised clustering based on gene expression (B), and cell-type annotation based on the reference scRNA-seq dataset (C).

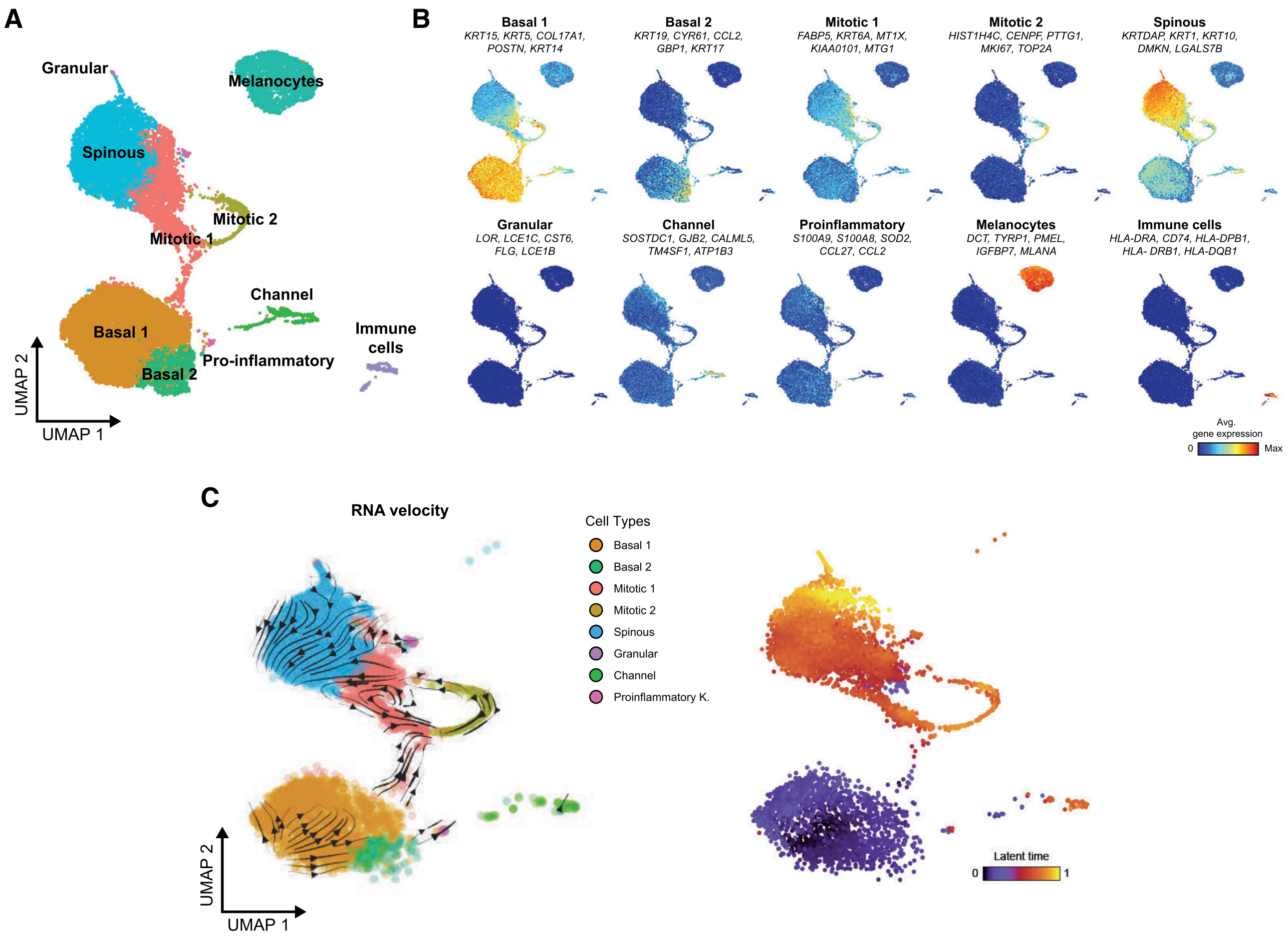

Figure EV2. Single-cell RNA sequencing analysis of the human epidermis.

A Uniform manifold approximation and projection (UMAP) plot depicting single-cell transcriptomes from healthy sun-protected human epidermis (n = 4). Each dot represents a single cell (n = 32,272). Colors depict the six archetypical keratinocyte populations described in the text, as well as other minority cell types (Cheng *et al*, 2018a; Data ref: Cheng *et al*, 2018b).

B Average expression of the top five gene markers defining each cell population projected on the UMAP plot. Red indicates maximum average gene expression, while blue indicates low or no expression of a particular set of genes in log-normalized UMI counts.

C Left: RNA velocities calculated using the 7,068 keratinocytes from the in-house generated dataset of healthy human epidermis, projected onto the UMAP embedding. Right: UMAP plot displaying the latent time calculated by scVelo.

Figure EV3. Cell-of-origin-based tumor stratification strategy can be expanded to other epidermal tumor entities.

A–E Heatmaps displaying unsupervised hierarchical clustering based on the methylation patterns at differentiated keratinocyte-specific peaks of (A) 102 epidermal tumors and healthy controls; (B) Five sBCC samples from Sand *et al* (2019a), Data ref: Sand *et al* (2019b); (C) 12 averaged VV samples from Al-Eitan *et al* (2020) (D) Five AK and 18 cSCC samples from Hervás-Marín *et al* (2019a), Data ref: Hervás-Marín *et al* (2019b); (E) Eight cSCC metastases and three primary metastatic cSCC, always together with the 12 healthy samples from our cohort. Each row represents the average methylation value of all CpGs contained in a particular peak. Heatmaps correspond to the dendrograms shown in Fig 5A–E. AK: actinic keratosis, BCC: basal cell carcinoma, BD: Bowen's disease, cSCC: cutaneous squamous cell carcinoma, sBCC: sclerodermiform basal cell carcinoma, SK: seborrheic keratosis, VV: verruca vulgaris.

© 2022 The Authors

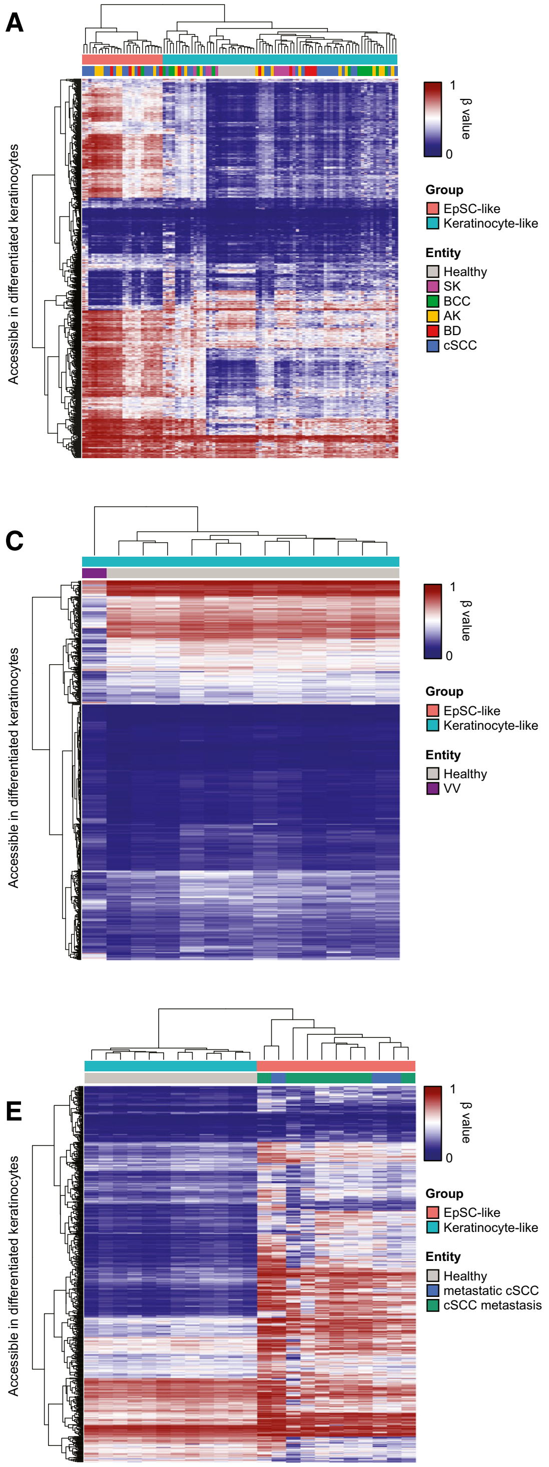
A
Accessible in differentiated keratinocytes
1
β value
0
Group
EpSC-like
Keratinocyte-like
Entity
Healthy
SK
BCC
AK
BD
cSCC
B
Accessible in differentiated keratinocytes
1
β value
0
Group
EpSC-like
Keratinocyte-like
Entity
Healthy
sBCC
C
Accessible in differentiated keratinocytes
1
β value
0
Group
EpSC-like
Keratinocyte-like
Entity
Healthy
VV
D
Accessible in differentiated keratinocytes
1
β value
0
Group
EpSC-like
Keratinocyte-like
Entity
Healthy
AK
cSCC
E
Accessible in differentiated keratinocytes
1
β value
0
Group
EpSC-like
Keratinocyte-like
Entity
Healthy
metastatic cSCC
cSCC metastasis

Figure EV3.

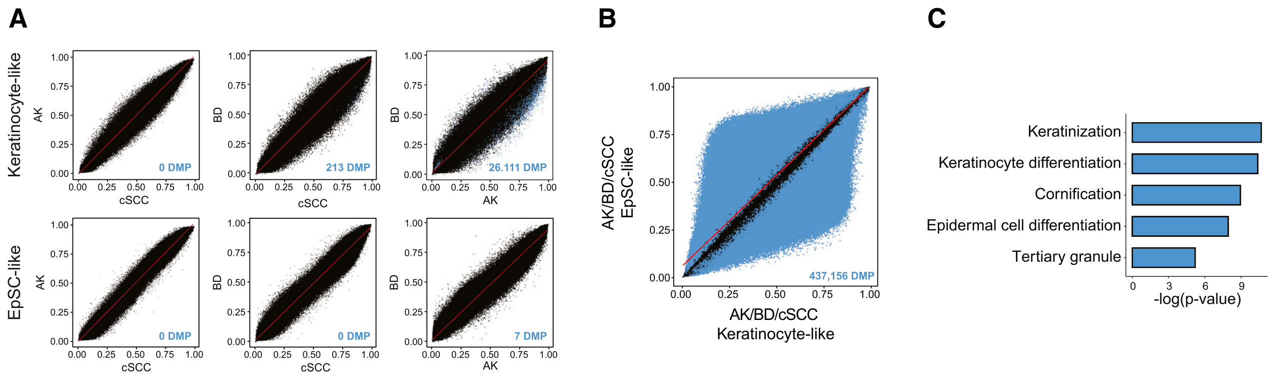

Figure EV4. cSCC and precursor lesions from the same cell-of-origin subclass display almost identical methylomes.

A Scatter plots of pairwise comparisons between AK, BD, and cSCC methylomes from the keratinocyte-like (upper) or EpSC-like (lower) subclass.

B Scatter plot comparing AK, BD, and cSCC methylomes from each cell-of-origin subclass as a unique entity.

C Top five enriched Gene Ontology (GO) terms using genes with differentially methylated promoter regions between AK/BD/cSCC samples from distinct cell-of-origin subclasses.

Data information: In the scatter plots, significantly differentially methylated CpG probes (P-value < 0.05, F-test) are depicted in blue. AK: actinic keratosis, BD: Bowen's disease, cSCC: cutaneous squamous cell carcinoma, DMP: differentially methylated probes.

© 2022 The Authors

Differentiation-related epigenomic changes define clinically distinct keratinocyte cancer subclasses.

Llorenç Solé-Boldo[1], Günter Raddatz[1], Julian Gutekunst[1], Oliver Gilliam[1], Felix Bormann[1], Michelle S. Liberio[2], Daniel Hasche[3], Wiebke Antonopoulos[4,5], Jan-Philipp Mallm[2,6], Anke S. Lonsdorf[7], Manuel Rodríguez-Paredes[1,8*] & Frank Lyko[1*]

[1]Division of Epigenetics, DKFZ-ZMBH Alliance, German Cancer Research Center, 69120 Heidelberg, Germany; [2]Single-cell Open Lab, German Cancer Research Center and Bioquant, 69120 Heidelberg, Germany; [3]Division of Viral Transformation Mechanisms, German Cancer Research Center, 69120 Heidelberg, Germany; [4]Tissue Bank of the National Center for Tumor Diseases (NCT), 69120 Heidelberg, Germany; [5]Institute of Pathology, Heidelberg University Hospital, 69120 Heidelberg, Germany; [6]Division of Chromatin Networks, German Cancer Research Center and Bioquant, 69120 Heidelberg, Germany; [7]Department of Dermatology, University Hospital, Ruprecht-Karls University of Heidelberg, 69120 Heidelberg, Germany; [8]Institute of Toxicology, University Medical Center Mainz, Johannes Gutenberg University, 55131 Mainz, Germany.

*Equal contribution

Corresponding authors: Manuel Rodríguez-Paredes (m.rodriguez@dkfz.de, +49 6221424631)

Frank Lyko (f.lyko@dkfz.de, +49 6221423800)

Table of Contents

Appendix Figure S1. Single-cell transcriptomics of healthy human epidermis identifies archetypical keratinocyte populations. 3

Appendix Figure S2. Main genes involved in keratinocyte differentiation identified by RNA velocity. 4

Appendix Figure S3. Tumor subclasses do not display differences in sample purity. 5

Appendix Figure S4. Single-cell methylation of human epidermis shows variable methylomes. . 6

Appendix Figure S6. Illustrative examples of FFPE tumor isolation by laser microdissection. 8

Appendix Figure S7. Comparison between epidermal differentiation-related regulatory regions identified in vitro and in vivo. 9

Appendix Table S1. Sequencing primers for sci-MET. 10

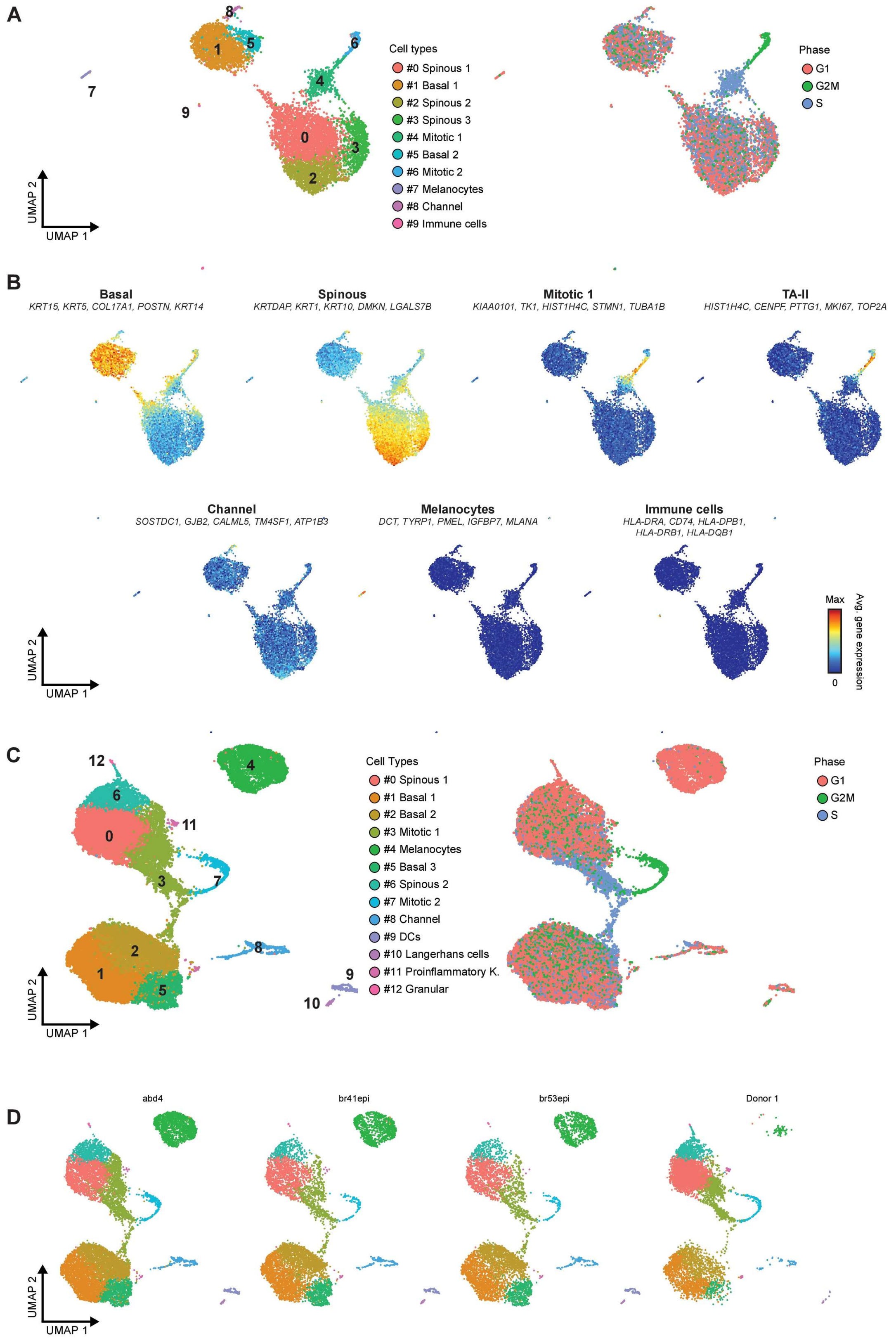
A
Cell types
#0 Spinous 1
#1 Basal 1
#2 Spinous 2
#3 Spinous 3
#4 Mitotic 1
#5 Basal 2
#6 Mitotic 2
#7 Melanocytes
#8 Channel
#9 Immune cells
Phase
G1
G2M
S
UMAP 2
UMAP 1
B
Basal
KRT15, KRT5, COL17A1, POSTN, KRT14
Spinous
KRTDAP, KRT1, KRT10, DMKN, LGALS7B
Mitotic 1
KIAA0101, TK1, HIST1H4C, STMN1, TUBA1B
TA-II
HIST1H4C, CENPF, PTTG1, MKI67, TOP2A
Channel
SOSTDC1, GJB2, CALML5, TM4SF1, ATP1B3
Melanocytes
DCT, TYRP1, PMEL, IGFBP7, MLANA
Immune cells
HLA-DRA, CD74, HLA-DPB1, HLA-DRB1, HLA-DQB1
Max
0
Avg. gene expression
C
Cell Types
#0 Spinous 1
#1 Basal 1
#2 Basal 2
#3 Mitotic 1
#4 Melanocytes
#5 Basal 3
#6 Spinous 2
#7 Mitotic 2
#8 Channel
#9 DCs
#10 Langerhans cells
#11 Proinflammatory K.
#12 Granular
Phase
G1
G2M
S
D
abd4
br41epi
br53epi
Donor 1

Appendix Figure S1. Single-cell transcriptomics of healthy human epidermis identifies archetypical keratinocyte populations.

A UMAP plot depicts 7,143 single-cell transcriptomes from a human healthy epidermis sample, generated in-house. Each cell is shown as a dot, and coloring is according to unsupervised clustering performed by Seurat (left) or cell cycle phase (right).

B UMAP plots show the average expression of the most representative genes of well-known human epidermal cell populations. Red indicates maximum average expression and blue indicates low or no expression in log-normalized UMI counts.

C UMAP plot depicts 32,272 single-cell transcriptomes from the integrated dataset comprising four healthy epidermal samples. Each cell is shown as a dot and coloring is according to unsupervised clustering performed by Seurat (left) or cell cycle phase (right).

D UMAP plots depict the contribution of each sample to the integrated dataset. Samples abd4, br41epi and br53epi correspond to the dataset published in Cheng *et al.*(Cheng *et al*, 2018) while Donor 1 corresponds to the in-house dataset.

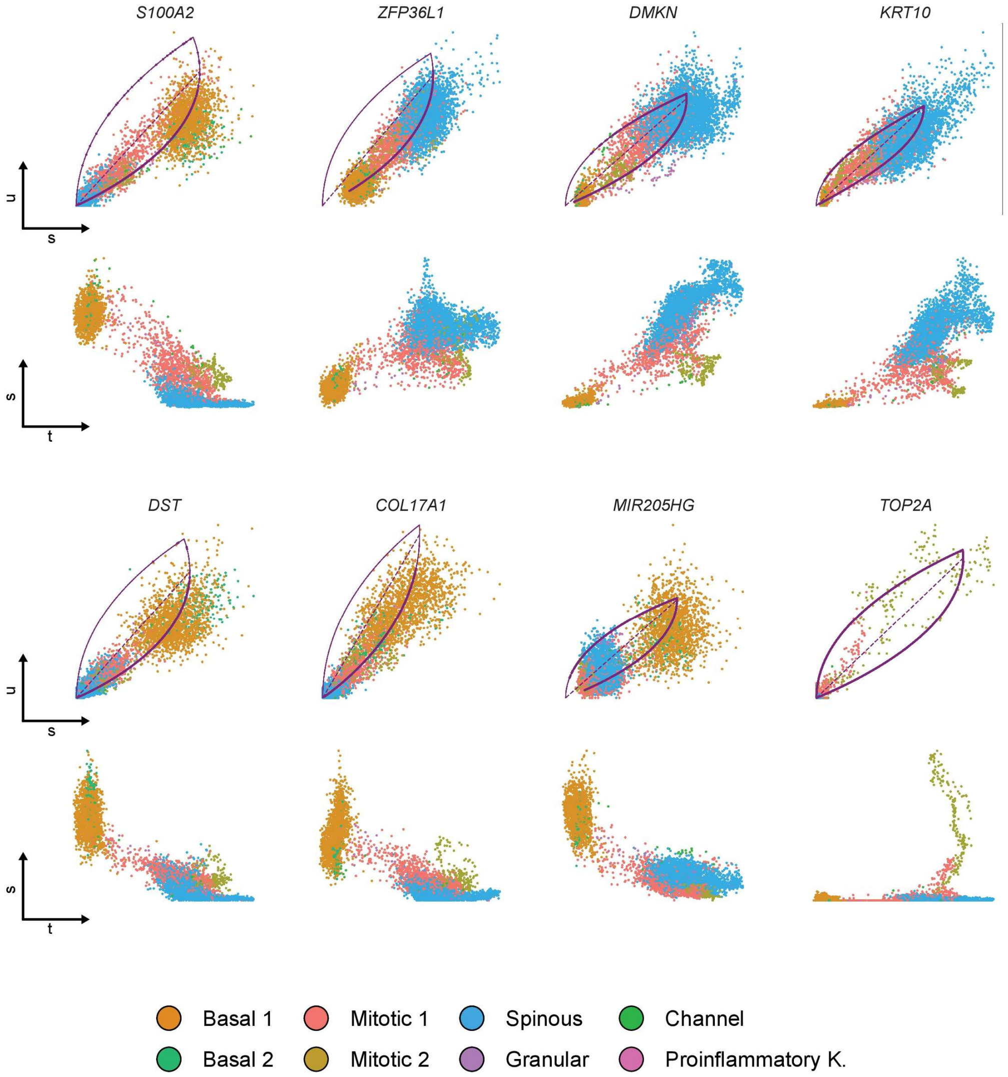

Appendix Figure S2. Main genes involved in keratinocyte differentiation identified by RNA velocity. Phase portraits (upper) and expression dynamics along latent time (lower) of the top eight putative driver genes of keratinocyte differentiation, as calculated by the dynamic model of scVelo(Bergen *et al*, 2020). Coloring is according to the defined keratinocyte populations. Bold lines depict the inferred splicing dynamics, while the dotted lines depict the transcriptional steady state. u: unspliced, s: spliced, t: latent time.

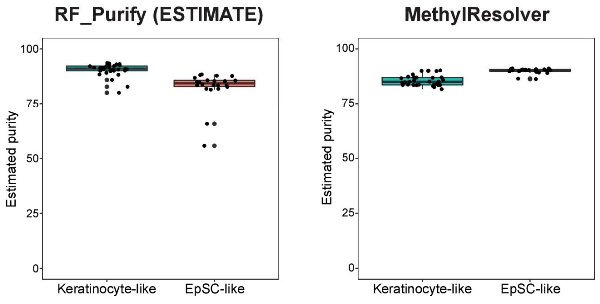

Appendix Figure S3. Tumor subclasses do not display differences in sample purity. Boxplot showing the tumor purity of the 55 AK/cSCC samples, as estimated by RF_Purify (ESTIMATE method) and MethylResolver.

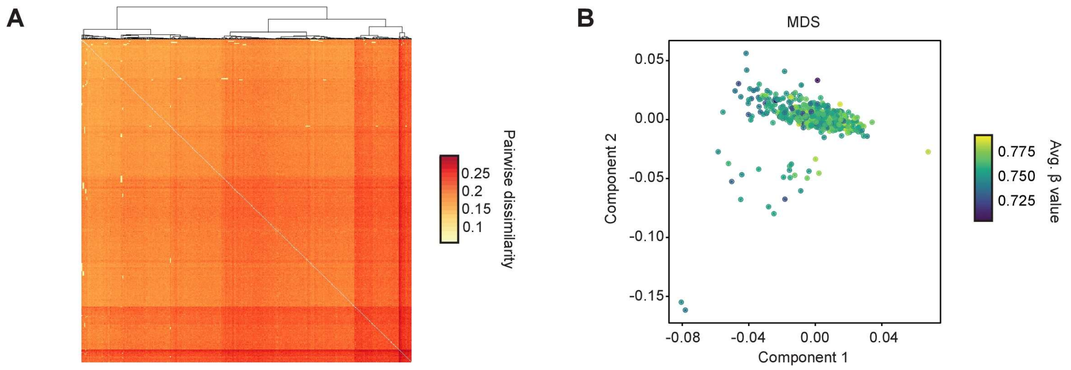

Appendix Figure S4. Single-cell methylation of human epidermis shows variable methylomes.

A Pairwise dissimilarity matrix between the 554 single-cell methylomes obtained from healthy human epidermis.

B Multidimensional scaling (MDS) analysis of the 554 single-cell methylomes obtained from healthy human epidermis and colored by average methylation content per cell.

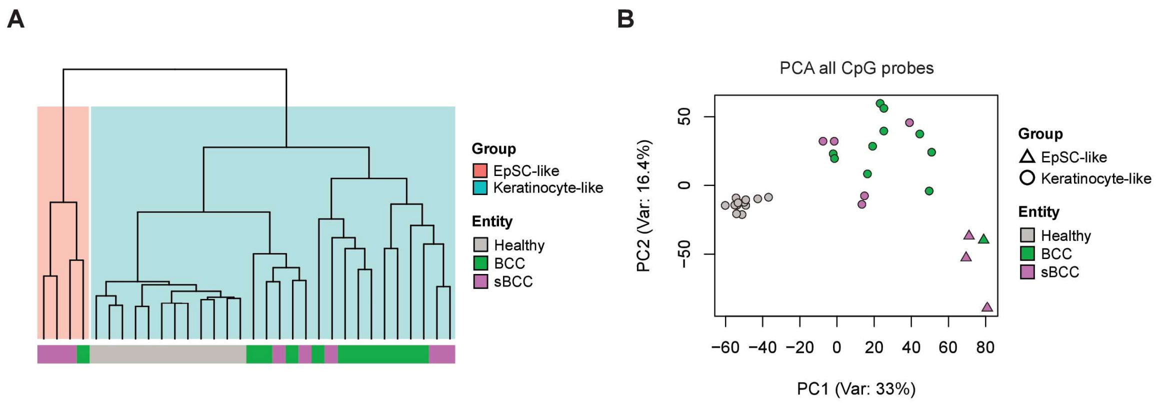

Appendix Figure S5. Combined analysis of BCC and sBCC tumor samples.

A Heatmap displaying the unsupervised hierarchical clustering of the 14 BCC (11 BCC and three sBCC) samples from our dataset and five sBCC from Sand *et al.*(Sand *et al*, 2019) based on the methylation patterns at differentiated keratinocyte-specific peaks. Healthy epidermal samples (n=12) were included as controls.

B PCA performed with 12 healthy epidermis, 11 BCC and eight sBCC samples using all CpG probes retained after filtering. Coloring is according to sample type and shape is according to cell-of-origin subclass. BCC: basal cell carcinoma, sBCC: sclerodermiform basal cell carcinoma.

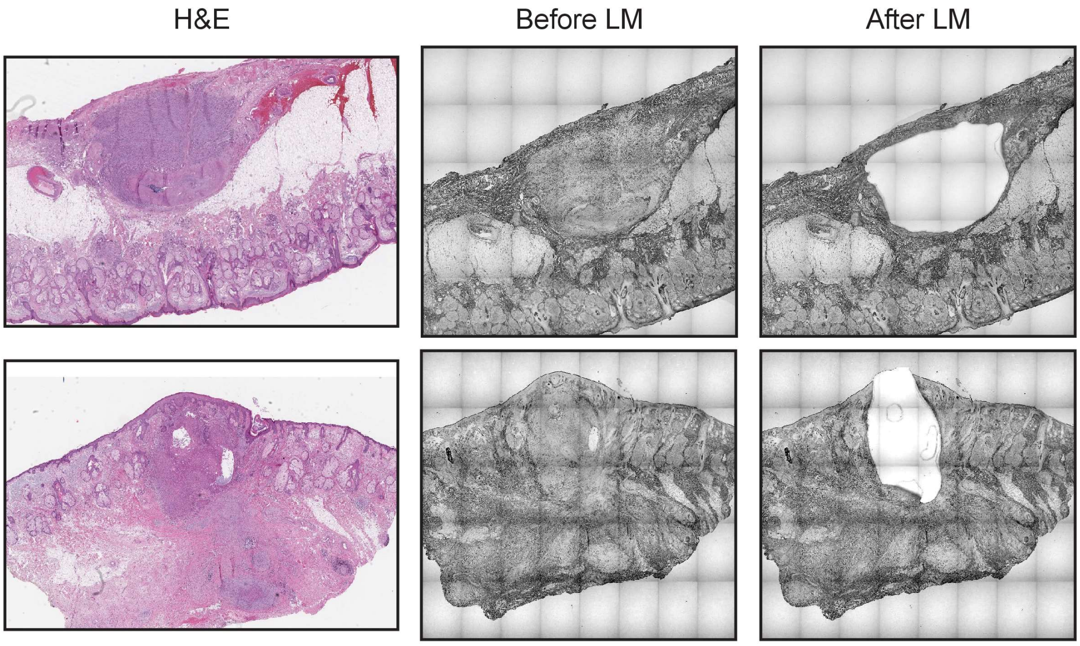

Appendix Figure S6. Illustrative examples of FFPE tumor isolation by laser microdissection. For each example, a hematoxylin and eosin (H&E) staining is shown (left), as well as an image of the tumor section before (middle) and after (right) laser microdissection. Images were taken at 5x original magnification. H&E: hematoxylin and eosin, LM: laser microdissection.

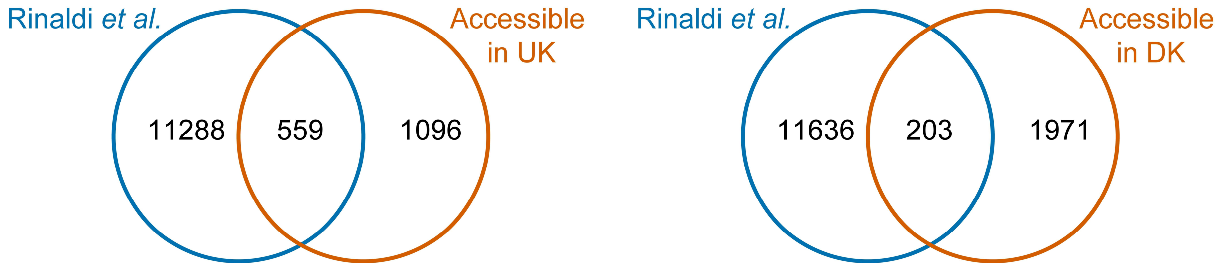

Appendix Figure S7. Comparison between epidermal differentiation-related regulatory regions identified in vitro and in vivo. Venn diagram comparing the set of undifferentiated and differentiated-specific peaks identified in our *in vivo* scATAC-seq data and a previously published set of enhancers found to be lost upon epidermal differentiation (Rinaldi *et al*, 2016), identified in upon calcium-induced differentiation of EpSC *in vitro*. DK: differentiated keratinocytes; UK: undifferentiated keratinocytes.

Appendix Table S1. Sequencing primers for sci-MET. Primer sequences for sequencing of sci-MET libraries. +: Locked Nucleic Acid (LNA) modification.

Name	Sequence
sciMET_Read1	+TGGT+AGAG+AGGG+TGAGATGTGTATAAGAGATAG
sciMET_Index1	AGATCGGAAGAGCACACGTCTGAACTCCAGTCAC
sciMET_Index2	+CTA+TCT+CTT+ATA+CAC+ATC+TCACCCTCTCTACCA
PhiX_Read1	ACACTCTTTCCCTACACGACGCTCTTCCGATCT
PhiX_Index1	AGATCGGAAGAGCGTCGTGTAGGGAAAGAGTGT

References

Bergen V, Lange M, Peidli S, Wolf FA & Theis FJ (2020) Generalizing RNA velocity to transient cell states through dynamical modeling. *Nat Biotechnol* 38: 1408–1414

Cheng JB, Sedgewick AJ, Finnegan AI, Benz SC, Song JS & Cho RJ (2018) Transcriptional Programming of Normal and Inflamed Human Epidermis at Single-Cell Resolution. *Cell Rep* 25: 871–883

Rinaldi L, Datta D, Serrat J, Morey L, Solanas G, Avgustinova A, Blanco E, Pons JI, Matallanas D, von Kriegsheim A, *et al* (2016) Dnmt3a and Dnmt3b Associate with Enhancers to Regulate Human Epidermal Stem Cell Homeostasis. *Cell Stem Cell* 19: 491–501

Sand M, Hessam ABDSTGS, Becker JC, Meyer EST & Bechara FG (2019) Dicer sequencing, whole genome methylation profiling, mRNA and smallRNA sequencing analysis in basal cell carcinoma. *Cell Physiol Biochem* 53: 760–773
