# Supplementary material for: Differentiation‐related epigenomic changes define clinically distinct keratinocyte cancer subclasses
Source: Mol Syst Biol. 2022 Sep 19;18(9):e11073. doi: 10.15252/msb.202211073 (PMC9484266; doi:10.15252/msb.202211073)
Supplement: Supplementary file 7 [file MSB-18-e11073-s004.pdf]

Differentiation-related epigenomic changes define clinically distinct keratinocyte cancer subclasses

Llorenç Solé-Boldo, Günter Raddatz, Julian Gutekunst, Oliver Gilliam, Felix Bormann, Michelle Liberio, Daniel Hasche, Wiebke Antonopoulos, Jan-Philipp Mallm, Anke Lonsdorf, Manuel Rodríguez-Paredes and Frank Lyko
DOI: 10.15252/msb.202211073

Corresponding author(s): Manuel Rodríguez-Paredes (m.rodriguez@dkfz.de) , Frank Lyko (f.lyko@dkfz.de)

Review Timeline:

Submission Date:	13th Apr 22
Editorial Decision:	12th May 22
Revision Received:	19th Jul 22
Editorial Decision:	12th Aug 22
Revision Received:	17th Aug 22
Accepted:	30th Aug 22

Editor: Jingyi Hou

Transaction Report:

(Note: With the exception of the correction of typographical or spelling errors that could be a source of ambiguity, letters and reports are not edited. Depending on transfer agreements, referee reports obtained elsewhere may or may not be included in this compilation. Referee reports are anonymous unless the Referee chooses to sign their reports.)

1st Editorial Decision 12th May 2022

Thank you for submitting your work to Molecular Systems Biology. We have now heard back from two reviewers who agreed to evaluate your study. As you will see from the comments below, the reviewers find the study of potential interest. However, they raise a number of concerns about your work, which should be convincingly addressed in a major revision of the present manuscript.

I think that the reviewers' recommendations are rather clear, and there is no need to reiterate their comments. All issues raised by the reviewers need to be satisfactorily addressed. As you may already know, our editorial policy allows in principle a single round of major revision, and it is therefore essential to provide responses to the reviewers' comments that are as complete as possible.

On a more editorial level, we would ask you to address the following issues:

Reviewer #1:

The paper "Differentiation-related epigenomic changes define clinically distinct keratinocyte cancer subclasses" by Bole-Soldo et al presents a multi-omic single cell and bulk-tissue analysis of healthy epidermal and keratinocyte cancers (KCs) (including both basal cell carcinomas-BCCs and cutaneous squamous cell carcinoma- cSCC). Besides scRNA-Seq, the authors also do profiling of the epigenome (scATAC-Seq + DNAm) using joint profiling techniques (e.g. sci-MET). The authors identify two distinct subclasses of KCs and using the single-cell data generated from healthy epidermal samples, are able to relate these subclasses to two distinct cells of origin, effectively one subclass with more stem-like features and another derived from a more differentiated cell-type. The authors provide some evidence that the stem-like subtype is more invasive.
I think that overall this is well-conducted study using a rich repertoire of technologies and statistical methods to arrive at an important novel insight in KC. Indeed, dissecting the cells of origin of cancers generally and how they relate to molecular classifications is an important area of research. As such, this paper advances our molecular understanding of KCs and their preinvasive lesions. Paper is well written and figures are presented to a professional standard. However, whether this paper is appropriate for a journal like MSB, which focuses more on systems biology, is questionable, as I did not perceive the novel systems biological insights. The paper is very descriptive. I would have thought that this manuscript would be much more suitable for a cancer or genome medicine focused journal like Cancer Research, Cancer Discovery, Genome Medicine or Cancer Cell. Indeed, are the authors not doing themselves a disfavour by trying to publish this in a journal which is not cancer-based?
Besides this, there are only a number of relatively minor but still important issues that should be addressed:
1) Differentially accessible peaks: I was a little bit struck by the fact that the authors identified more differentially accessible peaks as being accessible in differentiated keratinocytes (2179) than in undifferentiated keratinocytes (1,659) when later in the DNA methylation analysis the authors report twice as many EPIC probes in accessible peaks within undifferentiated keratinocytes compared to differentiated ones, with the number of overlapping peaks also greater for undifferentiated keratinocytes. Can the authors please explain how this huge switch in numbers can happen? Is it due to some underlying bias of the EPIC array? i.e. why would it be that probes within accessible peaks in differentiated keratinocytes are underrepresented on the EPIC array? Please check.
2) Only 2 EpSC-like cells from Sci-MET: it is undeniable that the number of cells (n=185) with sufficient coverage obtained from sci-MET is really small, specially the number of EpSC-like cells which was only 2. Could the authors somehow relax their analyses to ensure a greater number of cells please? Should the authors perhaps not process additional samples? It would be good if the authors could obtain at least 10-50 EpSC cells if possible.
3) Datasets are a valuable resource that should be made publicly available (with no restrictions): it is a shame that the authors have not made their data publicly available. They state they will do this upon revising the MS. I would strongly urge the authors to deposit all the data including processed normalized scRNA-Seq, DNA methylation and scATAC-Seq data in a truly open repository such as GEO or figshare (ie *not EGA* because EGA requires a formal application process), in order to make this a resource that other groups can *easily* use. Only by being open and transparent can science move forward. These datasets are rich and complex, and it only benefits mankind the more people can analyze them.

Reviewer #2:

The study by Sole-Boldo and colleagues aimed to characterise the epigenomic dynamics underlying human keratinocyte differentiation in order to uncover the cells-of-origin of keratinocyte cancers.

They report that keratinocyte cancers enriched in cells classified, as epithelial stem cells tend to have a more aggressive clinical phenotype indicating that the cells-of-origin of the corresponding keratinocyte cancer could be exploited as prognostic markers.

For the purposes of this study they have used single cell technologies (single cell RNA-seq, multiome that combines scATAC-seq and scRNA-seq on the same cells, single cell DNA methylation) to profile epidermis samples from healthy individuals. This dataset was integrated with published data including scRNA-seq data from epidermis of healthy individuals (Cheng et al 2018), ChIP-seq data for histone marks on normal epidermal keratinocytes (ENCODE project), as well as DNA methylation profiles (EPIC-array) from healthy epidermis and keratinocyte cancers.

This could be a potentially interesting study; defining the exact cell-of-origin of cancers is important not only in understanding tumor biology but could potentially explain patient heterogeneity, contribute to better design of targeted therapies and used as prognostic markers. Hence, studies as this one should be considered of high relevance. Importantly, recent advances in single cell technologies interrogating the epigenome are instrumental in defining cellular states.

However, the way this study was designed and presented is not very coherent and it is not always easy to follow. The inclusion of samples and technologies used is not always justified. In addition, parts of the analysis (especially in Figure 1) are not novel and is not clear if they add anything to this study.
Specific comments (not in particular order):

1.scRNA-seq analysis (Figure 1). It is not clear why the authors analysed one epidermis sample from a healthy individual. They could have used only the published data from Cheng et al 2018, which they included in their analysis anyway. In addition, in the Cheng et al study they profiled cells from the foreskin, trunk, and scalp. How important is the location from where epidermis is isolated? This is not discussed in the manuscript. Also, how about the donor age?

2.scRNA-seq analysis on normal epidermis has been reported previously in at least two studies (Cheng 2018, Ji 2020). How does the analysis presented in Figure 1 compares to the analysis of previously published studies?

3.Multiome analysis - this could have been the most exciting part of this study. But somehow this data are not comprehensively discussed and analysed. The authors could have dedicated the whole figure 1 on this; instead they only show two genome browser snap shots, just confirming that the scATA-seq protocol worked. As a suggesting the authors could move the first part of Fig 2. (Fig 2A-D) to Figure 1 - as it is presented, this analysis is lost as authors seem to highlight mostly DNA methylation analysis.

4.Fig 2A does not add anything to the study - could be omitted or removed to supplemental information section. Same applies to Fig 2C - it just confirms that scATAC-seq peaks correspond to region with open chromatin.

5.It is not clear if DNA methylation data (12 healthy, 20 AK, 35 sSCC) used in Figures 2 have been previously published or generated as part of this study.

6.sci-MET - it is not clear if performing single cell DNA methylation analysis was really crucial for this analysis. How does it compare to scATAC-seq (given that it allows interrogation of thousands of cells). How the 186 cells were selected?

7.A cluster comprising only two cells is hard to be evaluated. It is even surprising that the authors make a big deal out of it. They have to explain how confident they are about this analysis. It is hard to trust this data. Although, we have to recognize that single cell methylation analysis it is not a trivial task.

8.Which data set has been used for calculating mitotic age and proliferative rate of cells?

9.Fig4C on microRNAs comes rather sudden and without a strong connection to the analysis discussed above.

10.Where do DNA methylation profiles used for Fig 5 come from? Have they been generated for the purposes of this study?

11.p-values are missing from many figures.

Reviewer #1:

1)Differentially accessible peaks: I was a little bit struck by the fact that the authors identified more differentially accessible peaks as being accessible in differentiated keratinocytes (2179) than in undifferentiated keratinocytes (1,659) when later in the DNA methylation analysis the authors report twice as many EPIC probes in accessible peaks within undifferentiated keratinocytes compared to differentiated ones, with the number of overlapping peaks also greater for undifferentiated keratinocytes. Can the authors please explain how this huge switch in numbers can happen? Is it due to some underlying bias of the EPIC array? i.e. why would it be that probes within accessible peaks in differentiated keratinocytes are underrepresented on the EPIC array? Please check.

The discrepancy in numbers can be explained by the fact that accessible regions in undifferentiated keratinocytes cover more DNA sequence compared to the accessible regions in differentiated keratinocytes (see the attached figure below). More specifically, the average peak length in undifferentiated keratinocytes is 547.6 bp while the average peak length in differentiated keratinocytes is 423.6 bp.

Furthermore, the probes contained in the Infinium EPIC arrays are enriched for CpGs located in promoter regions whereas only 16% of the EPIC probes cover intergenic regions (Pidsley *et al*, 2016). Our results indicate an enrichment for enhancers (usually located in intergenic regions) in the peaks associated with differentiated keratinocytes. This bias in the EPIC design might also contribute to the discrepancy in the amount of CpGs detected. This point has been clarified in the Materials and Methods section.

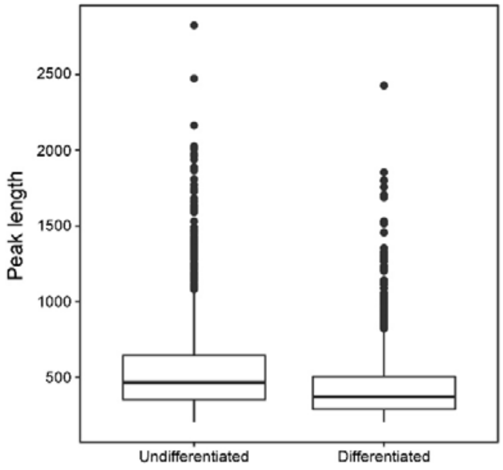

2) Only 2 EpSC-like cells from Sci-MET: it is undeniable that the number of cells (n=185) with sufficient coverage obtained from sci-MET is really small, specially the number of EpSC-like cells which was only 2. Could the authors somehow relax their analyses to ensure a greater number of cells please? Should the authors perhaps not process additional samples? It would be good if the authors could obtain at least 10-50 EpSC cells if possible.

We have now processed two more libraries containing around 200 cells each. A total of 554 epidermal cells passed our stringent quality controls which now includes 6 EpSCs (see our revised Fig. 3A). This also allowed us to further confirm the previously defined methylation patterns in the distinct genomic regions (see our revised Fig. 3B).

3) Datasets are a valuable resource that should be made publicly available (with no restrictions): it is a shame that the authors have not made their data publicly available. They state they will do this upon revising the MS. I would strongly urge the authors to deposit all the data including processed normalized scRNA-Seq, DNA methylation and scATAC-Seq data in a truly open repository such as GEO or figshare (ie *not EGA* because EGA requires a formal application process), in order to make this a resource that other groups can *easily* use. Only by being open and transparent can science move forward. These datasets are rich and complex, and it only benefits mankind the more people can analyze them.

All newly generated datasets have now been uploaded to the GEO database (accession no. GSE207337) and the ArrayExpress database (accession no. E-MTAB-11856). This has been clarified in the manuscript and a reviewer access is available through the following links:

- GEO database: https://www.ncbi.nlm.nih.gov/geo/query/acc.cgi?acc=GSE207337
  reviewer token: uhivuuwcfpcfvon
- ArrayExpress database: https://www.ebi.ac.uk/arrayexpress/experiments/E-MTAB-11856
  Username: Reviewer_E-MTAB-11856
  Password: q9ME29ac

Reviewer #2:

1.scRNA-seq analysis (Figure 1). It is not clear why the authors analysed one epidermis sample from a healthy individual. They could have used only the published data from Cheng et al 2018, which they included in their analysis anyway. In addition, in the Cheng et al study they profiled cells from the foreskin, trunk, and scalp. How important is the location from where epidermis is isolated? This is not discussed in the manuscript. Also, how about the donor age?

We generated our own dataset to be able to run certain analyses (i.e. RNA velocity) that could not be performed with the pre-processed published data. To ensure that our dataset was properly annotated and to gain statistical power in analyses that benefit from higher cell numbers (i.e. EpiSCORE) we specifically integrated it with three published samples from a comparable location (trunk area). These points have now been clarified in the Materials and Methods and Results sections of the manuscript.

Donor age was not provided in the original publication (Cheng *et al*, 2018). In any case, our integrated analysis showed no major differences between the four samples and all cell clusters were similarly present in all samples (see Appendix Fig S1D).

2.scRNA-seq analysis on normal epidermis has been reported previously in at least two studies (Cheng 2018, Ji 2020). How does the analysis presented in Figure 1 compares to the analysis of previously published studies?

Ji et al. analyzed epidermis obtained from healthy and cSCC samples (Ji *et al*, 2020). While they classified healthy cells as basal, cycling and differentiated, they did not consider subtypes (for instance spinous, granular, or other more specialized keratinocytes). Cheng et al. 2018 described keratinocyte heterogeneity in higher detail,

and we have recapitulated the original analysis of Cheng et al in our manuscript with a more up-to-date computational pipeline. This is now clarified in the Discussion section of the manuscript.

Cheng et al also used pseudotime and lineage inference to further characterize epidermal differentiation (Cheng *et al*, 2018). They used the graph-based tool Slingshot based on PCA components which identified several branches that were left poorly characterized. On the contrary, our RNA velocity analysis, which is not graph-based but transcriptome-based, shows only one clear differentiation branch. This has been clarified in the revised discussion.

3.Multiome analysis - this could have been the most exciting part of this study. But somehow this data are not comprehensively discussed and analysed. The authors could have dedicated the whole figure 1 on this; instead they only show two genome browser snap shots, just confirming that the scATA-seq protocol worked. As a suggesting the authors could move the first part of Fig 2. (Fig 2A-D) to Figure 1 - as it is presented, this analysis is lost as authors seem to highlight mostly DNA methylation analysis.

We have followed the reviewer's recommendation and we have considerably expanded the multiome analysis to comprehensively analyze epidermal differentiation. Thus, we have now investigated the co-accessibility at the human epidermal differentiation complex, and also identified the main transcription factors involved in keratinocyte differentiation, which has revealed novel potential regulators. Figure 1 is now entirely dedicated to the multiome dataset, while the reference scRNA-seq dataset analysis has been moved to the Appendix and the Expanded View Figures.

4.Fig 2A does not add anything to the study - could be omitted or removed to supplemental information section. Same applies to Fig 2C - it just confirms that scATAC-seq peaks correspond to region with open chromatin.

Fig 2A has been now integrated in Fig 1D. Together with the newly added analyses, this figure contributes to the identification of known and novel regulators of human epidermal differentiation.

Fig 2C corresponds now to Fig 2A. With this analysis we identified that differentially accessible peaks are enriched for histone marks associated with regulatory regions (promoters and enhancers). Importantly, these regions are the most dynamically methylated upon cell differentiation and can be leveraged to investigate a tumor's cell-of-origin. These results therefore prompted us to investigate the methylation patterns in such peaks in the context of skin cancer.

5.It is not clear if DNA methylation data (12 healthy, 20 AK, 35 sSCC) used in Figures 2 have been previously published or generated as part of this study.

Some datasets were generated for this study, while others were published previously. This has now been clarified in the manuscript.

6.sci-MET - it is not clear if performing single cell DNA methylation analysis was really crucial for this analysis. How does it compare to scATAC-seq (given that it allows interrogation of thousands of cells). How the 186 cells were selected?

Our cell-of-origin hypothesis for the keratinocyte cancers was based on bulk DNA methylation data and not on chromatin accessibility. Thus, performing single-cell DNA methylation was indeed crucial to provide a direct confirmation for this hypothesis that could not have been obtained otherwise. We have now further validated this hypothesis with the newly sequenced sci-MET libraries (see our revised Fig. 3A-C).

Regarding the 186 epidermal cells (554 after our revision of the manuscript), they were selected after sequencing. One of the main limitations in single-cell methylomics techniques is the low coverage of CpGs per cell. To minimize this limitation, we reduced the number of cells included in each sequencing round, and only those for which we obtained a minimum of 100,000 sequencing reads were retained for further analyses. By doing this, we observed a CpG coverage per cell comparable to the one reported in the original sci-MET publication, and that was also sufficient for cell type identification (Mulqueen *et al*, 2018).

7.A cluster comprising only two cells is hard to be evaluated. It is even surprising that the authors make a big deal out of it. They have to explain how confident they are about this analysis. It is hard to trust this data. Although, we have to recognize that single cell methylation analysis it is not a trivial task.

We have sequenced additional libraries to triple the number of cells and to strengthen our analysis (see our revised Fig. 3A). Also see reviewer 1, point 2.

8.Which data set has been used for calculating mitotic age and proliferative rate of cells?

The bulk methylation (EPIC) dataset comprising 12 healthy, 20 AK and 35 cSCC epidermal samples. This has been clarified in the manuscript.

9.Fig4C on microRNAs comes rather sudden and without a strong connection to the analysis discussed above.

We hypothesized that a less proliferative phenotype in EpSC-like tumors could correlate with a more invasive phenotype, as these two processes are anticorrelated in cancer cells. The miRNAs analyzed are known to silence important transcription factors (ZEB1 and ZEB2) driving the epithelial-to-mesenchymal transition, an essential process for cancer cells to become invasive. The promoters of these miRNAs have been found hypermethylated in a wide range of invasive cell lines and human cancers.

This line of thought has now been clarified in the main text of the manuscript. Furthermore, we have performed immunofluorescence staining against ZEB2 in keratinocyte-like and EpSC-like cSCC samples (see our revised Fig. 4D). We found an increase in ZEB2-positive tumor cells in EpSC-like tumors, which provides important confirmation for our hypothesis.

10.Where do DNA methylation profiles used for Fig 5 come from? Have they been generated for the purposes of this study?

The dataset in Figure 5A contains the previously analyzed dataset containing 12 healthy, 20 AK and 35 cSCC, combined with newly generated samples including 14 BCC, 11 BD and 10 SK.

The datasets in Figure 5B, 5C and 5D were obtained from Sand et al 2019, Al Eitan et al 2020 and Hervás-Marín et al 2019, respectively.

The datasets in Figure 5E, corresponding to 8 cSCC metastases and 3 primary metastasizing cSCCs, were generated for this study.

These points have been clarified in the manuscript.

11.p-values are missing from many figures.

We have added p-values to all applicable figures.

References

Cheng JB, Sedgewick AJ, Finnegan AI, Benz SC, Song JS & Cho RJ (2018) Transcriptional Programming of Normal and Inflamed Human Epidermis at Single-Cell Resolution Article Transcriptional Programming of Normal and Inflamed Human Epidermis at Single-Cell Resolution. *Cell Rep* 25: 871–883

Ji AL, Rubin AJ, Thrane K, Jiang S, Reynolds DL, Meyers RM, Guo MG, George BM, Mollbrink A, Bergenstråhle J, *et al* (2020) Multimodal Analysis of Composition and Spatial Architecture in Human Squamous Cell Carcinoma. *Cell* 182: 497-514.e22

Mulqueen RM, Pokholok D, Norberg SJ, Torkenczy KA, Fields AJ, Sun D, Sinnamon JR, Shendure J, Trapnell C, O'Roak BJ, *et al* (2018) Highly scalable generation of DNA methylation profiles in single cells. *Nat Biotechnol* 36: 428–431

Pidsley R, Zotenko E, Peters TJ, Lawrence MG, Risbridger GP, Molloy P, van Djik S, Muhlhausler B, Stirzaker C & Clark SJ (2016) Critical evaluation of the Illumina MethylationEPIC BeadChip microarray for whole-genome DNA methylation profiling. *Genome Biol* 17:208

1st Revision - Editorial Decision 12th Aug 2022

Thank you for sending us your revised manuscript. We have now heard back from the two reviewers who agreed to evaluate your study. As you will see, the reviewers are overall satisfied with the modifications made.

Before we can formally accept your manuscript, we would ask you to address the following editorial-level issues:

Reviewer #1:

The authors have responded satisfactorily to my concerns. The only remaining issue, mostly an editorial issue, is that the new data deposited in GEO will only be released to the public on 22 July 2023, which is 1 year from now. Likewise, the link to the new data on ArrayExpress is not working, presumably because it is still private. I would advise the authors and editor to ensure data is open and freely accessible upon publication of this MS in MSB.

Reviewer #2:

The authors have adequately responded to reviewer comments.

2nd Revision - Editorial Decision 30th Aug 2022

Thank you again for sending us your revised manuscript. We are now satisfied with the modifications made and I am pleased to inform you that your paper has been accepted for publication.

EMBO Press Author Checklist

Corresponding Author Name: Manuel Rodríguez-Paredes & Frank Lyko
Journal Submitted to: Molecular Systems Biology
Manuscript Number: MSB-2022-11073

USEFUL LINKS FOR COMPLETING THIS FORM
The EMBO Journal - Author Guidelines
EMBO Reports - Author Guidelines
Molecular Systems Biology - Author Guidelines
EMBO Molecular Medicine - Author Guidelines

Reporting Checklist for Life Science Articles (updated January
This checklist is adapted from Materials Design Analysis Reporting (MDAR) Checklist for Authors. MDAR establishes a minimum set of requirements in transparent reporting in the life sciences (see Statement of Task: 10.31222/osf.io/9sm4x). Please follow the journal's guidelines in preparing your manuscript.
Please note that a copy of this checklist will be published alongside your article.

Abridged guidelines for figures
1. Data
The data shown in figures should satisfy the following conditions:
- the data were obtained and processed according to the field’s best practice and are presented to reflect the results of the experiments in an accurate and unbiased manner.
- ideally, figure panels should include only measurements that are directly comparable to each other and obtained with the same assay.
- plots include clearly labeled error bars for independent experiments and sample sizes. Unless justified, error bars should not be shown for technical
- if n<5, the individual data points from each experiment should be plotted. Any statistical test employed should be justified.
- Source Data should be included to report the data underlying figures according to the guidelines set out in the authorship guidelines on Data

2. Captions
Each figure caption should contain the following information, for each panel where they are relevant:
- a specification of the experimental system investigated (eg cell line, species name).
- the assay(s) and method(s) used to carry out the reported observations and measurements.
- an explicit mention of the biological and chemical entity(ies) that are being measured.
- an explicit mention of the biological and chemical entity(ies) that are altered/varied/perturbed in a controlled manner.
- the exact sample size (n) for each experimental group/condition, given as a number, not a range;
- a description of the sample collection allowing the reader to understand whether the samples represent technical or biological replicates (including how many animals, litters, cultures, etc.).
- a statement of how many times the experiment shown was independently replicated in the laboratory.
- definitions of statistical methods and measures:
  - common tests, such as t-test (please specify whether paired vs. unpaired), simple χ2 tests, Wilcoxon and Mann-Whitney tests, can be unambiguously identified by name only, but more complex techniques should be described in the methods section;
  - are tests one-sided or two-sided?
  - are there adjustments for multiple comparisons?
  - exact statistical test results, e.g., P values = x but not P values < x;
  - definition of ‘center values’ as median or average;
  - definition of error bars as s.d. or s.e.m.

Please complete ALL of the questions below.
Select "Not Applicable" only when the requested information is not relevant for your study.

Materials

Newly Created Materials	Information included in the manuscript?	In which section is the information available? (Reagents and Tools Table, Materials and Methods, Figures, Data Availability Section)
New materials and reagents need to be available; do any restrictions apply?	Not Applicable	

Antibodies	Information included in the manuscript?	In which section is the information available? (Reagents and Tools Table, Materials and Methods, Figures, Data Availability Section)
For **antibodies** provide the following information:<br>- Commercial antibodies: RRID (if possible) or supplier name, catalogue number and or/clone number<br>- Non-commercial: RRID or citation	Yes	Materials and Methods

DNA and RNA sequences	Information included in the manuscript?	In which section is the information available? (Reagents and Tools Table, Materials and Methods, Figures, Data Availability Section)
**Short novel DNA or RNA including primers, probes**: provide the sequences.	Yes	Appendix Table S1

Cell materials	Information included in the manuscript?	In which section is the information available? (Reagents and Tools Table, Materials and Methods, Figures, Data Availability Section)
**Cell lines:** Provide species information, strain. Provide accession number in repository **OR** supplier name, catalog number, clone number, and/**OR** RRID.	Not Applicable	
**Primary cultures:** Provide species, strain, sex of origin, genetic modification status.	Not Applicable	
Report if the cell lines were recently **authenticated** (e.g., by STR profiling) and tested for mycoplasma contamination.	Not Applicable	

Experimental animals	Information included in the manuscript?	In which section is the information available? (Reagents and Tools Table, Materials and Methods, Figures, Data Availability Section)
**Laboratory animals or Model organisms:** Provide species, strain, sex, age, genetic modification status. Provide accession number in repository **OR** supplier name, catalog number, clone number, **OR** RRID.	Not Applicable	
**Animal observed in or captured from the field:** Provide species, sex, and age where possible**.**	Not Applicable	
Please detail **housing and husbandry conditions**.	Not Applicable	

Plants and microbes	Information included in the manuscript?	In which section is the information available? (Reagents and Tools Table, Materials and Methods, Figures, Data Availability Section)
**Plants:** provide species and strain, ecotype and cultivar where relevant, unique accession number if available, and source (including location for collected wild specimens).	Not Applicable	
**Microbes:** provide species and strain, unique accession number if available, and source.	Not Applicable	

Human research participants	Information included in the manuscript?	In which section is the information available? (Reagents and Tools Table, Materials and Methods, Figures, Data Availability Section)
If collected and within the bounds of privacy constraints report on age, sex and gender or ethnicity for all study participants.	Yes	Main text, Materials and Methods

Core facilities	Information included in the manuscript?	In which section is the information available? (Reagents and Tools Table, Materials and Methods, Figures, Data Availability Section)
If your work benefited from core facilities, was their service mentioned in the acknowledgments section?	Yes	Materials and Methods

Design

Study protocol	Information included in the manuscript?	In which section is the information available? (Reagents and Tools Table, Materials and Methods, Figures, Data Availability Section)
If study protocol has been **pre-registered, provide DOI in the manuscript**. For clinical trials, provide the trial registration number **OR** cite DOI.	Not Applicable	
Report the **clinical trial registration number** (at ClinicalTrials.gov or equivalent), where applicable.	Not Applicable	

Laboratory protocol	Information included in the manuscript?	In which section is the information available? (Reagents and Tools Table, Materials and Methods, Figures, Data Availability Section)
Provide DOI OR other citation details if **external detailed step-by-step protocols** are available.	Yes	Materials and Methods

Experimental study design and statistics	Information included in the manuscript?	In which section is the information available? (Reagents and Tools Table, Materials and Methods, Figures, Data Availability Section)
Include a statement about **sample size** estimate even if no statistical methods were used.	Yes	Main text, Materials and Methods
Were any steps taken to minimize the effects of subjective bias when allocating animals/samples to treatment (e.g. **randomization procedure**)? If yes, have they been described?	Not Applicable	
Include a statement about **blinding** even if no blinding was done.	Not Applicable	
Describe **inclusion/exclusion criteria** if samples or animals were excluded from the analysis. Were the criteria pre-established?<br><br>If sample or data points were omitted from analysis, report if this was due to attrition or intentional exclusion and provide justification.	Not Applicable	
For every figure, are **statistical tests** justified as appropriate? Do the data meet the assumptions of the tests (e.g., normal distribution)? Describe any methods used to assess it. Is there an estimate of variation within each group of data? Is the variance similar between the groups that are being statistically compared?	Yes	Main text, Figure legends, Materials and Methods

Sample definition and in-laboratory replication	Information included in the manuscript?	In which section is the information available? (Reagents and Tools Table, Materials and Methods, Figures, Data Availability Section)
In the figure legends: state number of times the experiment was **replicated** in laboratory.	Yes	Figure legends, Materials and Methods
In the figure legends: define whether data describe **technical or biological replicates**.	Yes	Figure legends, Materials and Methods

Ethics

Ethics	Information included in the manuscript?	In which section is the information available? (Reagents and Tools Table, Materials and Methods, Figures, Data Availability Section)
Studies involving **human participants**: State details of **authority granting ethics approval** (IRB or equivalent committee(s), provide reference number for approval.	Yes	Materials and Methods
Studies involving **human participants**: Include a statement confirming that **informed consent** was obtained from all subjects and that the experiments conformed to the principles set out in the WMA Declaration of Helsinki and the Department of Health and Human Services Belmont Report.	Yes	Materials and Methods
Studies involving **human participants:** For publication of **patient photos**, include a statement confirming that consent to publish was obtained.	Not Applicable	
Studies involving experimental **animals**: State details of **authority granting ethics approva**l (IRB or equivalent committee(s), provide reference number for approval. Include a statement of compliance with ethical regulations.	Not Applicable	
Studies involving **specimen and field samples:** State if relevant **permits** obtained, provide details of authority approving study; if none were required, explain why.	Not Applicable	

Dual Use Research of Concern (DURC)	Information included in the manuscript?	In which section is the information available? (Reagents and Tools Table, Materials and Methods, Figures, Data Availability Section)
Could your study fall under dual use research restrictions? Please check biosecurity documents and list of **select agents and toxins** (CDC): https://www.selectagents.gov/sat/list.htm	Not Applicable	
If you used a select agent, is the security level of the lab appropriate and reported in the manuscript?	Not Applicable	
If a study is subject to dual use research of concern regulations, is the name of the **authority granting approval and reference number** for the regulatory approval provided in the manuscript?	Not Applicable	

Reporting

The MDAR framework recommends adoption of discipline-specific guidelines, established and endorsed through community initiatives. Journals have their own policy about requiring specific guidelines and recommendations to complement MDAR.

Adherence to community standards	Information included in the manuscript?	In which section is the information available? (Reagents and Tools Table, Materials and Methods, Figures, Data Availability Section)
State if relevant guidelines or checklists (e.g., **ICMJE, MIBBI, ARRIVE, PRISMA**) have been followed or provided.	Not Applicable	
For **tumor marker prognostic studies,** we recommend that you follow the **REMARK** reporting guidelines (see link list at top right). See author guidelines, under 'Reporting Guidelines'. Please confirm you have followed these guidelines.	Not Applicable	
For **phase II and III randomized controlled trials**, please refer to the **CONSORT** flow diagram (see link list at top right) and submit the CONSORT checklist (see link list at top right) with your submission. See author guidelines, under 'Reporting Guidelines'. Please confirm you have submitted this list.	Not Applicable	

Data Availability

Data availability	Information included in the manuscript?	In which section is the information available? (Reagents and Tools Table, Materials and Methods, Figures, Data Availability Section)
Have **primary datasets** been deposited according to the journal's guidelines (see 'Data Deposition' section) and the respective accession numbers provided in the Data Availability Section?	Yes	Data Availability Section
Were **human clinical and genomic datasets** deposited in a public access-controlled repository in accordance to ethical obligations to the patients and to the applicable consent agreement?	Yes	Data Availability Section
Are **computational models** that are central and integral to a study available without restrictions in a machine-readable form? Were the relevant accession numbers or links provided?	Not Applicable	
If publicly available data were reused, provide the respective **data citations in the reference list**.	Yes	Main text, Reference list
